# A multicentre, multi-national, double-blind, randomised, active-controlled, parallel-group clinical study to assess the safety and efficacy of PDA10 (Epoetin-Alfa) vs. Eprex® in patients with anaemia of chronic renal failure

**DOI:** 10.1186/s12882-021-02601-w

**Published:** 2021-11-25

**Authors:** Soo Kun Lim, Bak Leong Goh, Ravindran Visvanathan, Su Hyun Kim, Jin Seok Jeon, Sung Gyun Kim, Jae Hyun Chang, Chun Soo Lim, Zaki Morad

**Affiliations:** 1 Renal Division, Department of Medicine, Faculty of Medicine, University of Malaya, 59100 Kuala Lumpur, Malaysia; 2 Serdang Hospital, Kajang, Malaysia; 3 Prince Court Medical Centre, Kuala Lumpur, Malaysia; 4 Chung-Ang University Hospital, Seoul, South Korea; 5 Soonchunhyang University Hospital, Seoul, South Korea; 6 Hallym University Sacred Heart Hospital, Anyang, South Korea; 7 Gil Medical Centre, Gachon University College of Medicine, Incheon, South Korea; 8 Seoul National University Boramae Medical Centre, Seoul, South Korea; 9 KPJ Ampang Puteri Specialist Hospital, Ampang, Malaysia

**Keywords:** Anaemia, Epoetin-α, Haemodialysis, PDA10, Therapeutic equivalence

## Abstract

**Background:**

Erythropoietin stimulating agent (ESA) has been standard of care in treating renal anaemia for the past 20 years. Many patients have limited access to ESA in view of long-term costs leading to suboptimal ESA dosage. Biosimilar epoetin is a potential cost-effective alternative to originator for optimal renal anaemia management.

**Objective:**

To determine efficacy and safety of PDA10 in treating renal anaemia in haemodialysis patients, in comparison to the originator epoetin-α, Eprex®.

**Methods:**

A phase 3, multicentre, multi-national, double-blind, randomised, active-controlled and parallel group study conducted over 40 weeks in Malaysia and Korea. End stage kidney disease patients undergoing regular haemodialysis who were on erythropoietin treatment were recruited. The study has 3 phases, which included a 12-week titration phase, followed by 28-week double-blind treatment phase and 24-week open-label extension phase.

**Results:**

The PDA10 and Eprex® were shown to be therapeutically equivalent (*p* < 0.0001) with mean absolute change in haemoglobin from baseline of − 0.176 (± 0.91) g/dl and − 0.118 (± 1.114) g/dl, respectively. Weekly dose change was 10.01 IU/kg/week in PDA10 group and 10.30 IU/kg/week in Eprex® group, which has no significant difference. There were no significant differences in the safety profile between PDA10 and Eprex® groups.

**Conclusion:**

This study has confirmed the therapeutic equivalence between PDA10 and Eprex® in terms of efficacy, dosage requirement and safety profile in haemodialysis patients with renal anaemia.

**Trial registration:**

The study was registered with the National Medical Research Register (NMRR-13-400-16313). This study has been registered retrospectively with Clinical Research Information Service (CRiS), Republic of Korea on 25 March 2021.

**Supplementary Information:**

The online version contains supplementary material available at 10.1186/s12882-021-02601-w.

## Background

Anaemia, that is, haemoglobin (Hb) concentration of < 12 g/dl in men and < 11 g/dl in women [1], is a common complication in chronic kidney disease (CKD). As the renal function declines to estimated glomerular filtration rate (eGFR) of < 60 mL/min/1.73 m^2^, the prevalence of anaemia and its severity increases [1, 2]. Though the main pathophysiology of anaemia in CKD is erythropoietin deficiency, it is often multifactorial [1–3]. These include iron and other micronutritional deficiencies, uraemic toxicity, hyperparathyroidism and inflammatory mechanisms associated with CKD.

Risk factors for anaemia in CKD are gender, age, stage of CKD, serum albumin levels and calcium and phosphorus concentrations [2, 4]. Diabetes not only increases the risk of anaemia but also its severity in all CKD stages compared to those without diabetes [1, 4, 5]. Renal replacement therapy with haemodialysis (HD) in end stage kidney disease (ESKD) further increases the risk of anaemia. Reasons include dialyzer blood loss, clotted dialysis membranes, frequent blood sampling and surgical interventions of vascular access. The consequences of anaemia in ESKD patients are compromised quality of life and, increased incidence of heart failure, blood transfusion requirement and mortality risk [1].

Some indications for epoetin-containing medicinal products are anaemia in CKD, chemotherapy-induced anaemia and increasing production of autologous blood. Recombinant human erythropoietin (rhEPO) is proven beneficial in treating renal anaemia [6–9] and clinical trials [10–12] have indicated an acceptable degree of safety.

Since the introduction of Epogen, an epoetin alfa, erythropoietin stimulating agents (ESAs) are the standard of care (SOC) for renal anaemia for over 20 years [13]. As the recommended target Hb level is 10–11.5 g/dl, up to 80–90% of dialysis patients require ESAs. The use of ESA however, varies depending on a country’s healthcare financial structure (33.7% in Korea, 91% in Malaysia and up to 13.1% in the United States of America) [2, 14, 15]. Though correction of Hb levels is the SOC in renal anaemia, current understanding of its risks and benefits shows that under and over-treatment can have equally poor outcomes [16–18].

All currently available ESAs work through the same signalling pathway leading to gene activation promoting survival, differentiation, proliferation and maturation of red blood cells progenitors and precursors. As cost effectiveness is an important consideration in choice of ESAs, a model agent should at a reduced dose frequency, correct Hb levels evenly and maintain it within target range.

Biosimilars for ESA have more than 10 years of evidence since its approval and are almost exclusively biosimilars to epoetin-α. PDA10 is a biosimilar epoetin-α, indicated for the treatment of renal anaemia. Its active substance is a rhEPO of identical primary structure produced in Chinese Hamster Ovary cells (CHO-DG44). Phase 2 clinical study was waived from regulatory authorities, because of PDA10 has been proven its biosimilarity based on results of Phase 1, non-clinical studies and analytical similarity studies [19].

This study aims to determine the safety and efficacy of PDA10 in renal anaemia in HD patients, compared to the originator epoetin-α, Eprex®.

## Methods

This is a phase 3, multicentre, multi-national, double-blind, randomised, active-controlled and parallel group study to determine the bioequivalence of PDA10 compared to Eprex® in ESKD patients with anaemia. The study was conducted over 40 weeks in 29 sites across Malaysia and Korea, from October 2013 till April 2017. The study has obtained approval from National Medical Research Register Medical Research and Ethics Committee (NMRR-13-400-16,313) prior to study initiation. All methods were carried out in accordance with relevant guidelines and regulations. This study has been registered retrospectively with Clinical Research Information Service (CRiS), Republic of Korea on 25 March 2021.

### Study population

Anaemic ESKD patients on chronic HD between 18 to below 75 years were screened as prospective subjects. Written informed consent in line with Korean and Malaysian Good Clinical Practice was obtained prior to any study procedures.

Taking into account a 20% drop-out rate, a sample size of 316 subjects was planned to achieve sufficient power determining bioequivalence. Inclusion criteria were ESKD undergoing chronic HD for at least 3 months, through a functioning native arterio-venous fistula, at or within 5% of their dry weight during baseline period and on ESA treatment prior to screening. For female patients, those of childbearing age with negative pregnancy tests and on contraception or, post-menopausal were included. Patients needed to fulfil certain stability criteria in dosage of ESA; Hb, serum ferritin and transferrin saturation (TSAT) levels and frequency of HD per week prior to randomisation.

Other than the general mandatory exclusion criteria, patients with hyperkalaemia, epilepsy, malnutrition, current diagnosis of anaemia due to other causes and history of pure red cell aplasia were excluded. Patients with dialysis catheters or synthetic grafts were also excluded because of higher infection risk among this group of patients. Those with uncontrolled hypertension (pre-dialysis diastolic blood pressure of > 110 mmHg), severe hyperparathyroidism (i.e. parathyroid hormone levels > 150 pmol/l) or those who needed blood transfusions, within 12 weeks prior to randomisation were also excluded.

### Study design

**Fig. 1:**
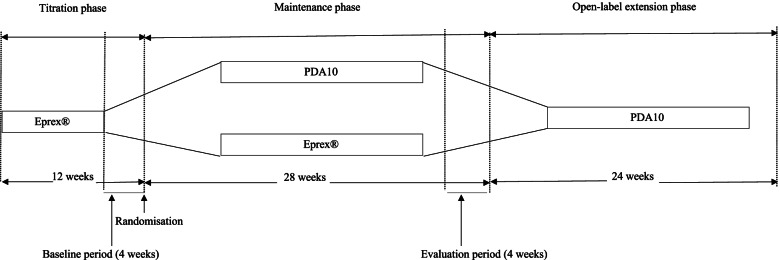
Study schedule. The study schedule was divided into three phases – Titration (12 weeks) which, included a 4-week baseline period at the end, Maintenance (28 weeks) which, included a 4-week evaluation period at the end and an open-label extension phase (24 weeks). Subjects were randomised at the end of the Titration phase

The study was divided into three phases (Fig. 1). The titration phase assessed the patients’ disease stability and established their baseline characteristics. In certain cases, this was prolonged up to 20 weeks. This included an observation (baseline) period during the last 4 weeks.

Patients already on Eprex® with a Hb level of < 10 g/dl during the screening period or those treated with other ESA for at least 12 weeks prior, had to participate in the titration phase. Those on Eprex® treatment for ≥12 weeks with a Hb level of 10–12 g/dl prior to titration phase and adequate HD thrice weekly with a documented urea reduction ratio > 65% or delivered Kt/V ≥ 1.2 required participation only in the 4-week baseline period. To be eligible for randomisation and entry into the following phase, all patients had to be on a stable intravenous (IV) Eprex® dose, had a stable Hb level between 10 and 12 g/dl and serum ferritin and/or TSAT levels of ≥100 ng/ml and ≥ 20% respectively, during the baseline period. A stable IV Eprex® dose was defined as not > 25% change in weekly dose and no clinically relevant change of HD regime during the baseline period. Titration of IV Eprex® dose (up- or down-titration of 25% of starting dose or, withholding of dose) was done 4-weekly based on the latest 2-weekly Hb levels. More frequent changes were made only when the value or rate of Hb change was outside the pre-set safety parameters of Hb < 10 g/dl or ≥ 12 g/dl or, increase of Hb by < 0.3 g/dl or > 1.0 g/dl every 2 weeks. All patients were also treated with iron therapy based on individual iron levels during this phase. A guideline for intravenous iron therapy is detailed in study protocol (Additional file 1).

The titration phase was followed by a 28-week double-blind treatment phase (maintenance phase) which included an evaluation period at the last 4 weeks. Eligible patients were randomised to receive one of the two study regimens (PDA10 or Eprex®) in a 1:1 ratio IV. During this phase, efficacy and safety were evaluated with Hb, haematocrit and IV Eprex® dose monitored 2-weekly and dose adjustments made, if required, every 4-weekly based on a pre-set scheme similar to the titration phase. A guideline for erythropoietin stimulating agent injections is detailed in study protocol (Additional file 1).

Subjects who completed the study at week 28 were offered a continuation of PDA10 treatment by entering a 24-week open-label extension phase for long-term safety and tolerability evaluation which is beyond the scope of this article.

### Treatments

PDA10 or Eprex® was supplied in pre-filled syringes (PFS) that had the same external appearance and functionality. During the titration phase all patients were administered Eprex® for the first 12 weeks. Those who met all randomisation criteria were randomised and treated with either the investigational product (PDA10 2000 IU/0.5 ml) or reference product (Eprex® 2000 IU/0.5 ml) for 28 weeks in a double-blind fashion.

Both treatments were administered as an IV bolus 1–3 times a week. The maximum dosage in the titration and maintenance phases could not exceed 300 IU/kg/dose. Only the investigational and reference treatments were allowed except during treatment of adverse events (AEs) and administration of other medications at the discretion of the principal investigators.

#### Statistical analysis

##### Sample size calculation

There are two co-primary endpoints in this study; the mean change in hemoglobin level and the mean change in weekly dose per kg body weight from baseline period to the evaluation period. A sample size of 126 per group was calculated to achieve 90% power to detect a difference in the mean change in hemoglobin level. In the case of weekly dose, it was calculated that a sample size of 97 per group is necessary to provide 90% power. The larger sample size of change in hemoglobin level (*n* = 126) was chosen for the trial, which is expected to give an overall 80% power for the proof of equivalence for primary endpoints. The total number of patients to be enrolled was estimated to be 316, including an assumed drop-out rate of 20%.

##### Efficacy endpoints

The primary endpoints were mean changes in Hb level and weekly dosage/kilogram (kg) body weight between baseline and evaluation periods. The Hb, haematocrit and treatment dosage for baseline period were collected at weeks-0, 2 and 4 and, at weeks-24, 26 and 28 during the evaluation period.

The secondary endpoints were mean change in haematocrit levels between baseline and evaluation periods, proportion of patients with Hb levels within and out of target range during evaluation and maintenance periods respectively, frequency of patients with changes in dosage, incidence of blood transfusions and safety endpoints (AEs, occurrence of anti-epoetin antibodies, vital signs, physical examination and clinical laboratory determinations).

##### Statistical methods

All analyses were performed using the data analysis software SAS® Version 9.3 at alfa= 0.05 level, two-sided, without any adjustment for multiple comparisons unless specified. The per protocol set (PPS) only included those completing the maintenance phase with no major protocol violations whilst the full analysis set (FAS) comprised of all subjects treated with study medication for > 4 weeks with ≥1 post-baseline value of the primary endpoints. Efficacy analyses were conducted in both sets with the PPS as the primary analysis set. The results of both sets were compared to draw final conclusion.

For primary endpoints, calculation of the upper and lower limits of 95% one-sided confidence intervals (CI) of the difference between both treatment groups were done. To demonstrate therapeutic equivalence, two one-sided tests (TOST) procedures were used where, d was the difference of the mean change in each endpoint (d_L_: lower limit and d_U_: upper limit of 95% one-sided CI). Both were compared with the pre-defined clinically accepted ranges for the corresponding parameters (± 0.5 g/dl for Hb and ± 45 IU/kg/week for EPO dosage, based on the respective reference means). The intervals were calculated by means of analysis of covariance model including treatment group as a factor and study centre and baseline values as covariates.

Secondary endpoints were summaries of categorical variables and included frequency and percentage of patients at each level of response. Treatment difference was analysed using two sample t-test or Wilcoxon rank-sum test for continuous and, Chi-square (χ^2^) test or Fisher’s exact test for categorical parameters.

Treatment emergent adverse events (TEAE) during the maintenance phase were based on number, percentage and the corresponding 95% CI of patients experiencing TEAEs in each treatment group. The significance for treatment difference was done using χ^2^-test or Fisher’s exact test. All other secondary endpoints used descriptive statistics to represent changes from baseline at each visit.

## Results

### Study population

**Fig. 2:**
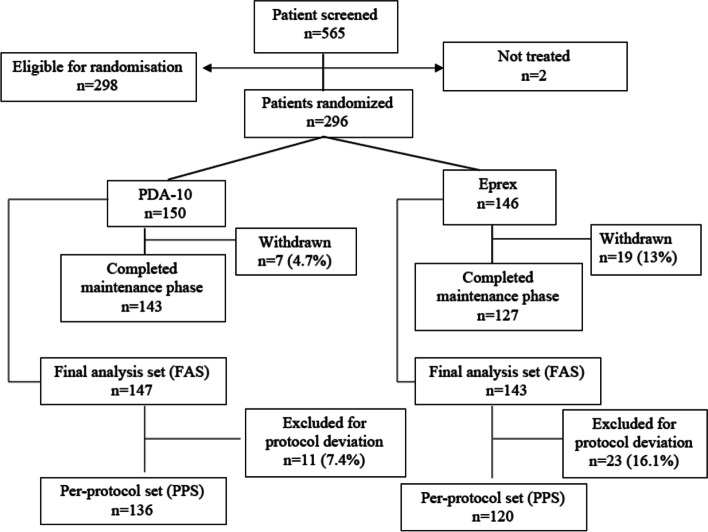
The study flow of subjects from screening up to the per-protocol set

Of the total 565 patients screened between October 2013 till June 2016, 298 were eligible for randomisation of which two patients, one from each group, were not treated (one at patient’s request in the PDA10 group and the other lost to follow-up in the Eprex® group). The PDA10 group had 150 subjects whilst the Eprex® group had 146 subjects who were eventually treated. Maintenance phase was completed by 143 patients in the PDA10 and 127 in the Eprex® groups. A total of 7 (2 due to AEs) and 19 (11 due to AEs) patients were withdrawn during the maintenance phase from the PDA10 and Eprex® groups, respectively. Of the 296 patients randomised and started on the study medications, 290 (147 and 143 from the PDA10 and Eprex® groups, respectively) were included in the FAS. Excluding those who had been withdrawn and had major protocol deviations from the FAS, total 256 subjects (136 in PDA10 group, 120 in Eprex® group) were included in the PPS (Fig. 2). Total number analysed for safety was 296.

Demographic data and baseline characteristics of the PPS are presented by treatment group in Table 1.

**Table 1:** Demographics and baseline characteristics of the per-protocol set

	PDA-10 (*N* = 136)	Eprex® (*N* = 120)	*P* value
Mean age (range), years	54.09 (11.26)	51.01 (14.04)	NS
Sex [n (%)]			
Male	85.00 (62.50)	76.00 (63.33)	NS
Female	51.00 (37.50)	44.00 (36.67)	
Race/Ethnicity [n (%)]			
Korean	35.00 (25.74)	27.00 (22.50)	NS
Malaysian	101.00 (74.26)	93.00 (77.50)	
Mean weight (kg)	64.48 (14.02)	63.49 (12.66)	
Mean BMI (kg/m2)	24.86 (4.58)	24.34 (4.38)	
Mean duration of renal anaemia (range) (month)	64.46 (56.18)	57.58 (49.67)	NS
Mean duration of ESKD (range) (month)	66.01(59.84)	60.16 (52.34)	
Mean baseline Hb (range) (g/dl)	11.01 (0.58)	11.00(0.59)	
Mean baseline dosage of epoetin (range) (IU/week)	107.52 (69.18)	114.58 (73.86)	
Mean baseline ferritin (range) (ng/ml)	692.39 (686.85)	740.18 (602.42)	
Mean baseline transferrin saturation (range) (%)	34.49(20.61)	31.24 (13.49)	

*BMI* body mass index, *ESKD* end stage kidney disease, *Hb* haemoglobin, *IU* international unit

The two treatment arms were similar in demographics and baseline characteristics. The mean age was 52.64 years (standard deviation [SD] 12.71) and majority were males (62.89%). Up to 75.8% of study subjects were from Malaysia and 24.2%, from Korea. Mean duration of renal anaemia was 61.23 months.

There were no statistically significant differences between treatment groups in the proportion of patients with a past medical history and those with concurrent diseases. The most common concurrent disease by preferred term (PT) was ESKD (100%) followed by renal anaemia (96.06%) and hypertension (91.80%). All patients in both groups had history of taking anti-anaemic preparations whilst 96.09% were also on vitamins. The inter-group difference in the proportion of patients with concomitant medications was also not significant.

### Primary efficacy endpoints

The mean change in Hb level between the baseline and evaluation periods in the PPS is presented in Table 2. Both the PPS and FAS had no significant difference in baseline Hb levels. The results in the FAS showed a similar trend to those in the PPS i.e. the least square mean (± standard error) for the mean absolute change in Hb level during the evaluation period compared to the baseline period was − 0.283 (± 0.084) g/dl and − 0.275 (± 0.085) g/dl in the PDA10 and Eprex® groups, respectively. Both sets had significant difference in mean Hb change between treatment groups showing therapeutic equivalence (two one sided test result, *p* < 0.0001).

**Table 2:** Mean change in Hb level from baseline to evaluation periods in the PPS

	PDA-10 (*n* = 136)	Eprex® (*n* = 120)
Mean baseline Hb (SD) (g/dl)	11.011 (0.577)	11.001 (0.587)
Mean evaluation Hb (SD) (g/dl)	10.835 (0.811)	10.883 (0.985)
Mean absolute Hb change (SD) (g/dl)	−0.176 (0.914)	−0.118 (1.114)
Least Square mean difference of “PDA10 – Eprex®”		
LS mean	−0.259 (0.088)	−0.194 (0.089)
LS mean for difference	−0.066 (0.111)	
95% one-sided lower limit (≥ − 0.5) (*p* value)	−0.249 (<.0001)	
95% one-sided upper limit (≤ + 0.5) (*p* value)	0.117 (<.0001)	

*SD* standard deviation, *LS* least square, *Hb* haemoglobin

The weekly dose/kg body weight during the baseline and evaluation periods and the change in weekly dose during the evaluation period from the baseline period in the PPS are presented in Table 3. In the FAS analysis, the least square mean (± standard error) for the mean absolute change in the weekly dose/kg body weight was 7.77 (± 4.21) IU/kg/week and − 7.31 (± 4.25) IU/kg/week in the PDA10 group and Eprex® groups, respectively. The results in the PPS and FAS showed therapeutic equivalence between the test and reference drugs (two one-sided test result, *p* < 0.0001).

**Table 3:** Mean change in weekly dose/kg body weight between the baseline and evaluation periods in the PPS

	PDA-10 (*n* = 136)	Eprex® (*n* = 120)
Mean baseline epoetin dosage (IU/kg/week)	107.52 (69.18)	114.58 (73.86)
Mean evaluation epoetin dosage (IU/kg/week)	117.53 (81.35)	104.28 (65.87)
Mean absolute change in epoetin dose (IU/kg/week)	10.01 (44.64)	−10.30 (56.09)
Least Square mean difference of “PDA10 – Eprex®”		
LS mean	9.55 (4.81)	−8.71 (4.82)
LS mean for difference	18.26 (6.02)	
95% one-sided lower limit (≥ − 0.5) (*p* value)	8.31 (< 0.0001)	
95% one-sided upper limit (≤ + 0.5) (*p* value)	28.21 (< 0.0001)	

*IU* international unit, *LS* least square

### Secondary efficacy endpoints

**Fig. 3:**
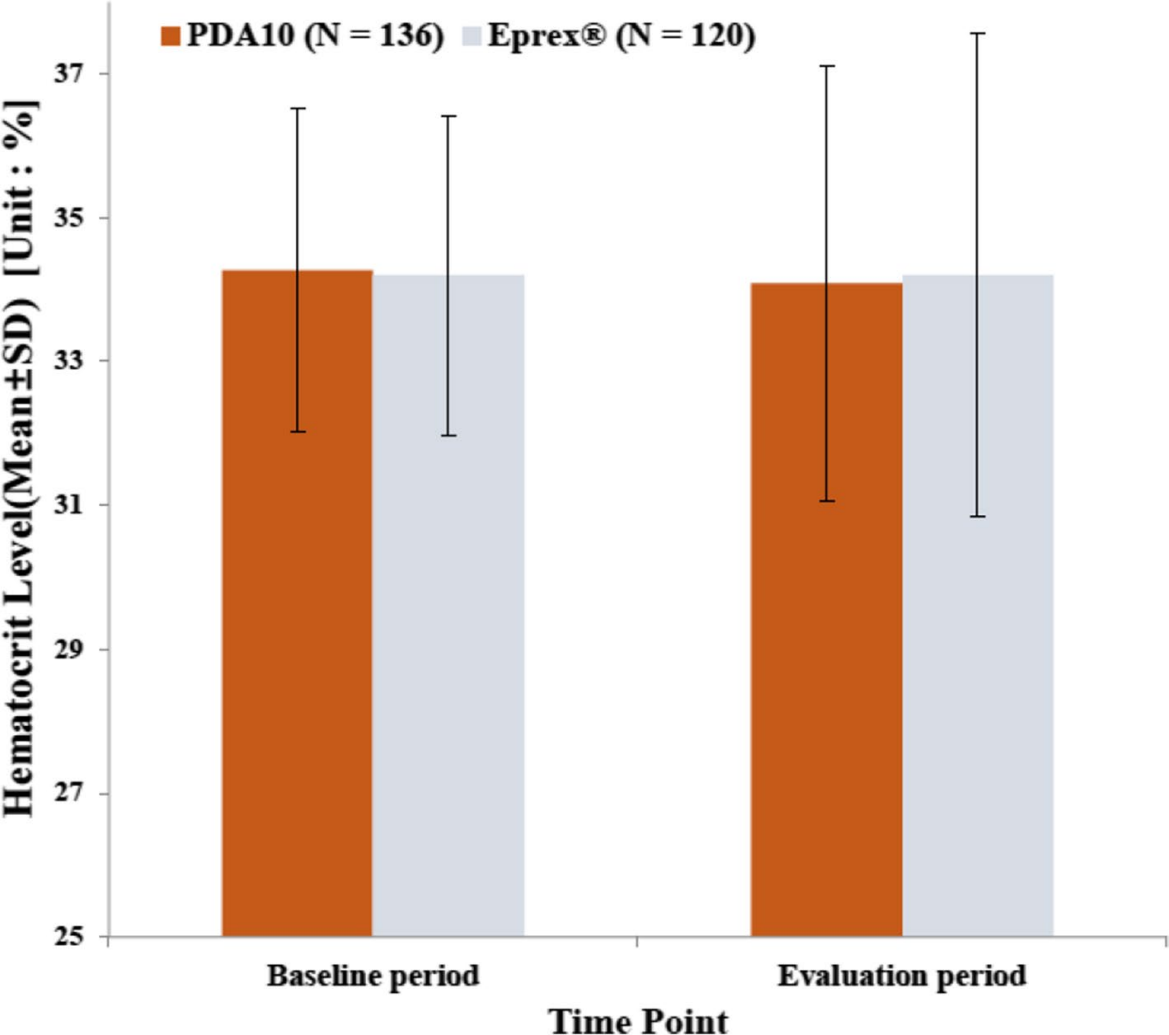
Mean change in haematocrit levels between the baseline and evaluation periods in the PPS. The mean haematocrit levels (in percentage) in subjects of the per-protocol set (PPS) in the PDA-10 and Eprex® arms at baseline and evaluation periods. The error bars represent the standard deviation (SD)

The haematocrit levels during the baseline and evaluation periods and their changes are presented in Fig. 3. The mean (± SD) change at the evaluation period compared to the baseline was − 0.71% (± 2.944) in the PDA10 group (*p* = 0.552) and 0.007% (± 3.498) in the Eprex® group (*p* = 0.608) with no statistical significance between both groups (*p* = 0.445). The FAS showed trends similar to those of the PPS.

**Fig. 4:**
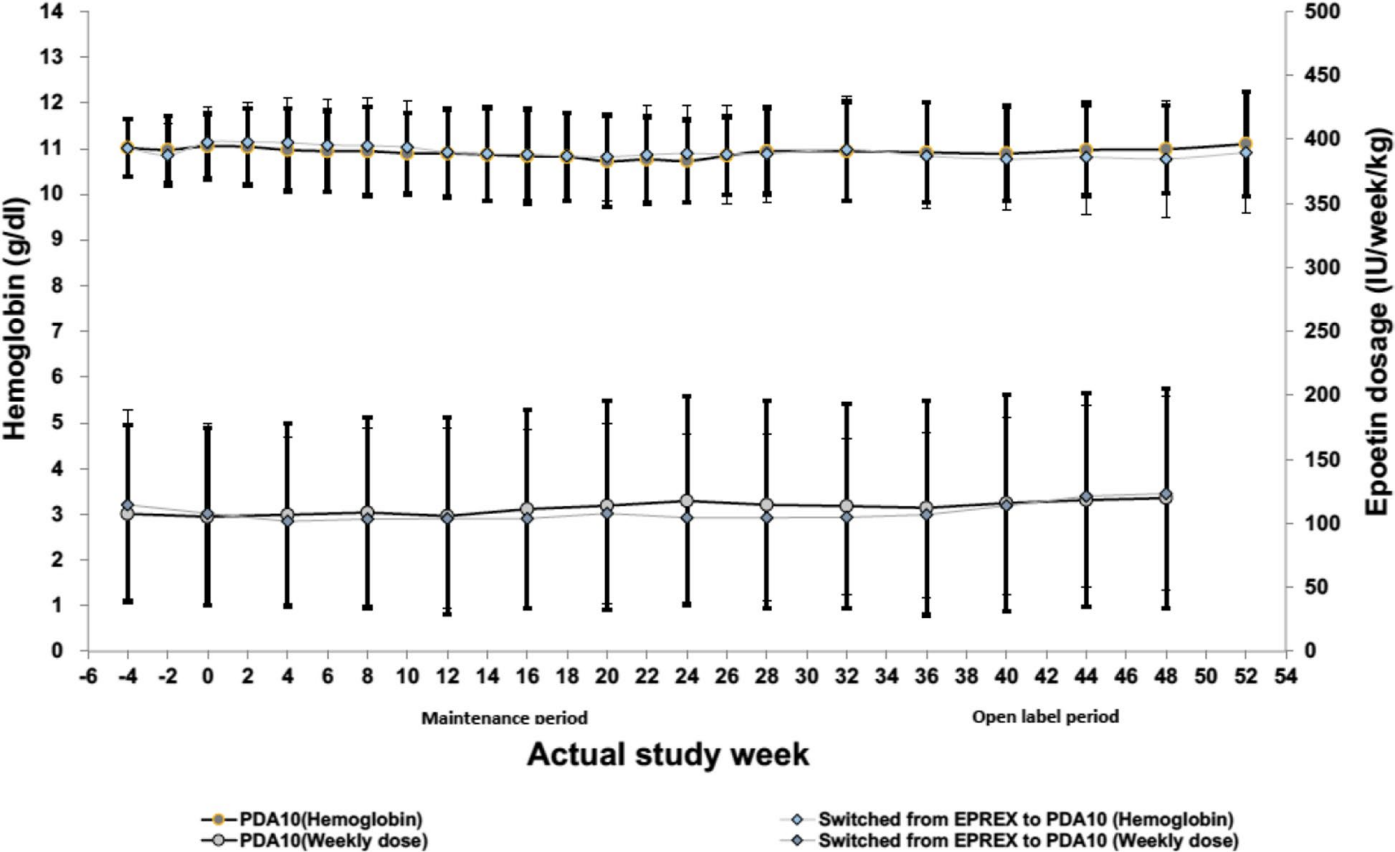
Hemoglobin levels (mean ± SD) and epoetin dosage during the study period The line graphs depict the average haemoglobin levels (top) and the epoetin dosage (bottom) of subjects during the maintenance and open label phases. The error bars refer to the standard deviation (SD)

One hundred and eleven (81.62%) and 108 (90.0%) patients in the PDA10 and Eprex® groups, respectively had a Hb level out of the target range (10–12 g/dl) during the maintenance phase and the difference in the proportion between the treatments in both PPS and FAS was not statistically significant (*p* = 0.057). In the PPS, 85 (62.50%) and 61 (50.83%) of patients in the PDA10 and Eprex® groups, respectively were within target range during the evaluation period with no difference between both groups (*p* = 0.0600). However, the proportion of patients within the target range showed statistical difference between the treatments in the FAS (*p* = 0.036). As shown in Fig. 4, the mean Hb concentrations and ESA dosage were stable throughout the treatment period in both treatment arms.

Except for one patient in the PPS PDA10 group, all subjects showed a change in the dose/kg body weight (dry body weight) during the maintenance phase, i.e. One hundred thirty-five patients [99.26%, 491 events] and 120 patients [100%, 470 events] in the PDA 10 and Eprex® groups, respectively. None in the PDA10 group and 1 patient (0.83%, 5 events) in the Eprex® group received blood transfusion (difference between treatments, *p* = 0.469). The results for both these parameters in the FAS were similar to the PPS.

### Immunogenicity assessment

None of the patients assessed at baseline were positive for immunogenicity. At week 28, anti-epoetin antibodies were not observed in both treatment groups. The immunogenicity test results of both treatment groups were comparable.

### Safety assessment

A total of 296 patients (150 and 146 in the PDA10 and Eprex® groups, respectively) who met the definition of the safety set were included (Table 4).

**Table 4:** Summary of TEAEs in the safety set

	PDA10 (***N*** = 150)	Eprex® (***N*** = 146)	***P***-value
**Subjects with TEAEs**	98(65.33) [273]	88(60.27) [231]	0.3678 (c)
**Infection**	34(22.67) [49]	35(23.97) [51]	0.7905 (c)
**Infection with corresponding category**	29(19.33) [35]	28(19.18) [39]	0.9730 (c)
a. Respiratory tract infections	21(14.00) [26]	24(16.44) [33]	0.5591 (c)
b. Gastrointestinal infections	6(4.00) [6]	4(2.74) [4]	0.7499 (f)
c. Urinary tract infections	0	0	–
d. Vascular access infections	3(2.00) [3]	2(1.37) [2]	1.0000 (f)
No corresponding category	10(6.67) [14]	11(7.53) [12]	0.7713 (c)
**Non-Infection**	89(59.33) [224]	78(53.42) [180]	0.3054 (c)
**Non-Infection with corresponding category**	39(26.00) [60]	35(23.97) [46]	0.6871 (c)
a. Poorly controlled BP	26(17.33) [41]	20(13.70) [26]	0.3882 (c)
b. Ischemic coronary artery disease	3(2.00) [3]	3(2.05) [3]	1.0000 (f)
c. Heart failure	13(8.67) [16]	8(5.48) [8]	0.2856 (c)
d. Ischemic stroke	0	0	–
e. Haemorrhagic stroke	0	0	–
f. AVF thrombosis	0	7(4.79) [7]	0.0066 (f)
g. Death	0	2(1.37) [2]	0.2424 (f)
No corresponding category	76(50.67) [164]	61(41.78) [134]	0.1253 (c)

*TEAEs* treatment-emergent adverse events

*P*-value: Difference between the treatment groups (Chi-square test (c) or Fishers exact test (f))

Note: Denominator of percentage is the number of subjects in the column

TEAEs are displayed as number of subjects (percentage of subjects) [number of events]

The mean duration of the study drug administration by treatment group was 187.3 days and 176.3 days in the PDA10 and Eprex® groups. There was no difference in extent of exposure between both groups.

There was no difference in the number of subjects experiencing TEAE in both groups. The most common infection related TEAE was respiratory tract infections in 14.00 and 16.44% followed by gastrointestinal infections in 4.00 and 2.74% of patients in the PDA-10 and Eprex® groups, respectively. This was statistically not significant. The most common adverse drug reactions (ADRs) was “Hypertension” in 0.67% (PDA10 group) and 1.37% (Eprex® group). Other than one patient each reporting “blood pressure inadequately controlled” and “rash” in the PDA10 group, there were no other ADRs reported. In this study, all clinically significant abnormal laboratory findings were reported as AEs and were found to be unrelated to the study products. There were also no deaths or serious AEs reported in both groups that were related to the study products.

## Discussion

In this 28-week randomised controlled study, PDA10 was shown to be therapeutically equivalent to the originator Eprex® in terms of absolute change in Hb levels and comparable safety profile in treating renal anaemia for patients undergoing maintenance HD.

The target Hb range of 10–12 g/dl was chosen based on the KDIGO guidelines and adapted to local medical practice. The ESA dosage titration scheme was designed based on previous similar studies [20, 21]. Determination of therapeutic equivalence was done in accordance with the standards set by the ICH Statistical Principles for Clinical Trials document. The results were based on both the FAS and PPS, wherein, the PPS is considered the most conservative approach for this study type [20].

Primary efficacy endpoints in this study focused on mean change in Hb level (*d*). In this study, the difference in mean change of Hb levels from baseline were both significantly different when compared with the *dL* and *dU* limits (*dL ≤ d ≤ dU*). The difference in both groups were within the pre-determined lower (− 0.5) and upper (0.5) limits showing therapeutic equivalence between both drugs.

Conversion from Eprex® to PDA10 did not affect the Hb stability and consistent with other published literature [20–24], did not require higher epoetin dosage in the PDA10 arm. In contrary, a retrospective study [25] reported that switching to biosimilars from ESA originators required 40% higher doses to maintain Hb stability. Our study highlighted the potential cost saving of PDA10 as compared to the originator in managing renal anaemia among HD patients. A high proportion of our subjects in both groups had Hb level out of target range during the maintenance phase. These Hb fluctuations are not uncommon and have been well described in the use of ESA in renal anaemia. However, with the appropriate epoetin dosage adjustment, these fluctuations did not increase occurrence of AEs secondary to high Hb level such as worsening hypertension, stroke and vascular access thrombosis.

There were no significant differences in the safety profile in both groups. Though the incidence of TEAEs leading to death was higher in the Eprex® group, all TEAEs leading to death were considered unrelated to the products. There were no cases of pure red cell aplasia reported in both groups and no signals for immunogenicity. This is consistent with other reports on biosimilar epoetin [20–22].

## Conclusion

This study has confirmed the efficacy, safety profile and therapeutic equivalence of PDA10 as compared to the originator epoetin-α, Eprex®. The dosage requirement for PDA10 to maintain Hb stability is similar to Eprex®. This biosimilar epoetin-α may offer a more cost-effective erythropoietin therapy and improve the access of ESA therapy for patients undergoing haemodialysis treatment.

## Supplementary Information

**Additional file 1: Table 5.** TEAEs with no corresponding category.

## Data Availability

The datasets used and/or analysed during the current study are available from the corresponding author on reasonable request.

## Abbreviations

ADRs: Adverse drug reactions
AEs: Adverse events
χ^2^: Chi-square test
CHO-DG44: Chinese Hamster Ovary cells
CKD: Chronic kidney disease
CI: Confidence interval
ESKD: End stage kidney disease
PDA-10: Epoetin-Alfa
ESA: Erythropoietin stimulating agent
e-GFR: Estimated glomerular filtration rate
FAS: Full analysis set
HD: Haemodialysis
Hb: Haemoglobin
IV: Intravenous
kg: Kilogram
PPS: Per protocol set
PFS: Pre-filled syringes
PT: Preferred term
rhEPO: Recombinant human erythropoietin
SD: Standard deviation
SOC: Standard of care
TSAT: Transferrin saturation
TEAE: Treatment emergent adverse events
TOST: Two one-sided tests

## References

[1] Fishbane S, Spinowitz B (2018). Update on Anemia in ESRD and earlier stages of CKD: Core curriculum 2018. Am J Kidney Dis.

[2] Ryu S-R, Park SK, Jung JY (2017). The prevalence and Management of Anemia in chronic kidney disease patients: result from the KoreaN cohort study for outcomes in patients with chronic kidney disease (KNOW-CKD). J Korean Med Sci.

[3] Stauffer ME, Fan T (2014). Prevalence of anemia in chronic kidney disease in the United States. PLoS One.

[4] Idris I, Tohid H, Muhammad NA (2018). Anaemia among primary care patients with type 2 diabetes mellitus (T2DM) and chronic kidney disease (CKD): a multicentred cross-sectional study. BMJ Open.

[5] El-Achkar TM, Ohmit SE, McCullough PA (2005). Higher prevalence of anemia with diabetes mellitus in moderate kidney insufficiency: the kidney early evaluation program. Kidney Int.

[6] Eschbach JW, Adamson JW (1988). Recombinant human erythropoietin: implications for nephrology. Am J Kidney Dis.

[7] Valderrábano F (1996). Erythropoietin in chronic renal failure. Kidney Int.

[8] Winearls CG (1995). Historical review on the use of recombinant human erythropoietin in chronic renal failure. Nephrol Dial Transplant.

[9] Eschbach JW, Abdulhadi MH, Browne JK (1989). Recombinant human erythropoietin in anemic patients with end-stage renal disease. Results of a phase III multicenter clinical trial. Ann Intern Med.

[10] Casati S, Passerini P, Campise MR (1987). Benefits and risks of protracted treatment with human recombinant erythropoietin in patients having haemodialysis. Br Med J (Clin Res Ed).

[11] Eschbach JW, Downing MR, Egrie JC, Browne JK, Adamson JW (1989). USA multicenter clinical trial with recombinant human erythropoietin (Amgen). Results in hemodialysis patients. Contrib Nephrol.

[12] Winearls CG, Oliver DO, Pippard MJ, Reid C, Downing MR, Cotes PM (1986). Effect of human erythropoietin derived from recombinant DNA on the anaemia of patients maintained by chronic haemodialysis. Lancet..

[13] KDIGO 2012 clinical practice guideline for Anaemia in chronic kidney disease. Kidney Int Suppl. 2012;4(2):279–331.

[14] Malaysian Society of Nephrology. 24th report of the Malaysian dialysis and transplant registry 2016. https://www.kisupplements.org/issue/S2157-1716(12)X7400-8.

[15] United States Renal Data System. US Renal Data System 2019 Annual Data Report: Epidemiology of Kidney Disease in the United States: Executive Summary.

[16] Drüeke TB, Locatelli F, Clyne N (2006). Normalization of hemoglobin level in patients with chronic kidney disease and anemia. N Engl J Med.

[17] Pfeffer MA, Burdmann EA, Chen C-Y (2009). A trial of Darbepoetin Alfa in type 2 diabetes and chronic kidney disease. N Engl J Med.

[18] Singh AK, Szczech L, Tang KL (2006). Correction of anemia with epoetin alfa in chronic kidney disease. N Engl J Med.

[19] Oh MK, Yoon J, Cho D-Y (2015). Pharmacokinetic and pharmacodynamic comparison of two recombinant human erythropoietin formulations, PDA10 and Eprex, in healthy Korean male volunteers: a randomised, double-blinded, single-dose, two-period crossover study. Clin Drug Investig..

[20] Haag-Weber M, Vetter A, Thyroff-Friesinger U (2009). Therapeutic equivalence, long-term efficacy and safety of HX575 in the treatment of anemia in chronic renal failure patients receiving hemodialysis. Clin Nephrol.

[21] Hörl WH, Locatelli F, Haag-Weber M, Ode M, Roth K (2012). Prospective multicenter study of HX575 (biosimilar epoetin-α) in patients with chronic kidney disease applying a target hemoglobin of 10--12 g/dl. Clin Nephrol.

[22] Haag-Weber M, Eckardt KU, Hörl WH, Roger SD, Vetter A, Roth K (2012). Safety, immunogenicity and efficacy of subcutaneous biosimilar epoetin-α (HX575) in non-dialysis patients with renal anemia: a multi-center, randomized, double-blind study. Clin Nephrol.

[23] Hörbrand F, Bramlage P, Fischaleck J, Hasford J, Brunkhorst R (2013). A population-based study comparing biosimilar versus originator erythropoiesis-stimulating agent consumption in 6,117 patients with renal anaemia. Eur J Clin Pharmacol.

[24] Thadhani R, Guilatco R, Hymes J, Maddux FW, Ahuja A (2018). Switching from Epoetin Alfa (Epogen®) to Epoetin Alfa-Epbx (RetacritTM) using a specified dosing algorithm: a randomized, non-inferiority study in adults on hemodialysis. Am J Nephrol.

[25] Minutolo R, Bolasco P, Chiodini P (2017). Effectiveness of switch to erythropoiesis-stimulating agent (ESA) Biosimilars versus maintenance of ESA originators in the real-life setting: matched-control study in hemodialysis patients. Clin Drug Investig.

