# Correction to: A multicentre, multi-national, double-blind, randomised, active-controlled, parallel-group clinical study to assess the safety and efficacy of PDA10 (Epoetin-Alfa) vs. Eprex® in patients with anaemia of chronic renal failure

**DOI:** 10.1186/s12882-021-02651-0

**Published:** 2022-01-03

**Authors:** Soo Kun Lim, Bak Leong Goh, Ravindran Visvanathan, Su Hyun Kim, Jin Seok Jeon, Sung Gyun Kim, Jae Hyun Chang, Chun Soo Lim, Zaki Morad

**Affiliations:** 1 Renal Division, Department of Medicine, Faculty of Medicine, University of Malaya, 59100 Kuala Lumpur, Malaysia; 2 Serdang Hospital, Kajang, Malaysia; 3 Prince Court Medical Centre, Kuala Lumpur, Malaysia; 4 Chung-Ang University Hospital, Seoul, South Korea; 5 Soonchunhyang University Hospital, Seoul, South Korea; 6 Hallym University Sacred Heart Hospital, Anyang, South Korea; 7 Gil Medical Centre, Gachon University College of Medicine, Incheon, South Korea; 8 Seoul National University Boramae Medical Centre, Seoul, South Korea; 9 KPJ Ampang Puteri Specialist Hospital, Ampang, Malaysia

**Correction to: BMC Nephrology 22, 391 (2021)**

**https://doi.org/10.1186/s12882-021-02601-w**

Following publication of the original article [1], the authors would like to correct a spelling mistake in title and in the body of the text.

The word currently reads: Alpha

The word should read: Alfa

The title has been updated above and the original article [1] has been corrected.
