# Supplementary material for: A multicentre, multi-national, double-blind, randomised, active-controlled, parallel-group clinical study to assess the safety and efficacy of PDA10 (Epoetin-Alfa) vs. Eprex® in patients with anaemia of chronic renal failure
Source: BMC Nephrol. 2021 Nov 25;22:391. doi: 10.1186/s12882-021-02601-w (PMC8620966; doi:10.1186/s12882-021-02601-w)
Supplement: Supplementary file 1 — Additional file 1: Table 5. TEAEs with no corresponding category. [file 12882_2021_2601_MOESM1_ESM.pdf]

1. TITLE PAGE

PANGEN BIOTECH

A MULTI-CENTER, MULTI-NATIONAL, DOUBLE-BLIND, RANDOMIZED, ACTIVE-CONTROLLED, PARALLEL-GROUP CLINICAL STUDY TO ASSESS SAFETY AND EFFICACY OF PDA10 (EPOETIN-ALFA) COMPARED TO EPREX® IN PATIENTS WITH ANEMIA OF CHRONIC RENAL FAILURE

Protocol PG-EPO-Ph3

Confidentiality Statement

This document is the property of PanGen Biotech and may not—in full or part—be passed on, reproduced, published, distributed to any person, or submitted to any regulatory authority without the express written permission of PanGen Biotech.

Protocol Agreement

I agree:

- *To conduct the study in accordance with ICH-GCP, the declaration of Helsinki, and local ethical and legal requirements*
- *To conduct the study in compliance with this protocol, any future amendments and not to implement any changes to the protocol without agreement from the Sponsor and prior review and written approval from the Institutional Review Board (IRB).*
- *To ensure that all persons assisting me with the study are adequately informed about the Investigational Product(s) and of their study-related duties and functions as described in the protocol*

Principal Investigator

Name:

Signature	*Date*

Protocol Agreement

I agree:

- *To conduct the study in accordance with ICH-GCP, the declaration of Helsinki, and local ethical and legal requirements*
- *To conduct the study in compliance with this protocol, any future amendments and not to implement any changes to the protocol without agreement from the Sponsor and prior review and written approval from the Institutional Review Board (IRB).*
- *To ensure that all persons assisting me with the study are adequately informed about the Investigational Product(s) and of their study-related duties and functions as described in the protocol*

Representative of Sponsor

Name:

________________________________ ________________________
Signature *Date*

Note:

This study is funded by a grant from CCM Duopharma Biotech Bhd. and PanGen Biotech Inc.

2. SYNOPSIS

CLINICAL STUDY SYNOPSIS: Study PG-EPO-Ph3	
**Study Number**	PG-EPO-Ph3
**Study title**	A multi-center, multi-national, double-blind, randomized, active-controlled, parallel-group clinical study to assess safety and efficacy of PDA10 (epoetin-alfa) compared to Eprex® in patients with anemia of chronic renal failure
**Development Phase**	III
**Objective**	• The primary objective is to demonstrate the therapeutic equivalence of PDA10 to Eprex® in patients with end stage renal failure (ESRD) on chronic hemodialysis.<br>The therapeutic equivalence shall be evaluated by the difference between treatment groups in the change in hemoglobin from baseline, while taking into account differences in epoetin dosage.<br>• The secondary objective is to assess the safety and tolerability of PDA10.<br>• The aim of the open label extension phase is to assess the long-term safety and tolerability of PDA10.
**Study Design**	• A multi-center, multi-national, randomized, double-blind, active-controlled, parallel-group clinical trial<br>• Study period: 24 months after IRB approval<br>• Study period per patient: approximately 64 weeks<br>- Titration phase: 12 weeks (in substantiated cases, a prolongation of the titration phase will be allowed up to 20 weeks and the baseline period [observation period] is the last four weeks of the titration phase.)<br>- Maintenance phase: 28 weeks including 4 weeks of the evaluation period (the evaluation period is the last 4 weeks of the maintenance phase.)<br>- Open-label extension phase: 24 weeks
**Number of Patients**	The planned sample size is 126 per group, which will be expected to give an overall 80% power for the proof of equivalence. The total number of patients to be enrolled will be 316, including an assumed drop-out/protocol violation rate of 20%.
**Inclusion/Exclusion criteria**	**Inclusion Criteria**<br>To be eligible to participate in the study, patients must meet the following criteria:<br>1. Anemic patients with end stage renal failure (ESRD) on chronic hemodialysis<br>2. Patients must be at least 18 years old but less than 75 years old at Screening Visit<br>3. Patients on hemodialysis through a functioning native arterio-venous fistula<br>4. Patients must be at their dry body weight or within 5% of it during the baseline period (observation period)<br>5. Patients must be able to understand the information provided to them and to give written Informed Consent

CLINICAL STUDY SYNOPSIS: Study PG-EPO-Ph3	
	6. Female patients of childbearing potential must have a negative serum pregnancy test at the Screening Visit and Baseline Visit. Females must be surgically sterile, postmenopausal for at least 1 year prior to Screening Visit or must be using an acceptable method of birth control ([oral, non-oral or implantable] hormone contraceptives, intrauterine contraceptive device or blockers and spermicides) effectively. Abstinence is not an acceptable method of contraception for the study.<br>7. Patients must have the following at Screening Visit or prior to randomization as well as at the baseline period (or observation period)<br>• Patients on erythropoietin treatment prior to Screening<br>Note: If patients who have been on Eprex® treatment before this study have hemoglobin level less than 10 g/dl during the baseline period (observation period), they must participate in the titration phase.<br>• Hemoglobin level with 10 - 12 g/dl and a stable IV dose of Eprex® without transfusion prior to randomization (A stable IV dose is defined as not more than 25% change up or down in weekly dose and no clinically relevant change of regimen in frequency of haemodialysis for the baseline period [observation period])<br>• Patients on Eprex® treatment for at least 12 weeks; patients on stable and adequate hemodialysis at least 3 times a week and with a documented URR > 65% or delivered KT/V ≥ 1.2 in the past 6 months prior to randomization (If patients meet this criteria, they will be randomized without participating in the titration phase)<br>• Serum ferritin level at least 100 ng/ml and/or transferrin saturation (TSAT) at least 20% prior to randomization<br><br>**Exclusion Criteria**<br>Patients who meet any of the following criteria will not be eligible to participate in the study**:**<br>1. Patients with a temporary or permanent catheters or synthetic grafts<br>2. Patients with uncontrolled hypertension, defined as a pre-dialysis diastolic BP of greater than 110 within 12 weeks prior to randomization<br>3. Patients with severe diseases within the last 6 months prior to randomization (e.g. stroke, transient ischemic attack, myocardial infarction, cerebrovascular accident, coronary artery bypass graft, deep venous thrombosis, unstable angina, decompensate congestive heart failure (New York Heart Association [NYHA] class III~IV), or other thromboembolic events)<br>4. Surgery patients who for any reason cannot receive adequate antithrombotic prophylaxis or treatment<br>5. Patients with a current or recent known history of a severe hyperparathyroidism or (PTH > 1500 pg/ml within 12 weeks prior to randomization.)<br>6. Patients with hyperkalemia<br>7. Patients with epilepsy<br>8. Patients with malnutrition (serum albumin < 3.5g/dl prior to randomization)<br>9. Patients with an acute infection, acute hepatitis (including A, B, C

CLINICAL STUDY SYNOPSIS: Study PG-EPO-Ph3	
	type) or chronic hepatitis B or C requiring treatment , or HIV infection<br>10. Patients with significant inflammation (CRP >30 mg/L within 12 weeks prior to randomization)<br>11. Patients with a history of gastrointestinal bleeding within the last 6 months before Screening<br>12. Patients with any active, uncontrolled systemic or inflammatory disease that in the Investigator's opinion may be significant to exclude participation in the study<br>13. Patients of need for blood transfusions within 12 weeks prior to randomization<br>14. Patients with history of pure red cell aplasia (PRCA) or anti-erythropoietin antibodies<br>15. Patients with a history of malignancy of any organ system within the last 5 years prior to Screening<br>16. Patients with a current diagnosis of anemia due to folic acid and/or Vitamin B12 deficiencies, hemolysis, or gastrointestinal bleeding or a history of active blood or bleeding disorders within the last 6 months before Screening<br>17. Patients who have received immunosuppressive treatment or use of other medication known to influence erythropoiesis 12 weeks prior to randomization<br>18. Patients with hypersensitivity to the active substance or to any of the excipients<br>19. Patients who have been treated with any other investigational drug within 4 weeks prior to Screening<br>20. Patients who currently are pregnant or lactating<br>21. Patients who are not cooperative or not able to follow the clinical study procedures<br>22. Patients who are judged to be ineligible to the clinical study at the Investigator’s discretion for other reasons such as alcohol and drug abuse
**Test Product and Mode of Administration**	**PDA10 (epoetin alfa; biosimilar)**<br>• Test product: PDA10, Pre-filled syringe (PFS)<br>• Mode of administration: IV, one to three times per week
**Reference Product and Mode of Administration**	**Eprex® (epoetin alfa; original)**<br>• Reference product: Eprex®, Pre-filled syringe (PFS)<br>• Mode of administration: IV, one to three times per week
**Dosage**	**<u>Observation period</u>**<br>In order to evaluate the Hb level of patients for screening, patients will be monitored on the Eprex® dosage previously treated and Hb levels for four weeks from screening visit and the Hb levels will be evaluated every 2 weeks. When the Hb level and iron status meet the followed criteria, the patients will proceed to the maintenance phase or the titration phase.<br>• <u>Criteria to proceed the maintenance phase without titration phase</u><br>Patients on Eprex® treatment for at least 12 weeks and with hemoglobin within the target range of 10.0 to 12.0 g/dl and a stable IV dose of Eprex®, and adequate iron store (serum ferritin level ≥ 100 ng/mL and/or transferrin

CLINICAL STUDY SYNOPSIS: Study PG-EPO-Ph3	
	saturation ≥ 20%) in observation period will proceed to enter the maintenance phase. Hb level measured during this observation period will be considered as baseline level.<br><br>• Criteria to proceed the titration phase<br>① Patients treated with other EPO products for at least 12 weeks<br>If the patients are treated with other EPO products, the patients should proceed to the titration phase to exposure the Eprex® for at least 12 weeks.<br>② Hb is below 10 g/dl even if the patients were treated with Eprex® for at least 12 weeks; in the event, a patients' Hb is to decline below 10 g/dl during the observation period, patients should switch to titration phase.<br><br>**Titration phase**<br>The initial dosage of Eprex® will be the same with the erythropoietin dosage initial dosage if Hb level is less than 10 g/dL prior to Screening Visit. The Hb level of patients is monitored every 2 weeks during titration phase. Eprex® dose changes will be made every four weeks on the basis of the most current Hb level—more frequent changes will be made only when the Hb value or the rate of Hb change is outside the preset safety as follows;<br><br>• If Hb level increase in the past two weeks is > 1.0 g/dl or Hb level is approaching 12 g/dl, the dose will be decreased by 25% of current dosage. If the hemoglobin continues to increase or exceed 12 g/dl, the treatment should be temporarily withheld until the hemoglobin begins to decrease, at which point therapy should be reinitiated at a dose approximately 25% below the previous dose. If dose reduction is needed, the amount given per dose or the number of weekly injections will be reduced, or both will be reduced.<br>• If Hb level increase in the past two weeks is < 0.3 g/dl and Hb level is below 10 g/dl, the dose will be increased by 25% of current dosage.<br>• If Hb increase in the past 2 weeks is ≤ 1.0 g/dl and Hb level is in the target range (10 – 12 g/dl), the dose will be maintained.<br><br>When a change in Eprex® dose is indicated, a one-step change (25% of current dosage) is performed based on the stepwise dosing schedule refer to appendix III.<br><br>* The patients will be treated with iron therapy based on iron level as follows;<br>① Marginal iron store (all patients will have serum ferritin level > 100 to be eligible for entry) - Eprex® dose increasing during the titration phase and IV iron as appropriate. For patients unable to access IV iron therapy, Sponsor will support the iron therapy.<br>② Hb is below 10 g/dl and serum ferritin level > 500 - Eprex® dose increasing during the titration phase and a close iron monitoring. If serum ferritin is to decline rapidly, giving IV iron should be considered

<table>
<tr><th colspan="2">CLINICAL STUDY SYNOPSIS: Study PG-EPO-Ph3</th></tr>
<tr><td></td><td>appropriately. For patients unable to access IV iron therapy, Sponsor will support the iron therapy.

During last four weeks of the titration period, the Hb level will be evaluated to meet the inclusion criteria to conduct the randomization similar to observation period. Patients with stable Hb maintained between 10 and 12 g/dl and on stable Eprex® dose will proceed to enter the maintenance phase at the end of the titration period. The last four weeks of the titration phase will be considered the baseline period, and Hb levels measured during baseline period will be considered as baseline level.

<u>Maintenance Phase</u>
The IV dose has to be adjusted individually to maintain hemoglobin between 10 - 12 g/dl. The hemoglobin, hematocrit, and dose will be monitored every two weeks during the maintenance phase. Investigators will decide to change or maintain the dosage of Eprex® or PDA10 every four weeks based on Hb level. The dosage will be determined according to the dose adjustment scheme below, and if hemoglobin level is not increased by more than 1 g/dl in any 2-week period or is not approaching 12 g/dl or below 10 g/dl, the dose will be maintained.

The maintenance dose should be individualized and the recommended total weekly dose should not exceed 300 IU/kg.

<u>Dose adjustment scheme</u>

If the hemoglobin increases by more than 1 g/dl in any 2-week period or the hemoglobin is approaching 12 g/dl, the dose should be reduced by approximately 25%. If the hemoglobin continues to increase or exceed 12 g/dl, the treatment should be temporarily withheld until the hemoglobin begins to decrease, at which point therapy should be reinitiated at a dose approximately 25% below the previous dose. If dose reduction is needed, the amount given per dose or the number of weekly injections will be reduced, or both will be reduced.
If the hemoglobin is below 10 g/dl, the dose should be increased by approximately 25%.
When a change in Eprex® or PDA10 dose is indicated, a one-step change (25% of current dosage) is performed according to the stepwise dosing schedule refer to appendix III.
The dose adjustment scheme in Open-Label Extension phase is same as maintenance phase, but hemoglobin will be assessed every 4 weeks.</td></tr>
<tr><td>Methodology</td><td>This study is a multi-center, multi-national, randomized, double-blind, active-controlled and parallel group clinical trial. Eligible subjects will be randomized, using IWRS, to receive one of the two study treatment regimen (PDA10 or Eprex®) in a 1:1 ratio. All subjects are to continue their treatment PDA10 or Eprex®, and to receive study medication 1-3 times weekly intravenously (refer to Appendix III).

During 12-week of the titration phase, all subjects will receive Eprex®, then</td></tr>
</table>

CLINICAL STUDY SYNOPSIS: Study PG-EPO-Ph3	
	those with hemoglobin within the target range of 10.0 to 12.0 g/dl and a stable IV dose (a dosage titrated and individualized) of Eprex® at baseline period will be randomly assigned to one of two study drugs, either to the test product, PDA10, or to the reference product, Eprex®. During the maintenance phase, the efficacy and safety of two groups will be evaluated every 2 weeks or 4 weeks.<br><br>Subjects who complete the maintenance phase will be offered the choice of participating 24-week open-label extension phase. During the open -label extension phase, PDA10 will be administered and the long-term safety will be evaluated.
**Primary endpoint(s):**	• Mean change in hemoglobin level between the baseline period and the evaluation period<br>• Mean change in weekly dosage per kg body weight between the baseline period and the evaluation period<br>* The baseline period is the last four weeks of titration phase or the observation period and the evaluation period is the last four weeks of maintenance phase. Hemoglobin, hematocrit and the dosage of epoetin for the baseline period will be collected at Week -4, Week -2 and Week 0, and those for the evaluation period will be collected at Week 24, Week 26 and Week 28.
**Secondary endpoint(s):**	• Mean change in hematocrit levels between the baseline period and the evaluation period<br>• Proportion of patients with hemoglobin level out of the target range during the maintenance phase<br>• Proportion of patients with hemoglobin level within the target range during the evaluation period<br>• Frequency of patients with changes in the dosage per kg body weight<br>• Incidence of blood transfusions
**Safety endpoint(s):**	1) Adverse Events<br>2) Occurrence of anti-epoetin antibodies<br>3) Vital Signs<br>4) Physical Examination<br>5) Clinical Laboratory Determinations

CLINICAL STUDY SYNOPSIS: Study PG-EPO-Ph3	
**Statistical methods**	**Efficacy Analysis**<br><br>Primary Endpoints:<br>From the statistical point of view, the question of therapeutic equivalence will be approached by calculating the upper limit and the lower limit of 95% one-sided confidence interval of the difference between both treatment groups of the primary endpoints. To demonstrate the therapeutic equivalence, two one-sided test (TOST) procedures will be used. It decomposes the interval hypothesis into two one-sided parts – H_0: $d < d_L$ and H_1: $d > d_U$, where d is the difference of the mean change in each primary endpoint, d_L is the lower limit of 95% one-sided confidence interval and d_U is the upper limit of 95% one-sided confidence interval. Both of these one-sided intervals will be compared with the pre-defined clinical acceptance ranges for the corresponding parameters (± 0.5 g/dl for hemoglobin and ± 45 IU/kg/week for epoetin dosage, based on the respective reference means). The intervals will be calculated by means of ANCOVA model including treatment group as a factor and study center and baseline value as covariates.<br><br>Secondary Endpoints:<br>Summaries of categorical variables (norminal or ordinal) will include frequency and percentage of patients at each level of response. Continuous variable summaries will include n, mean, median, standard deviation, minimum and maximum values. Treatment difference will be analyzed using two sample t-test or Wilcoxon rank-sum test for continuous parameters, and using χ^2-test or Fisher's exact test for categorical parameters.
**Statistical methods**	**Safety Analysis**<br><br>Adverse Event:<br>The number, percentage and the corresponding 95% CI of patients experiencing TEAEs in each treatment group will be presented. The significance test for treatment difference will be carried out using χ^2-test or Fisher's exact test.<br><br>Clinical laboratory test, Vital signs,<br>Descriptive statistics for vital signs and changes from baseline at each visit will be presented by treatment group. A supportive listing of patients with clinically significant abnormal postbaseline values will be provided, including the Patient ID, study center, and baseline and postbaseline values.

3. TABLE OF CONTENTS

1. TITLE PAGE 1

2. SYNOPSIS 4

3. TABLE OF CONTENTS 11

4. LIST OF ABBREVIATIONS 13

5. ETHICAL CONSIDERATIONS 15
   5.1. Institutional Review Board 15
   5.2. Ethical Conduct of the Study 15
   5.3. Subject Information and Informed Consent 15

6. INVESTIGATORS AND STUDY ADMINISTRATIVE STRUCTURE 16

7. INTRODUCTION 18
   7.1. Background for Development 18
   7.2. Target Disease and Status of Therapy 18
   7.3. Clinical Data 19
   7.4. Risk and Benefits 19

8. STUDY OBJECTIVES 20

9. INVESTIGATIONAL PRODUCTS 21
   9.1. Overall Study Design and Plan: Description 21
      9.1.1. Test drug 21
      9.1.2. Reference drug 21
   9.2. Labeling for Study Drug 22
   9.3. Drug Supplies and Accountability 22

10. INVESTIGATIONAL PLAN 23
   10.1. Overall Study Design and Plan: Description 23
   10.2. Discussion of Study Design, Including the Choice of Control Groups 24
   10.3. Selection of Study Population 24
      10.3.1. Inclusion Criteria 24
      10.3.2. Exclusion Criteria 25
      10.3.3. Temporary Discontinuation of the Investigational Products 26
      10.3.4. Withdrawal From Therapy or Assessment 27
      10.3.5. Replacement Procedures 27
   10.4. Treatments 28
      10.4.1. Treatments Administered 28
      10.4.2. Identity of Investigational Product(s) 28
      10.4.3. Method of Assigning Subjects to Treatment Groups 29
      10.4.4. Selection of Dosages in the Study 30
      10.4.5. Blinding 31
      10.4.6. Prior and Concomitant Therapy 32
      10.4.7. Treatment Compliance 32
      10.4.8. Expected Adverse Events and Precautions for Use 32
   10.5. Study Procedures 34
      10.5.1. Screening Assessments 34
      10.5.2. Efficacy Assessments 34
      10.5.3. Safety Asseements 35
      10.5.4. Immunological Assessment 39
      10.5.5. Schedule of Procedures/Assessments 40
   10.6. Statistical Methods 52
      10.6.1. General Principles of the Result Analysis 52

10.6.2. Study Populations 52
10.6.3. Demographics and Clinical History Data 52
10.6.4. Efficacy Assessment 53
10.6.5. Safety Assessment 54
10.6.6. Immunology 55
10.6.7. Determination of the Sample Size and it's Rationale 55
10.6.8. Handling of Dropouts of Missing Data 56
10.6.9. Interim Analysis 57
10.7. Changes in the Conduct of the Study or Planned Analyses 57
10.7.1. Changes in the Conduct of the Study 57
10.7.2. Planned period of the Study 57

11. QUALITY CONTROL AND QUALITY ASSURANCE 58
11.1. Monitoring 58
11.2. Audit and Inspection 58

12. PUBLICATION AND PRESENTATION 59

13. INTELLECTUAL PROPERTIES 59

14. DATA QUALITY ASSURANCE 59
14.1. Data Monitoring 59
14.2. Data Recording and Documentation 59

15. SUBJECT CONFIDENTIALITY 60

16. MEDICAL CARE AND TREATMENT FOLLOWING STUDY COMPLETION 60

17. DUTIES 60
17.1. Investgator's Duties 60
17.2. Sponsor's Duties 61

18. REFERENCES 62

19. APPENDICES 64
Appendix I. Declaration Of Helsinki 65
A. INTRODUCTION 65
B. PRINCIPLES FOR ALL MEDICAL RESEARCH 65
C. ADDITIONAL PRINCIPLES FOR MEDICAL RESEARCH COMBINED WITH MEDICAL CARE 67
Appendix II. Iron Status and Therapy 69
Appendix III. Epoetin Dosing 70
Appendix IV. List of study personnel and contact details 71

20. Summary of Protocol Amendments and Administrative Changes to the Protocol 72

4. LIST OF ABBREVIATIONS

AE	adverse event
ALT/AST	alanine aminotransferase/aspartate aminotransferase
ANCOVA	analysis of covariance
ANOVA	analysis of variance
BP	blood pressure
bpm	beats per minute
BUN	blood urea nitrogen
CRC	clinical research coordinator
CRF	chronic renal failure
CRO	contract research organization
CRP	C-reactive protein
eCRF	electronic case report form
EDC	electronic data capture
EPO	erythropoietin
GCP	good clinical practice
Hb	hemoglobin
Hct	hematocrit
HIV	human immunodeficiency virus
ICF	informed consent form
ICH	International Conference on Harmonization of Technical Requirements for Registration of Pharmaceuticals for Human Use
IEC	Independent Ethics Committee
IRB	Institutional Review Board
ISF	Investigator’s Site File
ITT	intent to treat
IU	international unit
IV	intravenously
IWRS	interactive web response system
LOCF	last observation carried forward
NYHA	New York Heart Association
PCS	potentially clinically significant

PFS	pre-filled Syringe
PI	Principal Investigator
PRCA	pure red cell aplasia
PT/PTT	prothrombin time/partial thromboplastin time
PTH	parathyroid hormone
RBC	red blood cell
rhEPO	recombinant human erythropoietin
SAE	serious adverse event
SC	subcutaneous
SUSARs	suspected unexpected serious adverse reactions
TEAE	treatment-emergent adverse event
TSAT	transferrin saturation
WBC	white blood cell
β-hCG	β-human chorionic gonadotropin

5. ETHICAL CONSIDERATIONS

This clinical study is designed to conduct and evaluate the study and record the results in ethical and scientific consideration complying with the International Conference on Harmonization (ICH) Guidance on General Considerations for Clinical Trials, Good Clinical Practice, and the related regulations.

5.1. INSTITUTIONAL REVIEW BOARD

Approval by the Institutional Review Board (IRB)/Independent Ethics Committee (IEC) before the start of the study will be the responsibility of the Principal Investigator (PI). A signed letter of study approval from the IRB/IEC chairman must be sent to the PI with a copy to the Sponsor before study start and the release of any study drug to the site by the Sponsor or its designee. If the IRB/IEC decides to suspend or terminate the study, the Investigator will immediately send the notice of study suspension or termination by the IRB/IEC to the Sponsor.

Study progress is to be reported to IRB/IECs annually (or as required) by the Investigator or Sponsor, depending on local regulatory obligations. If the Investigator is required to report to the IRB/IEC, he/she will forward a copy to the Sponsor at the time of each periodic report. The Investigator(s) or the Sponsor will submit, depending on local regulations, periodic reports and inform the IRB/IEC of any reportable adverse events (AEs) per ICH guidelines and local IRB/IEC standards of practice. Upon completion of the study, the Investigator will provide the IRB/IEC with a brief report of the outcome of the study, if required.

At the end of the study, the Sponsor should notify the IRB/IEC within the day to be required. The end of the study will be the date of the last study visit for the last subject in the study (or the end of the study will be the date of the clinical database lock, i.e., when all study data are collected and data validation is completed.). The Ssponsor should also provide the IRB/IEC with a summary of the study's outcome.

In the case of early termination/temporary halt of the study, the Investigator should notify the IRB/IEC within 24 hours, and a detailed written explanation of the reasons for the termination/halt should be given.

5.2. ETHICAL CONDUCT OF THE STUDY

The study will be conducted in full compliance with standard operating procedures of the Sponsor (or designee), which are designed to ensure adherence to Good Clinical Practice (GCP) guidelines and in accordance with the ethical principles that have their origins in the Declaration of Helsinki and ICH E6 Guideline for GCP.

5.3. SUBJECT INFORMATION AND INFORMED CONSENT

If adverse events requiring treatment occur, the subject will be given the specialist's diagnosis and appropriate treatment. The subject who discontinued the study will be guided to receive other appropriate medical treatment.

It is strictly evaluated whether the subject is eligible for this study through the pre-treatment tests. The study is conducted according to the protocol. At every visit during the study, patients are to be assessed with adverse events and adverse drug reactions occurred and their severity should be evaluated along with the appropriate measures taken.

The site should be equipped with the facilities and professionals required to conduct this study and every effort should be made to protect subjects.

If necessary, the Sponsor purchases the insurance policy and provides a copy of the policy for the Investigator.

Each Investigator has all the ethical and legal responsibilities to guarantee the patients who may be subjected to this study can be given full explanation about the protocol. This should be recorded in the informed consent form in writing which should be approved by the IRB/IEC, the same IRB/IEC which is responsible for approving this protocol. Each informed consent form should include the factors required by the ICH CGP Guidelines and local regulations, and comply with the ethical principles based on the Declaration of Helsinki. The Investigator agrees to receive an approval of any informed consent form used in this study by the Sponsor before submitting the form to the IRB/IEC.

The written consent will be obtained from all the subjects. The Investigator may discuss with a prospective subject concerning the study without obtaining consent.
When appropriate basic information is provided for the subjects, it is explained by the Investigator in a simple and non technical manner, the written consent form approved by the IRB/IEC should be signed and dated by the subjects, the person who obtains the consent (Investigator or delegate) and any other personnel requested by IRB/IEC. The subjects should receive one copy of the signed consent form and an original copy should be retained in the Investigator's Site File (ISF) by the Investigator. All the activities mentioned above should be completed before the subjects participate in the study.
If new information requiring the amendment of the informed consent form is secured, the Investigator should promptly consult with the Sponsor and conduct the necessary amendment according to the information. If necessary, the Investigator will report this amendment to the head of the site and submit the amended document to the IRB/IEC for approval. The Investigator should obtain the informed consent form from the subjects again using the amended informed consent form and explained information, he/she should follow the procedures described by the site.

1) The Investigator should provide enough information for the subjects about all the tests related to this study including the information which the IRB/IEC approved in writing for the subjects considered to be included in the study and their legal representatives.

2) The subject or his/her legal representative and the person who conducts the subject consent procedures will sign and date the informed consent form. A copy of the signed and dated consent form will be provided for the subject.

3) The Investigator should not conduct any study specific tests until informed consent from the subject has been obtained.

4) The investigator should retain the list of all the patients considered as a subject who have been screened and all the subjects who signed the consent form.

6. INVESTIGATORS AND STUDY ADMINISTRATIVE STRUCTURE

This study will be performed at approximately 20 study centers located in Korea and Malaysia. At each study center, the PI will be responsible for ensuring that the investigation is conducted according to the signed Investigator agreement, the protocol, and good clinical practice guidelines.

The PI at each study center will be responsible for the management of the study, which will consist of maintaining the study file and the patient records, corresponding with the IRB, and completing the electronic case report forms (eCRFs).

<u>List of study personnel and contract details, please refer to Appendix IV.</u>

7. INTRODUCTION

7.1. BACKGROUND FOR DEVELOPMENT

Chronic kidney disease (CKD), also known as chronic renal disease, is a progressive loss in renal function over a period of months or years.
Recent professional guidelines classify the severity of CKD in five stages, with stage 1 being the mildest and usually causing few symptoms and stage 5 being a severe illness with poor life expectancy if untreated. Stage 5 CKD is also called established chronic kidney disease and is synonymous with end stage renal disease (ESRD), chronic kidney failure (CKF) or chronic renal failure (CRF) (National Kidney Foundation. 2002).

Decreased renal function interferes with the kidneys' ability to maintain fluid and electrolyte homeostasis, and it leads to one of its recognized complications, such as cardiovascular disease, anemia or pericarditis. Severe CKD requires treatments of anemia, and renal replacement therapy, which may involve a form of dialysis (National Kidney Foundation. 2002). Additionally, anemia, a common complication of CKD, frequently develops as a result of a decrease in the ability of the kidneys to produce the glycoprotein hormone erythropoietin (Nangaku M et al. 2006 and Eckardt KU 2000) and occurs relatively early during the progression of CKD (Levin A. 2001 and Astor BC et al. 2002). Patients with a hemoglobin concentration of < 8 g/dl usually have either end stage renal failure or another condition contributing to their anemia, such as myeloma (MacDougall et al. 1990).

The development of erythropoiesis-stimulating agents has been beneficial in treating the anemia of CRF and an integral part of anemia management by maintaining hemoglobin (Hb) levels, so reducing blood transfusion rates and improving quality of life (Eschbach JW et al. 1989 and Lefebvre P et al. 2006). Improvements in anemia management have been the subject of clinical trials with new agents and treatment regimens which attempt to demonstrate potential advantages to patients and health care providers without compromising efficacy or safety, particularly in regard to Hb levels and Hb increase over time. An ideal agent would provide a smooth correction in Hb levels and then maintain Hb within the target range at a reduced dose frequency.

Human erythropoietin is a glycoprotein which is produced primarily in the kidneys and promotes red blood cell (RBC) production by stimulating the division and differentiation of committed progenitors in the bone marrow. Erythropoietin for clinical use is produced by recombinant DNA technology using mammalian cells as expression system. Epoetin-containing medicinal products are currently indicated for several conditions such as anemia in patients with chronic renal failure, chemotherapy-induced anemia in cancer patients and for increasing the yield of autologous blood. Recombinant human erythropoietin has been proved beneficial in treating renal anemia (Eschbach JW et al. 1988, Eschbach JW. 1989, Winearls CG. 1989 and Schaefer RM et al. 1989), and clinical trials have indicated an acceptable degree of safety (Winearls CG et al. 1986, Eschbach JW et al. 1987, Bommer J et al. 1987, Casati S et al. 1987 and Eschbach JW' et al. 1989).

7.2. TARGET DISEASE AND STATUS OF THERAPY

Hemodialysis is the most common method used to treat advanced and permanent kidney failure. Most experience of treatment with erythropoietin is with intravenous therapy in patients receiving hemodialysis. Hemodialysis itself, however, may contribute to the anemia, and iron deficiency can result from unavoidable dialyzer blood loss, clotted dialysis membranes, and frequent blood sampling.

It is important to determine clearly the baseline iron state of any patient being considered for erythropoietin treatment, and iron state should be monitored in patients who require dialysis (have stage 5 CKD) (MacDougall IC et al. 1996). If a patient is iron deficient, then some improvement in hemoglobin concentration can be expected to occur with iron treatment alone, either oral (Strickland ID et al. 1974) or intravenous (Strickland ID et al. 1977). As a result, treatment with erythropoietin

should be withheld until it is clear that the patient is not iron deficient or until a deficiency has been fully treated, as evidenced by a stable hemoglobin concentration. The advent of erythropoietin has resulted in an unprecedented and potent therapeutic stimulus to erythropoiesis, and it has become apparent that large quantities of iron are used in this process. Furthermore, patients who are iron replete before starting the hormone can rapidly become deficient under the influence of this treatment (Eschbach JW et al. 1977, Eschbach JW et al. 1987, Eschbach JW et al. 1989, Van Wyck DB et al. 1989, and Frenken LAM et al. 1988). Transferrin saturation should be at least 20%, and serum ferritin levels should be at least 100 ng/ml.

Human erythropoietin is a single chain, monomeric, glycosylated polypeptide of 165 amino acids. Erythropoietin for clinical use is produced by recombinant DNA technology using mammalian cell as expression system. All epoetins in clinical use have an amino acid sequence similar to endogenous erythropoietin but differ in the glycosylation pattern. Glycosylation influences pharmacokinetics and may affect efficacy and safety, particularly immunogenicity.

PDA10 is a biosimilar erythropoiesis stimulating agent indicated for the treatment of anemia associated with CKD. The active substance in PDA10 is a recombinant human erythropoietin (rhEPO) of identical primary structure produced in Chinese Hamster Ovary (CHO) cells (CHO-DG44).

7.3. CLINICAL DATA

The phase 1 study of PDA10 was conducted with healthy male adult volunteers, and the results refer to the 'Investigator's Brochure.

7.4. RISK AND BENEFITS

The risks of this clinical study are essentially the risk of experiencing an adverse event following administration of PDA10.

The anemia of chronic renal failure can be corrected fully with erythropoietin. In balancing the benefits and the risks, however, the common practice is to aim at partial correction. A linear increase in the hemoglobin concentration or packed cell volume leads to an exponential rise in the viscosity of whole blood (Schaefer RMl et al. 1988, Mayer G et al. 1988, and MacDougall IC et al. 1989), which, in turn, is thought to contribute to many of the adverse events of treatment with erythropoietin such as hypertension, increased peripheral resistance, thrombotic complications, etc. However, this partial correction of the anemia seems to cause near maximal improvement in wellbeing, exercise capacity (Mayer G et al. 1988), and symptoms of anemia. Moreover, the use of erythropoietin has been associated with a reduced need for blood transfusion, reduction in the frequency and severity of anemia-associated morbidity, and improvement in quality of life

The target hemoglobin concentration most commonly used is in the range of 10.0-12.0 g/dl, at which the ratio of risk to benefit seems to be minimized, though some flexibility is necessary in treating individual patients. Because the main aim of treatment with erythropoietin is to reverse the symptoms of anemia, differing thresholds at which this occurs may influence the appropriate final hemoglobin concentration.

With regard to the rate of rise of the hemoglobin response, for most patients an increase of 1 g/dl per month seems sensible. Exceeding this limit may predispose the patients to an increase risk of adverse events, and there is rarely any indication for more rapid correction of anemia.

On the other hand, PDA10 has been never tried in patients with CRF. Therefore, the design of this study excludes the subjects who are considered to have risks based on the current clinical procedures and eliminate the risk of the subjects as much as possible through the strict safety monitoring.

8. STUDY OBJECTIVES

1. The primary objective is to demonstrate the therapeutic equivalence of PDA10 to Eprex® in patients with end stage renal failure (ESRD) on chronic hemodialysis.

   The therapeutic equivalence shall be evaluated by the difference between treatment groups in the change in hemoglobin from baseline, while taking into account differences in epoetin dosage.

2. The secondary objective is to assess the safety and tolerability of PDA10

3. The aim of the open-label extension phase is to assess the long-term safety and tolerability of PDA10

9. INVESTIGATIONAL PRODUCTS

9.1. OVERALL STUDY DESIGN AND PLAN: DESCRIPTION

9.1.1. Test drug

- Product name: PDA10 Pre-filled syringe injection 2000 IU
- Generic name: Epoetin-alfa
- Appearance and formulation: A pre-filled syringe containing colorless transparent liquid with a needle included
- Storage: Keep refrigerated at 2 ~ 8℃
- Expiration date: 18 months from the date of manufacture
- Strength: 2,000 units (0.5 mL)

Quantities of ingredients: One pre-filled syringe (0.5 mL) contains the following:

	Name of ingredient	Acceptance Criteria	Quantity
Active ingredient	Recombinant human epoetin-alfa (vector :pMSG-EPO, host cell:CHO-DG44/dhfr-)	In-house	2,000 units (16.8 µg)
Stabilizer	Polysorbate 80	Ph.Eur.	q.s.
Stabilizer	Glycine	Ph.Eur.	q.s.
Isotonic agent	Sodium Chloride (NaCl)	Ph.Eur.	q.s.
Buffer	Sodium phosphate monobasic dihydrate ($NaH_2PO_4.2H_2O$)	Ph.Eur.	q.s.
Buffer	Sodium phosphate dibasic dihydrate ($Na_2HPO_4.2H_2O$)	Ph.Eur.	q.s.
Solvent	Water for injection	KP	q.s.

9.1.2. Reference drug

- Product name: Eprex® (2000 IU/0.5 mL)
- Manufacturer: Janssen-Cilag
- Generic name: Epoetin-alfa
- Appearance and formulation: A pre-filled syringe containing colorless transparent liquid with a needle included
- Storage: Keep refrigerated at 2 ~ 8℃
- Expiration date: 18 months from the date of manufacture
- Strength: 2,000 units (0.5 mL)

Quantities of ingredients: One pre-filled syringe (0.5 mL) contains the following:

	Name of ingredients	Acceptance criteria	Quantity
Active ingredient	Recombinant human epoetin-alfa (vector and host cell used: N/A)	Ph.Eur.	2,000 units (16.8 µg)
Stabilizer	Polysorbate 80	Ph.Eur.	q.s.
Stabilizer	Glycine	Ph.Eur.	q.s.
Isotonic agent	Sodium Chloride (NaCl)	Ph.Eur.	q.s.
Buffer	Sodium phosphate monobasic dihydrate ($NaH_2PO_4.2H_2O$)	Ph.Eur.	q.s.
Buffer	Sodium phosphate dibasic dihydrate ($Na_2HPO_4.2H_2O$)	Ph.Eur.	q.s.
Solvent	Water for injection	Ph.Eur.	q.s.

9.2. LABELING FOR STUDY DRUG

On the label of the investigational product, the following matters will be stated according to the relative laws including randomization number and medication ID.

- Mark of “Investigational drug”
- Product code or generic name
- Lot number and expiration date or retest date
- Storage condition
- Name of Sponsor or IND holder
- Mark of “cannot be used for the purposes other than the clinical study”

9.3. DRUG SUPPLIES AND ACCOUNTABILITY

The Sponsor (or the delegated person) should directly dispense the investigational product to the study pharmacist (or the delegated person) after consultation with the Principal Investigator and retain the delivery form. The frequency of supply of the investigational products for each site will be adequately regulated according to the status of the subject enrollment at each site and the expiry date of the investigational products. The investigational products will be provided in the form of pre-filled syringes.

Investigational product should be stored at 2-8°C and protected from light.

Investigational product should be stored in a secured, limited access area. The Investigator and hospital pharmacist are responsible for the appropriate storage and documentation of proper storage conditions of the investigational product at the site. The Sponsor (or the delegated person) should confirm the quantity and storage condition of the investigational products and manage them for the adequate process of the study.

The study pharmacist (or the delegated person) should keep track of receipt, shipment and condition of the investigational products. For all the investigational products, he/she should record the number of dispensed and returned products and the details including dispensation and return dates in the form (“Drug Accountability Log”) supplied by the Sponsor (or the delegated person). The study pharmacist (or the delegated person) should retain the record of the receipt, dispensation and return of the investigational products. Details of any drug lost (due to breakage or wastage), not used, deposed of at the study center or returned, must also be recorded. All supplies and pharmacy documentation must be made available throughout the trial for Monitor or sponsor delegated person to verify.

The investigational products and supplies cannot be used for purposes other than the purpose described in the protocol.

The PI or study pharmacist at each site should retain all the returned and unused investigational products until the monitor of the Sponsor (or the delegated person) confirms them. All the returned and unused investigational products will be collected or disposed by the Sponsor (or the delegated person) at the completion of the study or at the request of the Sponsor (or the delegated person). After consultation with the Principal Investigator, the study pharmacist should return the returned and unused drugs to the Sponsor (or the delegated person) and retain the return receipt.

If the investigational products must be disposed at the site during the study, the PI or study pharmacist must retain the returned and unused investigational products until the Sponsor (or the delegated person) approves of the disposal. If the investigational products are disposed at the site after the Sponsor’s confirmation and approval, the PI or study pharmacist is responsible to record and document it in detail.

10.INVESTIGATIONAL PLAN

10.1. OVERALL STUDY DESIGN AND PLAN: DESCRIPTION

This clinical study will be a multi-center, multi-national, randomized, double-blind, active-controlled and parallel group clinical trial. This study will consist of a 12-week of titration phase designed to assess the stability of patients' disease and to establish each patient's baseline characteristics. In substantiated cases, a prolongation of the titration phase will be allowed up to 20 weeks. The titration phase will be followed by a 28-week double-blind treatment phase (maintenance phase) including the evaluation period (the last 4 weeks of the maintenance phase). Eligible patients will be randomized to receive one of the two study treatment regimen (PDA10 or Eprex®) in a 1:1 ratio according to the randomization scheme. All subjects are to continue their treatment PDA10 or Eprex®, and to receive study medication 1-3 times weekly intravenously (IV).

Prior to performing any procedures or evaluation, written informed consent will be obtained. Prospective patients will be screened to confirm their eligibility for the study entry prior to randomization. During the 12-week of the titration phase, all subjects will receive Eprex®, then those with hemoglobin within the target range of 10.0 to 12.0 g/dl and a stable IV dose (a dosage titrated and individualized) of Eprex® during baseline period will be randomly assigned to one of two study drugs, either to the test product, PDA10, or to the reference product, Eprex®. However, patients on Eprex® treatment for at least 12 weeks and with hemoglobin within the target range of 10.0 to 12.0 g/dl and a stable IV dose of Eprex® will participate in the process of confirming the entry criteria for this study, and have a four-week of the baseline period (or observation period) without the titration phase. During the maintenance phase, the efficacy and safety will be evaluated every 2 or 4 weeks.

Subjects who complete the study at Week 28 will be offered continuation of PDA10 treatment by entering a 24-week of open-label extension phase, and the long-term safety and tolerability will be evaluated. Subjects must complete the Week 28 (Visit 11) assessments before entering the open-label extension phase. Visit 11 at Week 28 of the study will serve as the entry visit for the open label extension phase.

Figure 10.1–1. ***Study Design Diagram***

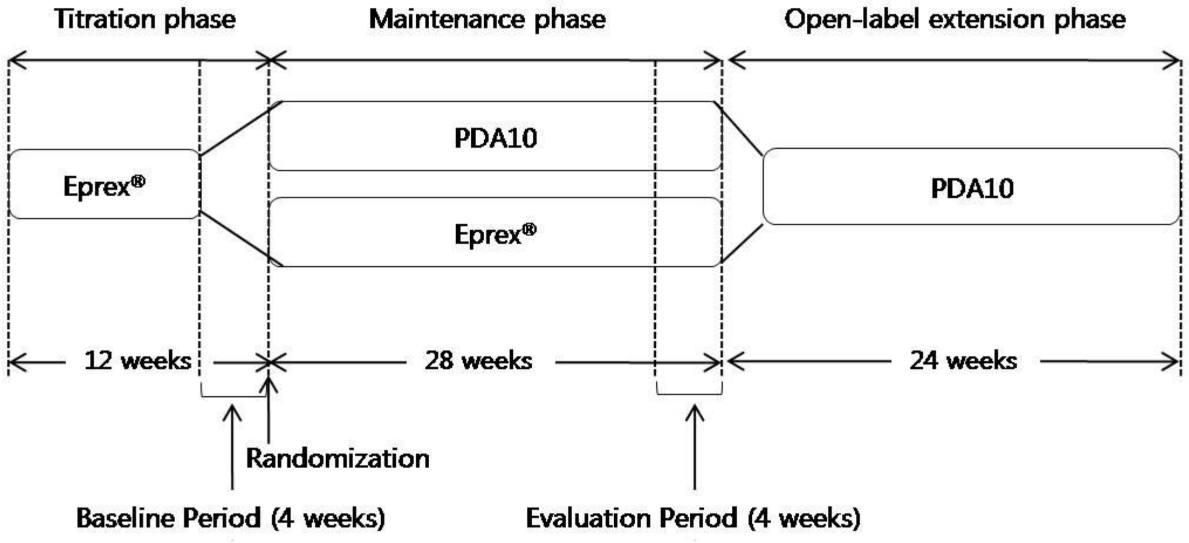

10.2. DISCUSSION OF STUDY DESIGN, INCLUDING THE CHOICE OF CONTROL GROUPS

This study is designed to evaluate the efficacy and safety of PDA10 and Eprex® administered by intravenous route.

Randomization will be used in this study to avoid bias in the assignment of subjects to treatment, to increase the likelihood that known and unknown subject attributes (e.g., demographics and baseline characteristics) are balanced across treatment groups, and to ensure the validity of statistical comparisons across treatment groups. Blinding to treatment will be used to reduce potential bias during data collection and evaluations of endpoints.

The double-blind and parallel-group design is considered to be appropriate for the comparison of efficacy and safety in patients receiving PDA10 and patients receiving Eprex® . The intended sample size has been selected to achieve sufficient power for comparisons of efficacy.

Subjects, who have completed the maintenance phase and want to continue the study participation, can be enrolled in the 24-week open-label extension phase. All subjects who provide signed informed consent to participate in open-label extension phase will be assigned to receive PDA10. During this phase, the long term safety, tolerability and immunogenicity will be evaluated.

Hemoglobin level has been utilized as the primary efficacy endpoint since it is a measure of the amount of hemoglobin per unit volume of blood and provides a measure of the ability of blood to transport oxygen to the tissues. Additionally, it is a well-established measure of efficacy in the treatment of patients with anemia of chronic renal failure. It is commonly used in clinical practice to assess the severity of anemic disorders and is one of the factors considered by physicians in determining whether to prescribe treatment, such as a blood transfusion, to patients with anemia, including sickle cell anemia. Epoetin dose is titrated to achieve the desired response to reduce the sensitivity of the hemoglobin-related endpoints to detect possible differences in the efficacy of the treatment arms. Therefore, epoetin dosage is a co-primary endpoint for this study.

Erythropoietin (EPO) promotes the use of iron deposits in the bone marrow, and consequently iron deficiency is a frequent problem resulting in resistance to the full effect of EPO. It is well known that a negative iron balance may occur (Eschbach JW et al. 1977) and it is not well established whether there is a compensatory increase in intestinal absorption in hemodialysis patients when they are iron deficient (Goch J et al. 1996, Donelli SM et al. 1991, Macdougall IC et al. 1995, and Gokal R et al, 1979). Oral supplementation is plagued by poor patient adherence due to frequent adverse events, and interference of other medication with digestive absorption (Marchasin S et al. 1961). Several studies have demonstrated the utility of IV iron supplementation in correcting anemia and sparing epoetin. Based on the above, intravenous iron agent will be supplemented with PDA10 and Eprex® as appropriate. See Appendix II for iron status and therapy.

10.3. SELECTION OF STUDY POPULATION

10.3.1. Inclusion Criteria

To be eligible to participate in the study, patients must meet the following criteria:

1. Anemic patients with end stage renal failure (ESRD) on chronic hemodialysis
2. Patients must be at least 18 years old but less than 75 years old at Screening Visit
3. Patients on hemodialysis through a functioning native arterio-venous fistula

4. Patients must be at their dry body weight or within 5% of it during the baseline period (observation period)

5. Patients must be able to understand the information provided to them and to give written Informed Consent

6. Female patients of childbearing potential must have a negative serum pregnancy test at the Screening Visit and at Baseline Visit. Females must be surgically sterile, postmenopausal for at least 1 year prior to Screening Visit or must be using an acceptable method of birth control ([oral, non-oral or implantable] hormone contraceptives, intrauterine contraceptive device or blockers and spermicides) effectively. Abstinence is not an acceptable method of contraception for the study.

7. Patients must have the following at Screening Visit or prior to randomization as well as at the baseline period (or observation period)

   - Patients on erythropoietin treatment prior to Screening
     Note: If patients who have been on Eprex® treatment before this study have hemoglobin level less than 10 g/dl during the baseline period (or observation period), they must participate in the titration phase.

   - Hemoglobin level with 10 - 12 g/dl and a stable IV dose of Eprex® without transfusion prior to randomization (A stable IV dose is defined as not more than 25% change up or down in weekly dose and no clinically relevant change of regimen in frequency of haemodialysis for the baseline period [observation period])

   - Patients on Eprex® treatment for at least 12 weeks; patients on stable and adequate hemodialysis at least 3 times a week and with a documented URR > 65% or delivered KT/V ≥ 1.2 in the past 6 months prior to randomization (If patients meet this criteria, they will be randomized without participating in the titration phase)

   - Serum ferritin level at least 100 ng/ml and/or transferrin saturation (TSAT) at least 20% prior to randomization

10.3.2. Exclusion Criteria

Patients who meet any of the following criteria will not be eligible to participate in the study**:**

1. Patients with a temporary or permanent catheters or synthetic grafts

2. Patients with uncontrolled hypertension, defined as a pre-dialysis diastolic BP of greater than 110 within 12 weeks prior to randomization

3. Patients with severe disease within the last 6 months prior to randomization (e.g stroke, transient ischemic attack, myocardial infarction, cerebrovascular accident, coronary artery bypass graft deep venous thrombosis, unstable angina, decompensate congestive heart failure (New York Heart Association [NYHA] class III~IV), or other thromboembolic event

4. Surgery patients who for any reason cannot receive adequate antithrombotic prophylaxis or treatment

5. Patients with a current or recent known history of a severe hyperparathyroidism or PTH > 1500 pg/ml within 12 weeks prior to randomization.

6. Patients with hyperkalemia
7. Patients with epilepsy
8. Patients with malnutrition (serum albumin < 3.5g/dl prior to randomization)
9. Patients with an acute infection, acute hepatitis (including A, B, C type) or chronic hepatitis B or C requiring treatment or HIV infection
10. Patients with significant inflammation (CRP >30 mg/L within 12 weeks prior to randomization)
11. Patients with a history of gastrointestinal bleeding within the last 6 months before Screening
12. Patients with any active, uncontrolled systemic or inflammatory disease that in the Investigator's opinion may be significant to exclude participation in the study
13. Patients of need for blood transfusions within 12 weeks prior to randomization
14. Patients with history of pure red cell aplasia (PRCA) or anti-erythropoietin antibodies
15. Patients with a history of malignancy of any organ system within the last 5 years prior to Screening
16. Patients with a current diagnosis of anemia due to folic acid and/or Vitamin B12 deficiencies, hemolysis, or gastrointestinal bleeding or a history of/or active blood or bleeding disorders within the last 6 months before Screening
17. Patients who have received immunosuppressive treatment or use of other medication known to influence erythropoiesis 12 weeks prior to randomization
18. Patients with hypersensitivity to the active substance or to any of the excipients
19. Patients who have treated with any other investigational drug within 4 weeks prior to Screening
20. Patients who currently are pregnant or lactating
21. Patients who are not cooperative or not able to follow the clinical study procedures
22. Patients who are judged to be ineligible to the clinical study at the Investigator’s discretion for other reasons such as alcohol and drug abuse

10.3.3. Temporary Discontinuation of the Investigational Products

The administration of the investigational product should be continued as much as possible. If the investigational product is discontinued, the Investigator should determine whether the drug can be only temporarily discontinued. The permanent discontinuation should be the last decision.

If the Investigator considers the corresponding adverse event onset is not related to the investigational product according to his/her best medical judgment, the re-challenge of the investigational product will be conducted under the careful and adequate clinical/laboratory monitoring.

The Investigator should record all the temporary discontinuation and re-challenge dates on the appropriate page of the eCRF.

10.3.4. Discontinuation From Therapy or Assessment

A premature discontinuation will occur when a subject who signed informed consent ceases participation in the study, regardless of circumstances, before the completion of all study visits. Subjects can be prematurely discontinued from the study for one of the following reasons:

- **Nonfufillment of inclusion/exclusion criteria (Screening Failure):** This reason applies if a patient is found not eligible for randomization at Screening (Visit 1) or at Baseline (Visit 4) before randomization. Screening failure subjects can be rescreened at any time after signing the informed consent form, and they will be given a new screening number.

- **An AE:** If a subject experiences an AE, premature discontinuation will be at the discretion of either the Investigator or the subject, independent of the event's relationship to the study treatment. The appropriate AE eCRF page must be completed.

- **Lack of efficacy:** This reason applies if response to the treatment is considered by the Investigator or the subject to be unsatisfactory.

- **Protocol noncompliance:** Any protocol violations detected during the trial should be corrected when possible and the subject allowed to continue. ONLY those violations that could affect the subject's safety (eg, illness requiring treatment[s] that in the clinical judgment of the Investigator [or after discussion with the medical monitor or the Sponsor] might invalidate the trial by interfering with the allocated double-blind study drug) or the willingness of the subject to comply with the study activities should lead to discontinuation.

- **Subject's request:** The subject is permitted to stop his/her participation at any time during the study without incurring any loss of his/her medical care. The Investigator should ensure that such withdrawal is not due to AEs or lack of efficacy, in which case those reasons should be selected.

- **Lost to follow-up:** Subjects who cannot be contacted and who do not have a known reason for discontinuation (e.g., withdrew consent or adverse event) will be classified as 'lost to follow-up' as the reason for discontinuation. In these cases, every effort should be made by the Investigator to ascertain the reason and to ensure the subject's attendance as soon as possible.

In case of withdrawal or discontinuation, unless informed consent has been withdrawn, the Investigator should make efforts to evaluate the withdrawal visit for all the subjets, together with a narrative description of the reason(s) for removing the subject, must be recorded in the source documents. The eCRF must document the primary reason for withdarwal or discontinuation.

10.3.5. Replacement Procedures

The subjects who were discontinued at the screening after signing the informed consent form can be re-screened in this study if they were not yet randomized. At this time, they must sign a new informed consent form.

If the randomized subject is withdrawn, he/she is not replaced with a new subject. The withdrawn subject's randomization number cannot be used again and the subject in question cannot participate in this study again.

10.4. TREATMENTS

10.4.1. Treatments Administered

The study drug will be provided by the Sponsor. During the study, patients will be supplied with Pre-filled Syringe (PFS) containing either PDA10 or Eprex®. All the PDA10 PFSs and Eprex® PFSs will have the same external appearance and will function in the same method.

After having been given full explanation about this study, the subject will sign the informed consent form. If the subject satisfies the inclusion/exclusion criteria at Screening (Visit 1), he/she will be administered Eprex® with iron supplement (if applicable) for 12 weeks. If the subject's hemoglobin level is within the target range (10.0 to 12.0 g/dl) at Baseline period (or observation period), he/she will be randomized to either PDA10 or Eprex® group at Baseline Visit. The subject randomized to either group will be treated with the investigational product for 28 weeks from the randomization in a double blind fashion.

Subjects who complete at Week 28 will be offered continuation of PDA10 treatment with iron supplement (if applicable) by entering the open-label extension phase. Subjects must complete the Week 28 (Visit 11) assessments before entering the open-label extension phase. Visit 11 at Week 28 of the maintenance phase will serve as the entry visit for the open-label extension phase.

- **Investigational product (PDA10 2,000 IU, PFS)**
- **Reference product (Eprex® 2,000 IU, PFS)**

In patients on hemodialysis, PDA10 or Eprex® will be administered as an IV bolus 1-3 times in a week. While the administration of PDA10 or Eprex® is independent of the dialysis procedure, PDA10 or Eprex® may be administered into the venous line at the end of the dialysis procedure to obviate the need for additional venous access. Alternatively, a bolus injection may be given during the dialysis session through a suitable venous port in the dialysis line.

Note:

1. The subcutaneous route of administration must not be used.

2. The maximum dosage should not exceed 300 IU/kg /dose.

Each site will be required to prepare, sign and retain a written delegation log signed by the PI for ensuring that the double-blind nature of the study is maintained.

10.4.2. Identity of Investigational Product(s)

The Sponsor will provide the study drugs packaged in a double-blind configuration in labeled containers and each subject's study drug will consist of either PDA10 or Eprex® during the maintenance phase. Each study center will be provided with drug supplies corresponding to a sequence of randomization numbers.

The properties of the investigational product provided by the Sponsor are listed in the table below.

PDA10 for intravenous injection is supplied in a pre-filled syringe (PFS).

Investigational Products	Appearance
Investigational Product: PDA10	Formulated as a sterile, colorless liquid in an isotonic sodium chloride/ sodium phosphate buffered solution in pre-filled syringe
Reference Product: Eprex®	Formulated as a sterile, colorless liquid in an isotonic sodium chloride/ sodium phosphate buffered solution in pre-filled syringe

Storage conditions

PDA10 and Eprex® are to be stored between 2°C and 8°C in the refrigerator, away from the freezer compartment and kept in the original carton. This temperature range should be closely maintained until administration to the subject.

The product should not be used, and discarded
- if the seal is broken,
- if the liquid is colored or particles floating is observed,
- if it may have been accidentally frozen, or
- if there has been a refrigeration failure

Note:

The investigational products should be visually inspected for particulate matter and discoloration prior to administration. Product exhibiting particulate matter or discoloration must not be used. The PFSs should not be shaked; shaking may denature the glycoprotein, rendering it inactive.

The investigational products contain no preservatives, and the syringe should not be reused and unused portion must be discarded.

IV Injection should be administered over 1-2 minutes. In subjects on dialysis the injection should follow the dialysis procedure. Slow injection over 5 minutes may be beneficial to those who experience flu-like symptoms.

10.4.3. Method of Assigning Subjects to Treatment Groups

At the time of the Baseline Visit, patients will be randomly assigned to receive either PDA10 or Eprex® according to the interactive web response system (IWRS) upon signing the ICF. This randomization number will consist of two parts as follows:

2-digit unique investigational site number established by Pangen Biotech

2-digit patient screening number consisting of sequential patient number unique within a site

For example, for a study center with number 99, the first consented patient 01 would have the following screening number 99S01 and the first randomized patient would have the following randomization number 99R01

The randomization number will be used to identify the subject throughout the study and will be recorded on the eCRF. This number must not be reused. Subjects who are rescreened will receive the next available randomization number to avoid confusion with data from the previous screenings.

10.4.4. Selection of Dosages in the Study

1) <u>Observation period</u>

In order to evaluate the Hb level of patients for screening, patients will be monitored on the Eprex® dosage previously treated for four weeks from screening visit and the Hb levels will be monitored every 2 weeks. When the Hb level and iron status meet the following criteria, the patients will proceed to the maintenance phase or the titration phase.

- **<u>Criteria to proceed the maintenance phase without titration phase</u>**

Patients on Eprex® treatment for at least 12 weeks and with hemoglobin within the target range of 10.0 to 12.0 g/dl and a stable IV dose of Eprex®, and adequate iron store (serum ferritin level ≥ 100 ng/mL and/or transferrin saturation ≥ 20%) during observation period will proceed to enter the maintenance phase. Hb level measured during this observation period will be considered as baseline level.

- **<u>Criteria to proceed the titration phase</u>**

1) Patients treated with other EPO products for at least 12 weeks

   If the patients are treated with other EPO products, the patients should proceed to the titration phase to exposure the Eprex® for at least 12 weeks.
2) Hb is below 10 g/dL even if the patients were treated with Eprex® for at least 12 weeks; in the event a patients' Hb is to decline below 10 during the observation period, patients should switch to titration phase.

2) <u>Titration phase</u>

The initial dosage of Eprex® will be the same with the erythropoietin dosage previously treated. However, the increased dosage will be treated for the initial dosage if Hb level is less than 10 g/dl prior to Screening Visit. The Hb level of patients is monitored every 2 weeks during titration phase. Eprex® dose changes will be made every four weeks on the basis of the most current Hb level—more frequent changes will be made only when the Hb value or the rate of Hb change is outside the preset safety as follows;

- If Hb level increase in the past two weeks is > 1.0 g/dL or Hb level is approaching 12 g/dL, the dose will be decreased by 25% of current dosage. If the hemoglobin continues to increase or exceed 12 g/dl, the treatment should be temporarily withheld until the hemoglobin begins to decrease, at which point therapy should be reinitiated at a dose approximately 25% below the previous dose. If dose reduction is needed, the amount given per dose or the number of weekly injections will be reduced, or both will be reduced.

- If Hb level increase in the past two weeks is < 0.3g/dL and Hb level is below 10 g/dL, the dose will be increased by 25% of current dosage.

- If Hb increase in the past 2 weeks is ≤ 1.0 g/dL and Hb level is in the target range (10 – 12 g/dL), the dose will be maintained.

When a change in Eprex® dose is indicated, a one-step change (25% of current dosage) is performed based on the stepwise dosing schedule refer to appendix III.

* The patients will be treated with iron therapy based on iron level as follows;

① Marginal iron store (all patients will have serum ferritin level > 100 to be eligible for entry) -

Eprex® dose increasing during the titration phase and IV iron as appropriate. For patients unable to access IV iron therapy, sponsor will support the iron therapy.

② Hb is below 10 g/dl and serum ferritin level > 500 - Eprex® dose increasing during the titration phase and a close iron monitoring. If serum ferritin is to decline rapidly, giving IV iron should be considered appropriately. For patients unable to access IV iron therapy, sponsor will support the iron therapy.

During last four weeks of the titration period, the Hb level will be evaluated to meet the inclusion criteria to conduct the randomization as same as observation period. Patients with stable Hb maintained between 10 and 12 g/dl and on stable Eprex® dose will proceed to enter the maintenance phase at the end of the titration period. The last four weeks of the titration phase will be considered the baseline period, and Hb levels measured during baseline period will be considered as baseline level.

3) <u>Maintenance Phase:</u>

The IV dose has to be adjusted individually to maintain hemoglobin between 10 - 12 g/dl. The hemoglobin, hematocrit, and dose will be monitored every two weeks during the maintenance phase. Investigators will decide to change or maintain the dosage of Eprex® or PDA10 every four weeks based on Hb level. The dosage will be determined according to the dose adjustment scheme below, and if hemoglobin level is not increased by more than 1 g/dl in any 2-week period or is not approaching 12 g/dl or below 10 g/dl, the dose will be maintained. The maintenance dose should be individualized, and the recommended total weekly dose should not exceed 300 IU/kg.

<u>Suggested Target Hemoglobin level Range:</u>

10 g/dl to 12 g/dl

During the study, Hb, Hematocrit and dose of Eprex® or PDA10 will be monitored every two weeks. Prior to and during the study, the patient's iron stores, including transferrin saturation (serum iron divided by iron binding capacity) and serum ferritin, will be evaluated.

<u>Dose Adjustment Scheme:</u>

If the hemoglobin increases by more than 1 g/dl in any 2-week period or the hemoglobin is approaching 12 g/d1, the dose should be reduced by approximately 25%. If the hemoglobin continues to increase or exceed 12 g/dl, the dose should be temporarily withheld until the hemoglobin begins to decrease, at which point therapy should be reinitiated at a dose approximately 25% below the previous dose. If dose reduction is needed, either the amount given per dose or the number of weekly injections will be reduced, or both will be reduced.

If the hemoglobin is below 10 g/dl, the dose may be increased by approximately 25% of the previous dose.

When a change in Eprex® or PDA10 dose is indicated, a one-step change (25% of current dosage) is performed according to the stepwise dosing schedule refer to appendix III.

The dose adjustment scheme in Open-Label Extension phase is same as maintenance phase, but hemoglobin will be assessed every 4 weeks.

10.4.5. Blinding

All PFSs will have the same external appearance and composition.

A list of subjects randomization codes will be generated by LSK global PS (an electronic version will be stored in a secure locked area). The list of patient randomization codes will be generated using PROC PLAN in the SAS® procedures. A stratified block randomization approach will be used for the randomization with stratification factor of country and study site. This list will identify each patient by randomization number and include the patient's corresponding treatment assignment. The randomization codes will be provided in a secure manner to the IWRS developer and the study drug packaging provider.

When the maintenance of the safety and well-being of the patient, as assessed by the Investigator, requires that the administered study treatment be revealed, the Investigator shall break the blind. Before a study drug is unblinded, every attempt should be made to discuss the case with the Sponsor to evaluate whether or not to break the blind. Unblinding will be done through the IWRS system. Accessing the IWRS for emergency unblinding should be done only in an emergency that necessitates identifying the study drug for the welfare of the patient.

An explanation for breaking the blind will be recorded in the relevant source documents. Breaking the code at the investigative study center will immediately disqualify the patient from further participation in the study.

10.4.6. Prior and Concomitant Therapy

10.4.6.1. Drug Interactions

No evidence of interaction of epoetin with other drugs was observed in the course of clinical trials. However, since cyclosporine is bound by RBCs there is potential for a drug interaction. If Eprex® is given concomitantly with cyclosporin, blood levels of cyclosporin should be monitored and the dose of cyclosporin adjusted as the hematocrit rises. An increase in heparin dose during hemodialysis is frequently required during the course of therapy with Eprex as a result of the increased packed cell volume. Occlusion of the dialysis system is possible if heparinazation is not optimum.

There is no rescue medication or procedure as this is not a placebo controlled trial.

10.4.7. Treatment Compliance

A monitor of the Sponsor (or the delegate) will review the pharmacy records at each center including the Drug Dispensing Record Form on which the pharmacist or delegated person should record all study drug released for patient use. The monitor will compare the dispensing record and PFSs to the individual subject's identifiers and visit schedule to assure that the subject receives the correct treatment, dose, and that the dosing schedule is correct. The monitor's report will include details of any missed doses, errors in dose, treatment errors or schedule errors and reasons for these. All supplies and pharmacy documentation must be made available throughout the trial for the monitor to review.

10.4.8. Expected Adverse Events and Precautions for Use

In larger overseas clinical studies of erythropoietin products conducted among patients with renal failure, a number of treatment-related side effects have occurred. Hypertension (24.7%) was the most commonly reported side effect, followed by muscle cramp, upper respiratory infection and headache, respectively reported in about 20% of subjects. Other frequently reported events were different types of infections, thrombosis (associated with hemodialysis in patients with renal failure), diarrhea, hypertension, dizziness, weakness, etc. whose incidence ranged 6~16%. Epileptic seizure was rarely reported at an incidence of 0.6%. In addition, a potential risk of infection with fatal pathogens such as

virus is suggested with the use of currently marketed erythropoietin products, which may be contaminated with animal-derived proteins during their cell culture process. Thus, the adverse events described above may occur during this clinical study. Based on the labeling information of Espogen Prefilled Syringe, a list of expected adverse events and precautions for use in this clinical study is prepared as follows:

10.4.8.1. Expected adverse events

Anaphylaxis: Rarely, anaphylactic shock may occur. A potential event should be confirmed with close monitoring and then appropriate measures such as treatment discontinuation should be taken.

1) Cardiovascular: Increased blood pressure, vascular access thrombosis, and occasionally, palpitation may occur.
2) Hypertensive encephalopathy: A sudden increase in blood pressure may lead to hypertensive encephalopathy, manifested by headache, disturbances of consciousness, seizure, etc. and further to cerebral hemorrhage. The investigational product should be administered under the close monitoring of blood pressure, hematocrit, etc.
3) Cerebral infarction: An event of potential cerebral infarction should be confirmed with close monitoring. If confirmed, appropriate measures such as treatment discontinuation should be taken.
4) Skin: Occasionally, itching, skin rash, etc. may occur.
5) Liver: Occasionally, hepatic dysfunction such as increases in AST, ALT, LDH, ALP, total bilirubin, etc. may be observed.
6) Gastrointestinal: Occasionally, nausea, vomiting, loss of appetite and diarrhea may occur. Abdominal cramp may also occur.
7) Blood: Occasionally, increased leukocyte and increased eosinophil may occur. A decreased granulocyte may sometimes be observed in preterm neonates. In occasional cases, serum potassium, BUN, creatinine and uric acid may increase. Rickets may sometimes develop in preterm infants.
8) Others: Bleeding inside the eyes, enlarged inner wall of nose, nose bleed, edema, and occasional events of headache, dizziness, fever, slight fever, hot flash, general malaise, arthralgia, myalgia, bitter taste, convulsion and periorbital edema may occur.
9) The investigational product is generally well tolerated. The adverse reactions reported are frequent sequelae of the underlying disease and are not necessarily attributable to the investigational product.

10.4.8.2. Contraindications

The administration of investigational product is contraindicated in patients:

1) with known hypersensitivity to any component of the investigational product
2) with uncontrolled hypertension
3) with known hypersensitivity to mammalian cell-derived products or human albumin products
4) who develop pure red cell aplasia (PRCA) following treatment with any erythropoietin product

10.4.8.3. Precautions for use

1) Hypertensive patients (Confirmed hypertensive episodes have been reported with the use of investigational product. Hypertensive encephalopathy may occur)
2) Patients with a history of drug hypersensitivity
3) Patients with a predisposition to allergy
4) Patients at a risk of thromboembolic event due to current or history of myocardial, pulmonary or cerebral infarction (An increase in blood thickness has been reported with the use of investigational product. Thromboembolism may develop newly or may be exacerbated. Especially, when the investigational product is used to facilitate autologous blood collection or in the post-operative period where clot-formation tends to increase, a close monitoring is required).

5) Preterm infants with intraventricular hemorrhage or intracranial hemorrhage (cerebral hemorrhage may be exacerbated with the use of investigational product).
6) Patients with ischemic vascular disease
7) Patients with a history of seizure
8) Cancer patients

10.5. STUDY PROCEDURES

10.5.1. Screening Assessments

10.5.1.1. Demographic information

Subject demography information will be collected at the Screening and includes date of birth (or age), sex, race/ethnicity, subject initial and duration and quantity of smoking and alcohol ingestion.

10.5.1.2. Medical and Surgical History

Medical and surgical history and current medical conditions (including anemia of chronic renal failure history) will be recorded at the Screening Visit. All pertinent medical and surgical history within 6 months (in case of malignancy; 5 years) must be noted in the Medical History and Current Medical Conditions in eCRF.

10.5.1.3. Prior Medication History

All medications the patient is taking at the time of study entry (Visit 1) and has taken within 6 months before entry must be recorded on the concomitant medication page in the eCRF. Thereafter, any changes in concomitant medications or new medications added will be recorded in the eCRF.

10.5.2. Efficacy Assessments

10.5.2.1. Hemoglobin Level

Hemoglobin level will be measured via hematologic examination from a blood sample, and will be recorded on a biweekly basis on eCRF. The target range of hemoglobin level is 10.0 to 12.0 g/dl.

10.5.2.2. Individual Dosage

Individual dosage will be recorded on a biweekly basis on eCRF, and doses must be individualized to ensure that hemoglobin is maintained within the target range for each subject.

10.5.2.3. Hematocrit Level

Hematocrit level will be measured via hematologic examination from a blood sample, and will be recorded on a biweekly basis on eCRF.

10.5.2.4. Blood Transfusion

During the study, incidence of blood transfusion will be compared between the two treatment groups.

10.5.3. Safety Asseements

The items below will be assessed and the results should be recorded in the followings: (1) source documents and (2) eCRFs. If any "new abnormal finding" considered clinically significant at the Investigator's discretion on any of the tests below or "aggravation" of the existing disease is observed after the subjects signs the informed consent form, an adequate treatment will be provided for the subject as needed. This case will be surveyed and assessed as an adverse event according to Section 9.5.3.3. Reporting Adverse Events. Any adverse event which did not disappear by the end of the study will be followed up.

10.5.3.1. Adverse Events

Any untoward medical occurrence in a patient or clinical investigation subject administered a pharmaceutical product and which does not necessarily have a causal relationship with this treatment. An adverse event (AE) can therefore be any unfavorable and unintended sign (including an abnormal laboratory finding), symptom, or disease temporally associated with the use of a medicinal (investigational) product, whether or not related to the medicinal (investigational) product (see the ICH Guideline for Clinical Safety Data Management: Definitions and Standards for Expedited Reporting).

In order to ensure complete safety data collection, all AEs or undesirable experiences occurring during the study (i.e., after signature of the Informed Consent), including any pre-and post-treatment periods required by the protocol, must be reported even if no investigational product was taken but specific study procedures were conducted.

Examples of AEs are as follows:

- Changes in the general condition of the subject
- Subjective symptoms offered by or elicited from the subject
- Objective signs observed by the Investigator or other study personnel
- All concurrent diseases that occur after the start of the study, including any change in severity or frequency of preexisting disease.
- All clinically relevant abnormalities in laboratory values or clinically relevant physical findings that occur during the study

Signs or symptoms of anemia should be recorded as AEs only if their nature changes considerably or their frequency or intensity increases in a clinically significant manner as compared to the clinical profile known to the Investigator from the subject's history or the baseline period (observation period)..

Adverse findings that are not considered clinically relevant based on medical judgment that are related to routine clinical laboratory evaluations, vital signs, or physical examinations should not be recorded on the AE reporting page of the eCRF. These findings should instead be recorded on the designated eCRF page.

<u>Causality Assessment</u>

For all AEs, the Investigator must provide an assessment of causal relationship to study drug. The causality assessment must be recorded on the appropriate AE reporting page of the subject's eCRF.

Not related: The relationship is unlikely or not related; i.e., there is no strong temporal relationship, and/or use of other drugs, underlying diseases, or other factors provide plausible explanations for the event or the subject did not take study drug.

Related: There is a possible or probable relationship; i.e., there is a reasonable or strong temporal relationship, and the events are unlikely attributed to other drugs, underlying diseases or other factors.

Severity Assessment

The Investigator will provide an assessment of the severity of each AE by recording a severity rating on the appropriate AE reporting page of the subject's eCRF. Severity is a description of the intensity of manifestation of the AE.

Severity will be assessed according to the following scale:

Mild: means awareness of symptoms or signs, but easily tolerated (acceptable).

Moderate: means enough discomfort to interfere with usual activity (disturbing).

Severe: means incapacity to work or to perform usual activities (unacceptable).

10.5.3.2. Serious Adverse Events

A Serious Adverse Events (SAE) is any untoward medical occurrence that occurs at any dose in any of the following outcome:

- results in death or is life-threatening
- requires inpatient hospitalization or prolongation of existing hospitalization
- results in persistent or significant disability/incapacity, or
- is a congenital anomaly/birth defect
- is medically important

In this context, the term life threatening refers to an event in which the subject was at immediate risk of death at the time of the event; it does NOT refer to an event which might have caused death if it would have been more severe.

Any important medical event that may not be immediately life threatening or result in death or hospitalization but, based upon appropriate medical judgment, may jeopardize the subject or may require medical or surgical intervention to prevent one of the other outcomes listed in the definition above should also be reported as a SAE. Examples of such events are intensive treatment in an emergency room or at home for allergic bronchospasm, blood dyscrasias or convulsions that do not result in hospitalization; or development of drug dependency or drug abuse.

Hospitalization for diagnostic or therapeutic procedures in the absence of any associated AE will not be considered as a SAE. If a hospitalization is planned prior to the subjects receiving the first dose of the investigational drug, it will not be classified as either an AE or SAE. This also applies to situation of scheduled elective surgery where no AE is present. Non-complicated, preplanned elective surgery

will not be considered an AE or SAE even if it involves hospitalization. However, if a hospitalization is unplanned or is a result of an AE, this will be considered an SAE.

Cases involving cancer as an Adverse Event could be reported as serious using the criterion “medically important”. Furthermore, emergency room visits that do not result in admission to the hospital will not be evaluated as SAEs.

Special Situation: Exposure to Study Drug during Pregnancy. Study personnel must report every pregnancy as soon as possible (within 24 hours after notification), even if no AE has occurred, and follow it to term. Investigators must instruct female subjects to inform them immediately should they become pregnant during the study. A subject who becomes pregnant during the study will immediately be discontinued (immediate stop of investigational product intake). The pregnancy will be registered in the Pregnancy Form to be completed with all available information and sent within the same time frame and following the same routing as for an SAE, as described below. The Investigator will make every effort to obtain all information related to the pregnancy and its final outcome; follow-up after the study has ended may be necessary.

All pregnancies that occurred during the study but only came to the knowledge of the Investigator after the termination of the study will be reported to the Sponsor or designee as if it occurred during the study. The same procedures specified above will be followed.

10.5.3.3. Reporting Adverse Events

The Sponsor (or the delegates) must be informed about an SAE within 24 hours of the site knowing about the event. The Investigator must promptly forward to the Sponsor (or the delegates) a duly completed Investigator SAE Report Form for Development Drug provided by the company, even if the data are incomplete or if it is obvious that more data will be needed to make any conclusions.

Additional information (e.g. autopsy or lab reports) should be provided to the Sponsor in a timely fashion to ensure accurate follow-up of each case.

The Sponsor (or the delegates) will communicate safety information and Suspected Unexpected Serous Adverse Reactions – SUSARs) to the appropriate agency (ies) and all active Investigators, in accordance with applicable regulatory requirements. The appropriate IRB/IEC will also be informed by the Investigator or the Sponsor, as specified by the applicable regulatory requirements in the Korea Food and Drug Administration (KFDA) or the appropriate agency (ies). Investigators are to retain evidence of such IRB/IEC notification in their study binder.

A copy of the Investigator SAE report form and the completion guide will be provided to the Investigator.

If known by the Investigator, Serious Adverse Events up to 30 days after the final study visit must be reported to the Sponsor, even if the Investigator is certain that they are in no way associated with the study drug.

Adverse Events that the Investigator thinks may be associated with the study medication must be reported to the Sponsor regardless of the time between the event and the end of the study.

Follow-Up Procedures for Serious Adverse Events

The Sponsor shall promptly investigate all safety information received by it. Follow-up information to a safety report shall be submitted as soon as the relevant information is available.

Relevant information such as discharge summaries, autopsy reports, and medical consultations shall be reviewed in detail by the Investigator. The Investigator shall comment on any event, abnormal laboratory result, or any other finding, noting whether it shall be considered a serious or nonserious AE or considered part of the subject's history. In addition, the Investigator shall report on an SAE form all subsequent events or other findings determined to be relevant and shall state for each event or finding whether it is related to study drug. All events determined to be nonserious shall be reported on the relevant eCRF page.

10.5.3.4. Clinical Laboratory Determinations

Samples for hematology and biochemistry assessments will be collected at every visit and the assessments will be conducted at the central laboratory or local laboratory. At Screening (Visit 1), the Investigator will assess the clinical significance of any values outside the reference ranges provided by the central laboratory; subjects with abnormalities judged to be clinically significant will be excluded from the study.
However, blood test for hemoglobin and hematocrit level can be conducted by the central laboratory. Biweekly basis data for hemoglobin and hematocrit level should be recorded on eCRF until the end of the Maintenance Phase.
HIV test will be conducted at Screening Visit (Visit 1), and CRP, PTH and iron state (Fe, ferritin, transferritin saturation) will be tested at Screening Visit (Visit 1), Baseline Visit (Visit 4), Week 12 (Visit 7), Week 28/Withdrawal (Visit 11), Week 40 (Visit 14) and Week 52/Withdrawal (Visit 17).

Hematology:	Hb, Hct, RBC, WBC, Plt, WBC diff count, PT/PTT
Serum biochemistry:	Na, K, Cl, total CO2, Ca, P, ALT/AST, Alkaline phosphatase, Total bilirubin, Glucose, Albumin, Total cholesterol, BUN, Serum creatinine, Uric acid
Iron evaluation:	Fe, ferritin, transferritin saturation

* Vitamin B12 and Folate evaluation will be conducted at Screening Visit (Visit 1).

10.5.3.5. Vital Signs

Vital sign measurements will be documented at every visit before administration of study medication. The parameters will include sitting pulse rate and sitting systolic and diastolic blood pressure. Pulse rate and blood pressure readings will be taken after the subject has been sitting for 5 minutes; the same arm should be consistently used for the measurements. Subjects found to have abnormal blood pressure will be evaluated by the PI to ensure to determine if they are eligible to continue in the study.

- Pulse
- Systolic/diastolic blood pressure

10.5.3.6. Chest X-rays

A plain postero-anterior chest X-ray should be taken unless a chest X-ray was performed and was clear within 3 months prior to Screening visit. The chest X-ray should be taken at Screening Visit (Visit 1), Baseline Visit (Visit 4), Week 28/Withdrawal (Visit 11) and Week 52 (Visit 17). However, chest X-ray result performed within 6 weeks prior to Baseline Visit can be used for baseline result.

10.5.3.7. Physical Examination

Physical examinations will be performed at every visit. The physical examination will include an examination of general appearance; eyes; ears; nose; throat; and the abdominal, skin/mucosa, lymphatic, cardiovascular, respiratory, neurological, and musculoskeletal systems.

10.5.3.8. Weight and Height

Weight (measured in kilograms) is measured at every visit and height (measured in centimeters), at the Screening Visit.

10.5.3.9. Pregnancy Test

For female subjects of childbearing potential, the Screening Visit (Visit 1), the Baseline Visit (Visit 4), Week 28/Withdrawal Visit (Visit 11) and Week 52/Withdrawal Visit (Visit 17) serum β-hCG pregnancy test results must be negative.

10.5.3.10. Concomitant medications

Should any treatment other than the investigational product be taken by the subject at screening or at any time during the course of the study (including over-the-counter products and nutraceuticals), an accurate record must be kept in the clinic chart (source documentation) and eCRF. This record should include the mane of the drug, the daily dose, the route and date(s) of administration, and the indication for use.

10.5.4. Immunological Assessment

10.5.4.1. Anti-epoetin antibody

1) Time of Blood Sampling

   The blood sampling to measure anti-epoetin antibodies will be conducted at Screening Visit (Visit 1), Baseline Visit (Visit 4), Week 28/Withdrawal Visit (Visit 11), and Week 52/Withdrawal Visit (Visit 17).

2) Measured Variables

   Blood samples for the determination of anti-epoetin antibodies will be sent from the site to the central antibody concentration analysis center for the clinical laboratory test.

10.5.5. Schedule of Procedures/Assessments

10.5.5.1. Schedule of Procedures/Assessments

Protocol Activity	Titration Phase							Maintenance Phase														Open-Label Extension Phase					
Visit Number[8]	1	1a	2	2a	3	3a	4	4a	5	5a	6	6a	7	7a	8	8a	9	9a	10	10a	11	12	13	14	15	16	17
Week Number	-12 Screening	-10	-8	-6	-4	-2	0 Baseline	2	4	6	8	10	12	14	16	18	20	22	24	26	28/W[7]	32	36	40	44	48	52/W[7]
Informed Consent[1]	X																										
Inclusion/Exclusion Criteria	X						X																				
Demographic Information	X																										
Medical and Surgical History	X																										
Prior Medication History	X																										
Anemia of Chronic Renal Failure History	X																										
Physical Examination	X		X		X		X		X		X		X		X		X		X		X	X	X	X	X	X	X
Vital Signs	X		X		X		X		X		X		X		X		X		X		X	X	X	X	X	X	X
Weight	X		X		X		X		X		X		X		X		X		X		X	X	X	X	X	X	X
Height	X																										
Hematology/Biochem[2]	X		X		X		X		X		X		X		X		X		X		X	X	X	X	X	X	X
Hb/Hct[2]		X		X		X		X		X		X		X		X		X		X							
CRP	X						X						X								X			X			X
Iron evaluation and PTH[2]	X						X						X								X			X			X
Vitamin B12 and Folate	X																										

Protocol Activity	Titration Phase							Maintenance Phase															Open-Label Extension Phase					
Visit Number[8)]	1	1a	2	2a	3	3a	4	4a	5	5a	6	6a	7	7a	8	8a	9	9a	10	10a	11	12	13	14	15	16	17
Week Number	-12 Screening	-10	-8	-6	-4	-2	0 Baseline	2	4	6	8	10	12	14	16	18	20	22	24	26	28/W[7)]	32	36	40	44	48	52/W[7)]
HIV Serum Testing	X																										
Chest X-ray[3)]	X						X														X						X
Anti-Epoetin Antibodies[4)]	X						X														X						X
Pregnancy Testing[5)]	X						X														X						X
Concomitant Medication	X		X		X		X		X		X		X		X		X		X		X	X	X	X	X	X	X
Adverse Events			X		X		X		X		X		X		X		X		X		X	X	X	X	X	X	X
PDA10 or Eprex® Administration[6)]	X		X		X		X		X		X		X		X		X		X		X	X	X	X	X	X	
Assessment of compliance			X		X		X		X		X		X		X		X		X		X	X	X	X	X	X	X

* W = withdrawal visit

1) If the subject is unable to provide written informed consent, written consent must be obtained from the subject's representative and verbal assent must be obtained from the subject.
2) If the laboratory values above exceed the upper limit of reference range, the re-test will be conducted by the investigator one more time to exclude the cases where the laboratory values continuously exceed the upper limit of reference range. Hemoglobin level and hematocrit level checked by the central laboratory will be recorded on a biweekly basis on eCRF until the end of the Maintenance Phase. Increases in dose should not be made more frequently than once 4 weeks. The clinical laboratory test, except hemoglobin and hematocrit will be conducted once four weeks.
3) Chest X-ray result within 3 months prior to Screening Visit will be available for screening. In addition, chest X-ray result performed within 6 weeks prior to Baseline Visit can be used for baseline result.
4) The blood sampling to measure anti-epoetin antibodies will be conducted at Screening Visit (Visit 1), Baseline Visit (Visit 4), Week 28/ Withdrawal Visit, and Week 52/ Withdrawal Visit.
5) Serum pregnancy test at Screening Visit, Baseline Visit, Week 28/ Withdrawal Visit and Week 52/Withdrawal Visit will be conducted.
6) Subjects will be administered Eprex® for the titration phase, PDA10 or Eprex® for the maintenance phase, and PDA10 for the open-label extension phase.

7) If subjects are withdrawn during the maintenance phase and open-label extension phase, the same procedure will be conducted with Visit 11 and Visit 17, respectively.
8) Visit window is ± 5 days. However, V4 blood sampling is allowed at -7 days from planned V4 date.

10.5.5.2. Description of Procedures/Assessments Schedule

Titration Phase

Visit 1 (Screening, Week -12)

Prior to any study activities, subjects will be asked to read and sign an informed consent form that has been approved by an IRB. Subjects will be given adequate time to consider any information concerning the study, given to them by the Investigator or designee.

Following assessments at this visit include:

- Signing informed consent form: The subject is given the oral explanation and written summarized exploratory statement about the study objectives, method, limitations and risks, study duration, etc. The subject signs and dates the consent form. One copy of the signed consent form is given to the subject.
- Confirm inclusion/exclusion criteria
- Demographic information
- Anemia of chronic renal failure history
- Significant past medical history (including allergies), concomitant disease, and drug history and concomitant drugs
- Vital signs (pulse and systolic and diastolic BP)
- Height and weight
- Blood sample for clinical laboratory values (Hematology/Serum biochemistry, CRP, PTH, Iron evaluation, Vitamin B12 and Folate, HIV, Anti-erythropoietin antibody, Serum pregnancy test)
- Physical examination
- Chest X-ray

Only patients who meet all inclusion/exclusion will be allowed to continue the remaining steps and administer Eprex®. If not, terminate the patient from the study.

Visit 1a (Week -10)

Clinical laboratory test for Hb and Hct will be performed.

Visit 2 (Week -8)

The following procedures will be conducted at this visit:

- Physical examination
- Vital signs (pulse and systolic and diastolic BP)

- Weight
- Blood sample for clinical laboratory values (Hematology/Serum biochemistry)
- Concomitant drugs
- Adverse events
- Eprex® administration
- Assessment of compliance

Visit 2a (Week -6)

Clinical laboratory test for Hb and Hct will be performed.

Visit 3 (Week -4)

The following procedures will be conducted at this visit:

- Physical examination
- Vital signs (pulse and systolic and diastolic BP)
- Weight
- Blood sample for clinical laboratory values (Hematology/Serum biochemistry)
- Concomitant drugs
- Adverse events
- Eprex® administration
- Assessment of compliance

Note: The duration of the titration phase is 12 weeks, and a prolongation of the titration phase will be allowed up to 20 weeks in substantiated cases. If a subject need a prolongation of the titration phase after Visit 3, the next visit will be Visit 3-1 (4 extra weeks of titration phase) and Visit 3-2 (8 extra weeks of titration phase), and the same procedures will be conducted with Visit 3.

Visit 3a (Week -2)

Clinical laboratory test for Hb and Hct will be performed.

Maintenance Phase

Visit 4 and Randomization (Baseline, Week 0)

Subjects with hemoglobin level within the target range between 10.0 and 12.0 g/dl will have the following procedures performed/recorded prior to study drug administration:

Review of inclusion/exclusion criteria

The following steps must be followed for the subjects to qualify for randomization:

- Physical examination
- Vital signs (pulse and systolic and diastolic BP)
- Weight
- Blood sample for clinical laboratory values (Hematology/Serum biochemistry, CRP, PTH, Iron evaluation, Anti-erythropoietin antibody, Serum pregnancy test)
- Chest X-ray (the result performed within 6 weeks prior to Baseline Visit can be used)
- Concomitant drugs
- Adverse events
- PDA10 or Eprex® administration **(Post randomization)**
- Assessment of compliance

Visit 4a (Week 2)

Clinical laboratory test for Hb and Hct will be performed.

Visit 5 (Week 4)

The following procedures will be conducted at this visit:

- Physical examination
- Vital signs (pulse and systolic and diastolic BP)
- Weight
- Blood sample for clinical laboratory values (Hematology/Serum biochemistry)
- Concomitant drugs
- Adverse events
- PDA10 or Eprex® administration
- Assessment of compliance

Visit 5a (Week 6)

Clinical laboratory test for Hb and Hct will be performed.

Visit 6 (Week 8)

The following procedures will be conducted at this visit:

- Physical examination
- Vital signs (pulse and systolic and diastolic BP)
- Weight
- Blood sample for clinical laboratory values (Hematology/Serum biochemistry)
- Concomitant drugs
- Adverse events
- PDA10 or Eprex® administration
- Assessment of compliance

Visit 6a (Week 10)

Clinical laboratory test for Hb and Hct will be performed.

Visit 7 (Week 12)

The following procedures will be conducted at this visit:

- Physical examination
- Vital signs (pulse and systolic and diastolic BP)
- Weight
- Blood sample for clinical laboratory values (Hematology/Serum biochemistry, CRP, PTH, Iron evaluation)
- Concomitant drugs
- Adverse events
- PDA10 or Eprex® administration
- Assessment of compliance

Visit 7a (Week 14)

Clinical laboratory test for Hb and Hct will be performed.

Visit 8 (Week 16)

The following procedures will be conducted at this visit:

- Physical examination
- Vital signs (pulse and systolic and diastolic BP)
- Weight
- Blood sample for clinical laboratory values (Hematology/Serum biochemistry)
- Concomitant drugs
- Adverse events
- PDA10 or Eprex® administration
- Assessment of compliance

Visit 8a (Week 18)

Clinical laboratory test for Hb and Hct will be performed.

Visit 9 (Week 20)

The following procedures will be conducted at this visit:

- Physical examination
- Vital signs (pulse and systolic and diastolic BP)
- Weight
- Blood sample for clinical laboratory values (Hematology/Serum biochemistry)
- Concomitant drugs
- Adverse events
- PDA10 or Eprex® administration
- Assessment of compliance

Visit 9a (Week 22)

Clinical laboratory test for Hb and Hct will be performed.

Visit 10 (Week 24)

The following procedures will be conducted at this visit:

- Physical examination
- Vital signs (pulse and systolic and diastolic BP)
- Weight
- Blood sample for clinical laboratory values (Hematology/Serum biochemistry)
- Concomitant drugs
- Adverse events
- PDA10 or Eprex® administration
- Assessment of compliance

Visit 10a (Week 26)

Clinical laboratory test for Hb and Hct will be performed.

Visit 11 (Week 28/Withdrawal)

The following procedures will be conducted at this visit:

- Physical examination
- Vital signs (pulse and systolic and diastolic BP)
- Weight
- Blood sample for clinical laboratory values (Hematology/Serum biochemistry, CRP, PTH, Iron evaluation, Anti-erythropoietin antibody, Serum pregnancy test)
- Chest X-ray
- Concomitant drugs
- Adverse events
- PDA10 administration (subjects who participate in the OLE phase only)
- Assessment of compliance

* Visit 11 at Week 28 will serve as the entry visit for the open label extension phase.

Subjects who signed the ICF will perform the next visit.

Subject who will NOT be participated in the extension phase will be returned to the standard treatment provided at the hospital/clinic respectively.

Visit 12 (Week 32)

The following procedures will be conducted at this visit:

- Physical examination
- Vital signs (pulse and systolic and diastolic BP)
- Weight
- Blood sample for clinical laboratory values (Hematology/Serum biochemistry)
- Concomitant drugs
- Adverse events
- PDA10 administration
- Assessment of compliance

Visit 13 (Week 36)

The following procedures will be conducted at this visit:

- Physical examination
- Vital signs (pulse and systolic and diastolic BP)
- Weight
- Blood sample for clinical laboratory values (Hematology/Serum biochemistry)
- Concomitant drugs
- Adverse events
- PDA10 administration
- Assessment of compliance

Visit 14 (Week 40)

The following procedures will be conducted at this visit:

- Physical examination
- Vital signs (pulse and systolic and diastolic BP)
- Weight
- Blood sample for clinical laboratory values (Hematology/Serum biochemistry, CRP, PTH, Iron evaluation)

- Concomitant drugs
- Adverse events
- PDA10 administration
- Assessment of compliance

Visit 15 (Week 44)

The following procedures will be conducted at this visit:

- Physical examination
- Vital signs (pulse and systolic and diastolic BP)
- Weight
- Blood sample for clinical laboratory values (Hematology/Serum biochemistry)
- Concomitant drugs
- Adverse events
- PDA10 administration
- Assessment of compliance

Visit 16 (Week 48)

The following procedures will be conducted at this visit:

- Physical examination
- Vital signs (pulse and systolic and diastolic BP)
- Weight
- Blood sample for clinical laboratory values (Hematology/Serum biochemistry)
- Concomitant drugs
- Adverse events
- PDA10 administration
- Assessment of compliance

Visit 17/Withdrawal (Week 52)

The following procedures will be conducted at this visit:

- Physical examination
- Vital signs (pulse and systolic and diastolic BP)
- Weight
- Blood sample for clinical laboratory values (Hematology/Serum biochemistry, CRP, PTH, Iron evaluation, Anti-erythropoietin antibody, Serum pregnancy test)
- Chest X-ray
- Concomitant drugs
- Adverse events
- Assessment of compliance

Unscheduled Visit

With regard to adverse events, change in concomitant drugs, withdrawals and the cases requiring medical treatment as a result of the clinical tests, if the subject visits on the day which is not scheduled, the corresponding matters should be recorded in the relative form. The visit schedule should not be changed due to the unscheduled visit.

Subject Completion

Subjects will be asked to participate in the open-label extension (OLE) phase at Visit 1. Among them, subjects who complete the Week 28 will be offered PDA10 treatment.

After end of open-label extension phase, subject to return to the standard treatment provided at the hospital/clinic respectively.

Handling of Biological Samples

Central laboratory will provide with a laboratory manual which will explain detailed descriptions of collection, preparation and labeling/shipping requirements for biological samples to each site.

10.6. STATISTICAL METHODS

10.6.1. General Principles of the Result Analysis

Statistical analyses will be performed using the data analysis software SAS® Version 9.2 or higher.

Statistical testing will be done at alpha=0.05 level, two-sided, without any adjustment for multiple comparisons unless otherwise specified. The p-values will be rounded to 4 decimal places; p-values less than 0.0001 will be presented as "<.0001" in all tables.

Summaries of categorical variables (norminal or ordinal) will include frequency and percentage of patients at each level of response. Continuous variable summaries will include n, mean, median, standard deviation, minimum and maximum values.

The primary efficacy analysis will be based on the PP population.

10.6.2. Study Populations

10.6.2.1. Safety Population

The Safety Population consists of all patients who started therapy with randomized study medication.

10.6.2.2. Full Analysis Set (FAS) Population

The Full Analysis Set (FAS) Population consists of all patients who were treated more than 4 weeks with randomized study medication and had at least one postbaseline value of the primary endpoints.

10.6.2.3. Per-Protocol Population

The Per-Protocol Population, a subset of the FAS Population, is defined as all patients who completed the maintenance phase without any major protocol violations. The precise reasons for excluding patients from the Per-Protocol Population will be fully defined and documented before unblinding.

10.6.3. Demographics and Clinical History Data

Demographic variables and other baseline characteristics will be summarized by treatment group for the FAS Populations. Summaries of categorical variables (norminal or ordinal) will include frequency and percentage of patients at each level of response.

For continuous variables, the number of observations, mean, median, standard deviation, minimum, and maximum will be presented.

Treatment difference will be analyzed using two sample t-test or Wilcoxon rank-sum test for continuous parameters, and using χ^2-test or Fisher's exact test for categorical parameters.

10.6.4. Efficacy Assessment

10.6.4.1. Primary Efficacy Variable

Maintenance Phase

From the statistical point of view, the question of therapeutic equivalence will be approached by calculating the upper limit and the lower limit of 95% one-sided confidence interval of the difference between both treatment groups of the primary endpoints:

- mean change in hemoglobin level between the baseline period and the evaluation period
- mean change in weekly dosage per kg body weight between the baseline period and the evaluation period

* The baseline period is the last four weeks of titration phase and the evaluation period is the last four weeks of maintenance phase. Hemoglobin, hematocrit and the dosage of epoetin for the baseline period will be collected at Week -4, Week -2 and Week 0, and those for the evaluation period will be collected at Week 24, Week 26 and Week 28.

To demonstrate the therapeutic equivalence, two one-sided test (TOST) procedures will be used. It decomposes the interval hypothesis into two one-sided parts – H_0: $d < d_L$ and H_1: $d > d_U$, where d is the difference of the mean change in each primary endpoint, d_L is the lower limit of 95% one-sided confidence interval and d_U is the upper limit of 95% one-sided confidence interval. Both of these one-sided intervals will be compared with the pre-defined clinical acceptance ranges for the corresponding parameters (± 0.5 g/dl for hemoglobin and ± 45 IU/kg/week for epoetin dosage, based on the respective reference means). The intervals will be calculated by means of ANCOVA model including treatment group as a factor and study center and baseline value as covariates.

The statistical evaluation of the primary endpoint “mean change in weekly dosage per kg body weight between the baseline period and the evaluation period” will be performed based on the nominal dosage declared on the labels of the pre-filled syringes.

As the dosage of epoetin and the corresponding level of hemoglobin are closely interrelated, a hierarchic test strategy will be used in the present trial. The test on a higher level of hierarchy can only be performed, should the target of the previous level be fulfilled. The overall equivalence statement is consistent with a positive outcome on both levels of hierarchy. Due to this reason, no adjustment of alpha values will be required on the separate levels. The levels of hierarchy will be defined as follows:

Level 1: Calculation of the upper limit and the lower limit of 95% one-sided confidence interval of the difference (test - reference) of the mean change in hemoglobin level between the baseline period and the evaluation period and comparison with the pre-defined acceptance range.

Level 2: Calculation of the upper limit and the lower limit of 95% one-sided confidence interval of the difference (test - reference) of the mean change in weekly dosage per kg body weight between the baseline period and the evaluation period and comparison with the pre-defined acceptance range.

Open-label Extension Phase

The statistical analysis of the results of the open follow-up extension period will be only descriptive. Depending on their distribution the target parameters will be presented with their mean, median, standard deviation and quartiles or with their incidences.

10.6.4.2. Secondary Efficacy Variables

Summaries of categorical variables (norminal or ordinal) will include frequency and percentage of patients at each level of response. Continuous variable summaries will include n, mean, median, standard deviation, minimum and maximum values.

Treatment difference will be analyzed using two sample t-test or Wilcoxon rank-sum test for continuous parameters, and using χ^2-test or Fisher's exact test for categorical parameters.

10.6.5. Safety Assessment

10.6.5.1. Extent of Exposure

Exposure to study drug for the Safety Population of the maintenance phase will be summarized for treatment duration, calculated as the number of days from the date of first randomized study medication taken to the date of last dose taken, inclusive. Descriptive statistics (n, mean, standard deviation, median, minimum, and maximum) will be presented by treatment group.

10.6.5.2. Adverse Events

An AE that occurs during the treatment period will be considered a TEAE if it was not present prior to the first dose of study drug or was present prior to the first dose of study drug but increased in severity during the treatment period. If the same AE is reported more than once prior to the first dose of study drug, the AE with the greatest severity will be used as the benchmark for comparison with the AEs occurring after the first dose of study drug that were coded to the preferred term.

All AEs will be classified by System Organ Class (SOC) and Preferred Term (PT) according to Medical Dictionary for Regulatory Activities (MedDRA). If more than one AE is coded to the same preferred term for the same patient, the patient will be counted only once for that preferred term using the most severe and most related occurrence for the summarization by severity and by relationship to the study drug.

The number, percentage and the corresponding 95% CI of patients experiencing TEAEs in each treatment group will be presented. The significance test for treatment difference will be carried out using χ^2-test or Fisher's exact test.

Listings will be presented for patients with SAEs, patients with AEs leading to permanent discontinuation, and patients with AEs leading to death.

10.6.5.3. Vital Signs

Descriptive statistics for vital signs and changes from baseline at each visit will be presented by treatment group.

A supportive listing of patients with clinically significant abnormal postbaseline values will be provided, including the Patient ID, study center, and baseline and postbaseline values.

10.6.5.4. Physical Examination

A listing of any new (or worsening) findings will be provided.

10.6.5.5. Clinical Laboratory Test

Descriptive statistics for clinical laboratory values and changes from the baseline values at each visit will be presented by treatment group for each clinical laboratory parameter. Also, the shift-table of change of status from the baseline to the last visit will be presented for each clinical laboratory parameter. Listings will be presented for patients with clinically significant abnormal values.

10.6.5.6. Chest X-ray

Listings will be presented for patients with chest x-ray abnormalities during the trial.

10.6.6. Immunology

10.6.6.1. Anti-Epoetin Antibody

Listings will be presented for patients with anti-epoetin antibodies during the trial.

10.6.7. Determination of the Sample Size and it's Rationale

10.6.7.1. Sample Size

There are two co-primary endpoints in this study; the mean change in hemoglobin level and the mean change in weekly dose per kg body weight from baseline period to the evaluation period. In determining the sample size of studies with co-primary endpoints, generally sample sizes are calculated for each co-primary endpoint and then the larger sample size is adopted (Volker Wizemann et al, 2008).

To achieve 90% power of the mean change in hemoglobin level, a sample size of 126 is required per group. In the case of weekly dose, a sample size of 97 is needed per group. The larger sample size of hemoglobin level (n=126) is chosen for the trial, which is expected to give a overall 80% power for the proof of equivalence. The total number of patients to be enrolled will be 316, including an assumed drop-out/protocol violation rate of 20%.

10.6.7.2. Rationale

The primary objective is to demonstrate the therapeutic equivalence of PDA10 to Eprex in patients with end stage renal failure (ESRD) on chronic hemodialysis. The hypotheses of interest are given by

$$H_0 : |\theta_t - \theta_c| \geq \delta \text{ versus } H_1 : |\theta_t - \theta_c| < \delta,$$

where θ_t = Mean change in hemoglobin level/weekly dose between the baseline period and the evaluation period in the PDA10 group,

θ_c = Mean change in hemoglobin level/weekly dose between the baseline period and the evaluation period in the Eprex group,

and δ = Equivalence margin

It is assumed that the mean change in hemoglobin level/weekly dose for PDA10 group and Eprex group are equal and the standard deviations of the mean change in hemoglobin level and weekly dose are also assumed to be 1.1 g/dL (M. Haag-Weber et al. (2007)) and 86.6 IU/kg (Krivoshiev S. et al. (2010)), respectively (Refer to the table below).

Reference	Treatment	Endpoint	Difference	95% CI	Inferred SD
M. Haag-Weber et al. Scientific discussion EMEA:1-26, 2007	HX575 (N=207)	Mean Hb change	0.08	(-0.17, 0.34)	1.1
	Eprex (N=118)				
Krivoshiev S. et al. Advances in Therapy 2010	Eepoetin Zeta (N=154)	Mean weekly epoetin dosage over last 4 wks	11.0	(-8.06,29.96)	86.6
	Eprex (N=165)				

In M. Haag-Weber et al. (2007), the equivalence margin of hemoglobin level was ±0.5 g/dL and "Guideline of Preclinical and clinical evaluation for Biosimilar in Erythropoietin" by KFDA also recommends that the appropriate equivalence margin is ±0.5 g/dL. Therefore equivalence margin of hemoglobin is defined ±0.5 g/dL. And the equivalence margin of weekly dose is defined ±45 IU/kg (S. Krivoshiev et al. (2010) and V. Wizemann et al. (2008)).

The subjects are randomly allocated to either PDA10 or Eprex group in the ratio of 1:1. The sample size resulting from hemoglobin level and weekly dose for achieving a 90% power with 5% significance level is given by

[Hemoglobin level]

$$n_t = \frac{(1+1/k)(z_\alpha + z_{\beta/2})^2\sigma^2}{(\delta - |\varepsilon|)^2} = \frac{2\times(1.645+1.96)^2\times 1.1^2}{0.5^2} = 125.80 \fallingdotseq 126 \text{ for the treatment group,}$$

[Weekly dose]

$$n_t = \frac{(1+1/k)(z_\alpha + z_{\beta/2})^2\sigma^2}{(\delta - |\varepsilon|)^2} = \frac{2\times(1.645+1.96)^2\times 86.6^2}{45^2} = 96.26 \fallingdotseq 97 \text{ for the treatment group,}$$

where $\varepsilon = \theta_t - \theta_c = 0$ (mean difference between PDA10 group and Eprex group),

$k = n_c / n_t = 1$ (allocation ratio),

and $n_c = kn_t$ (the number of patients in the control group)

Consequently, the planned sample size to show the therapeutic equivalence between PDA10 and Eprex group is 126 patients per group. Considering that the chance to be excluded from the PP population is 20%, the total number of patients to be enrolled will be 316 (158 per group).

10.6.8. Handling of Dropouts of Missing Data

- Efficacy analysis:

Any missing data due to subject withdrawal during the study is imputed using the LOCF method. Baseline data is not carried forward to impute the missing data.

- Safety analysis:

Safety analysis is performed on observed value only.

10.6.9. Interim Analysis

The first clinical study report (CSR) will be reported to regulatory agency following completion of the maintenance phase, and the additional CSR will be reported following completion of the open label extension phase.

10.7. CHANGES IN THE CONDUCT OF THE STUDY OR PLANNED ANALYSES

10.7.1. Changes in the Conduct of the Study

Any amendment to this protocol will be provided to the Investigator in writing by the Sponsor. No protocol amendment may be implemented (with the exceptions noted below) before it has been approved by the IRB/IEC and the signature page, signed by the Investigator, has been received by the Sponsor. If the protocol is amended to eliminate or reduce the risk to subjects, the amendment may be implemented before IRB/IEC review and approval. However, the IRB/EC must be informed in writing of such an amendment, and approval must be obtained within reasonable time limits. Deviating from the protocol is permitted only if absolutely necessary for the safety or clinical management of the subjects and must immediately be reported to the Sponsor.

10.7.2. Planned period of the Study

This study will be conducted for about 24 months after the IRB approval.

11.QUALITY CONTROL AND QUALITY ASSURANCE

11.1. MONITORING

The Sponsor of this study is will be responsible for the health authorities for making the study conducted in an adequate way by taking all the measures required to comply with ethics, and the protocol and guarantee the unity and validity of the data recorded in the eCRF. Therefore, the major duty of the monitoring team is to help maintain high ethical, scientific, technical and legal quality in all aspects. Moreover, they should confirm whether the study is conducting according to the protocol, ICH GCP Guidelines and all applicable regulatory requirements.

At the time of monitoring of this study, whether the case report form is complete and clear should be confirmed, the case report form should be compared with the source data in the presence of the Investigator and the Investigator should cooperate with the sponsor at any time. During the monitoring visit, the following matters should be examined with the Investigator, informed consent form, subject recruitment and follow-up, serious adverse events documentation and reporting, dispensation of investigational products, subject's compliance with administration and dosage of investigational products, quantity of investigational products, concomitant treatment and quality of data.

Monitoring of the study is the responsibility of the Sponsor and may be delegated to the Sponsor's delegate (a CRO or a contract monitor). The Monitor (the individual responsible for monitoring) will advise the Investigator regarding the practical conduct of the study and maintaining compliance with the protocol, GCP, and all applicable regulatory requirements.

The Investigator will allow the Sponsor or its representatives to review periodically, at mutually convenient times during the study and after the study has been completed, all CRFs and corresponding source documents (e.g., portions of office, hospital and laboratory records for each study participant). Therefore, the monitor will have direct access to these records. The monitoring visits provide the sponsor or its representatives with the opportunity to evaluate the progress of the study, to verify the accuracy and completeness of eCRFs, to ensure that all protocol requirements, applicable authorities' regulations and Investigator's obligations are being fulfilled, and to resolve any inconsistencies in the study records.

If the monitoring and/or auditing process identifies serious and/or persistent non-compliance by an Investigator/institution, the Sponsor may terminate the Investigator's/institution's participation in the study.

11.2. AUDIT AND INSPECTION

The Sponsor or Sponsor's delegate may conduct audit as a part of quality assurance and confirm whether the study was conducted according the protocol, SOP and related regulations. And the Commissioner of KFDA or other regulatory agency may confirm whether the study was conducted according the KGCP and related regulations through inspection.

12.PUBLICATION AND PRESENTATION

By signing this protocol, the Investigator consents to use the results of this study for the purpose of registration, publication and provision of information for medical and pharmaceutical professionals. Before publishing the results of the study in a medical journal or academic journal, the Sponsor has the right to examine the published information. The Investigator must not publish, present or disclose information related to the results of the study without the Sponsor’s prior written consent.

The Sponsor has the right to publish the results of the study at any time.

All information obtained from the trial subjects during this study will not be disclosed to anyone else and will be kept confidential. Data of the study results will be investigated only for medical purposes. If the study results are published as necessary, the subject’s identity will remain confidential.

In addition, subjects have no access to study data and the results of this study.

13.INTELLECTUAL PROPERTIES

All the information, documents and investigational products provided by the Sponsor (or the delegate) are the property of the Sponsor. The Investigator cannot mention any information or product related to patent or any other intellectual property applications.

All the results, data, documents and findings obtained from the study directly and indirectly in any way exclusively belong to the properties, the Sponsor may use or publish all the results without any limitations at the Sponsor’s discretion. The Sponsor does not have duties with regard to patent, development, sales or use of the study results.

Depending on the situation, the Investigator will provide all the supports required by the Sponsor to obtain or protect any patents including signatures on the legal documents while the Sponsor pays costs.

14.DATA QUALITY ASSURANCE

14.1. DATA MONITORING

Before any subject enters the study, a representative of the Sponsor or the delegate will meet with the Investigator and his/her staff to review the procedures to be followed while conducting the study and recording the findings in the eCRFs, and to train them on recording the data on eCRFs using the electronic data capture (EDC) system. After the first patient is enrolled, the Sponsor’s representative or the delegate will periodically monitor the progress of the study by conducting on-site visits, and will also be able to review query statuses remotely, possibly warranting more frequent communication with the Investigator and his/her staff. The Investigator will make available to the Sponsor’s representative or the delegate eCRFs and the computer for review, source documents, signed consent forms, and all other study-related documents. The Investigator will be responsible for reviewing eCRFs, signing the appropriate eCRF page(s), resolving data queries generated via the system, approving all changes performed on his/her data, and endorsing the patient data within the EDC system. This approval method will include applying an electronic signature, a uniquely assigned username and password that together will represent a traditional handwritten signature. A copy (archive) of each eCRF will be retained by the Investigator.

14.2. DATA RECORDING AND DOCUMENTATION

Data collection will involve the use of the EDC system, to which only authorized personnel will have access. In addition to periodic monitoring occurring within the system by the Sponsor, programmatic

edit checks will be used to review the data for completeness, logic, and adherence to study protocol. As a result of this monitoring and these checks, queries may be electronically issued to the clinical study centers and electronically closed by those study centers. The identifying information (assigned username, date, and time) for both the originator of the query (if created during the monitoring process) and the originator of the data change (if applicable), as well as the PI's approval of all changes performed on his/her patients' data, will be collected.

All data collected in the context of this study will be stored and evaluated per regulatory requirements and applicable guidance for electronic records. Also, data will be stored and evaluated in such a way as to guarantee patient confidentiality in accordance with the legal stipulations applying to confidentiality of data. Study records (i.e., copies of eCRFs, regulatory documents) will be retained at the study center or the 3rd parties for 15 years from the study ends, along with adequate source documentation, according to KFDA or other regulatory agency and ICH requirements. After period of storage, the study records will be destroyed according to the regulatory guidelines. All study records must be available for inspection by the Sponsor, its authorized representatives, and the KFDA or other regulatory agency.

15.SUBJECT CONFIDENTIALITY

The names of all the subjects will be kept confidential. The subjects will be identified with the number given during the study and initials for records and evaluations. All the study data on the subject will be strictly handled as confidentiality. The original copy of the signed informed consent form should be retained by the Investigator. The Investigator should confirm the record and management of the list where the numbers and names of the subjects were recorded.

The Principal Investigator should limit the information provided by the Sponsor as confidential and retain the provided documents (the protocol, Investigator's Brochure, eCRF and other documents) in a place where an adequate confidentiality is guaranteed. The information provided for the investigator by the Sponsor should not be disclosed to the third person without prior written consent of the Sponsor except to obtain the consent form from the subject who wants to participate in the study.

It is allowed to submit this protocol and other required documents to the IRB and the IRB members should also guarantee the same confidentiality.

16. MEDICAL CARE AND TREATMENT FOLLOWING STUDY COMPLETION

For any adverse event considered needing treatment by the subinvestigator, appropriate diagnostic procedures and treatments will be applied, and it will be conducted that the medical treatment considered as the best option for the patients who complete the study.

17.DUTIES

17.1. INVESTGATOR'S DUTIES

During the study, the Principal Investigator and Investigators should make effort for the subject safety. If serious adverse events occur, they should take prompt and appropriate measures to provide the subject with the necessary tests and treatment in order to minimize the adverse events.

The Principal Investigator will educate the Investigators and subjects or their guardians on all the adverse events which can occur after the investigational product administration and ask them to report all the events.

The amendments or changes in the protocol must be submitted to the IRB. The events which may influence the subject or continuation of the study, especially changes related to safety must be reported. The revised Investigator's brochure should be submitted to the IRB.

If there is a request, the report on the process will be submitted to the IRB every year and the summary of the study results will be submitted to the IRB at the completion of the study.

17.2. SPONSOR'S DUTIES

The Sponsor or the delegate is responsible for reporting the safety information obtained from the study to the KFDA or other regulatory agency in accordance to KGCP and related regulations.

In relation to the report above, the Sponsor should report additional safety data regularly until the adverse event in question is terminated (disappearance or impossible follow-up of the adverse drug reaction in question).The Sponsor should report all the safety information found during the study in the clinical study report.

18.REFERENCES

- Astor BC, Muntner P, Levin A, et al. Association of kidney function with anemia: the Third National Health and Nutrition Examination Survey (1988-1994). Arch Intern Med. 2002; 162(12): 1401–8.
- Bommer J, Alexiou U, luller-Buhl E, Eifert J, Ritz E. Recombinant human erythropoietin therapy in haemodialvsis patients-dose determination and clinical experience. Nephrol Dial Transplant 1987;2:238-42.
- Casati S, Passerini .P, Campise MR, et al. Benefits and risks of protracted treatment with human recombinant erythropoietin in patients having haemodialysis. Br Aledj 1987;295: 1017-20.
- Eckardt KU. Pathophysiology of renal anemia. Clin Nephrol. 2000; 53(1 Suppl.):S2–8.
- Eschbach JW, Abdulhadi MH, Browne JK et al. Recombinant human erythropoietin in anemic patients with end-stage renal disease. Results of a phase III multicenter clinical trial. Ann Intern Med 1989; 111: 992–1000
- Eschbach JW, Adamson JW. Recombinant human erythropoietin: implications for nephrology. Am] Kidney Dis 1988;11:203-9.
- Eschbach JW', Downing MR, Egrie JC, Browne JK, Adamson JW. USA multicenter clinical trial with recombinant human erythropoietin (Amgen). Contrib Nephrol 1989;76:160-5.
- Eschbach JW, Egrie JC, Downing MR, Browne JK, Adamson JW. Correction of the anemia of end-stage renal disease with recombinant human erythropoietin. N Englj Med 1987;316:73-8.
- Eschbach JW, Kelly MR, Halev NR, Abels RI, Adamson JW. Treattnent of' the anemia of progressise renal failure with recombinant human crvthropoietin. N Engl]f Med 1989;321:158-63.
- Eschbach JW. The anaemia of chronic renal failure: pathophysiology and the effects of recombinant erythropoietin. Kidney Int 1989;35:134-48.
- Frenken LAM, Coppens PJW, Tiggeler RGWL, Koene RAP. Intraperitoneal erythropoietin. Lancet 1988;ii:1495.
- Gertz B, Kohler E, Kes P, Essaian A, Bias P, Buchner A, Elsässer R, Epoetin theta: efficacy and safety of IV administration in anaemic haemodialysis patients in the maintenance phase in comparison to epoetin beta, Curr Med Res Opin. 2010 Oct;26(10):2393-402
- Haag-Weber M, Vetter A, Thyroff-Friesinger U, Therapeutic equivalence, long-term efficacy and safety of HX575 in the treatment of anemia in chronic renal failure patients receiving hemodialysis. Clin Nephrol. 2009 Nov; 72(5):380-90
- lain C Macdougall, R David Hutton, Ivor Cavill, Gerald A Coles, John D Williams. Treating renal anaemia with recombinant human erythropoietin: practical guidelines and a clinical algorithm. BMJ 1990;300:655-9
- Lefebvre P, Vekeman F, Sarokhan B et al. Relationship between hemoglobin level and quality of life in anemic patients with chronic kidney disease receiving epoetin alfa. Curr Med Res Opin 2006; 22: 1929–1937
- Levin A. Prevalence of cardiovascular damage in early renal disease. Nephrol Dial Transplant. 2001; 16(Suppl. 2):7–11.
- Lim VS, DeGowin RL, Zavaba D, et al. Recombinant human erythropoietin treatment in pre-dialysis patients: a double-blind placebo-controlled trial. Ann InternMcled 1989;110:108-14.
- Macdougall IC, Lewis NP, Saunders MJ, et al. Exercise capacity, fistula blood flow, and rheological studies during treatment with rHuElPO in haemodialysis patients. Nephrol Dial 7ransplant 1989;4:319.
- Macdougall IC, Tucker B, Thompson J, Tomson CR, Baker LR, Raine AE (1996). "A randomized controlled study of iron supplementation in patients treated with erythropoietin". Kidney Int. 50 (5): 1694–9.
- Mayer G, Steffenelli r, 'I'hum J, Cada EM, Stummvoll HK, Graf H. Haemodynamic parameters and blood viscosity in the pathogenesis of ervthropoietin treatment related h pertension. Nephrol Dial 7ransplant 1988 ;3:499.
- Mayer G, Thum J, Cada EM, Stummvoll HK, Graf H. Working capacity is increased following recombinant human erythropoietin treatment. Kidney Int 1988;34:525-8.
- Nangaku M, Eckardt KU. Pathogenesis of renal anemia. Semin Nephrol. 2006; 26(4):261–8.

- National Kidney Foundation (2002). "K/DOQI clinical practice guidelines for chronic kidney disease". http://www.kidney.org/professionals/KDOQI/guidelines_ckd. Retrieved 2008-06-29
- Schaefer RM, Horl WH, Massry SG. Treatment of renal anemia with recombinant human erythropoietin. Am] Nephrol 1989;9:353-62.
- Schaefer RMl, Leschke M, Strauer BE, Heidland A. Blood rheology and hypertension in hemodialysis patients treated with erythropoictin. AmJNephrol 1988;8:449-53.
- Scientific discussion. European Medicines Agency 2007;1–26
- Stone WJ, Graber SE, Krantz SB, et al. Treatment of the anemia of predialysis patients with recombinant human erythropoietin: a randomised, placebo-controlled trial. Am]f Med Sci 1988;2:171-9.
- Strickland ID, Chaput de Saintonge DM, Boulton FE, Francis B, Ruobikosa F, Waters JI. 'I'he therapeutic equivalence of oral and intravenous iron in renal dialvsis patients. Clin Nephrol 1977;7:55-7.
- Strickland ID, Chaput de Saintonge DMi, Boulton FE, ei al. A trial of oral iron in dialysis patients. Clin Nephrol 1974;2:13-7.
- Van Wyck DB, Stivelman JC, Ruiz J, Kirlin LF, Katz MA, Ogden DA. Iron status in patients receiving erythropoietin for dialysis-associated anemia. Kidney Int 1989,35:712-6.
- Winearls CG, Oliver DO, Pippard MJ, Reid C, Downing MR, Cotes PM. Effect of human erythropoietin derived from recombinant DNA on the anaemia of patients maintained by chronic haemodialysis. Lancet 1986;ii: 1 175-8.
- Winearls CG. Erythropoietin. Nephrol Dial Tranplant 1989;4:323-6.
- Wizemann V, Comparison of the therapeutic effects of epoetin zeta to epoetin alfa in the maintenance phase of renal anaemia treatment. Curr Med Res Opin. 2008 Mar;24(3):625-37.

19. APPENDICES

APPENDIX I. DECLARATION OF HELSINKI

A. INTRODUCTION

1. 1. The World Medical Association (WMA) has developed the Declaration of Helsinki as a statement of ethical principles for medical research involving human subjects**,** including research on identifiable human material and data. The Declaration is intended to be read as a whole and each of its constituent paragraphs should not be applied without consideration of all other relevant paragraphs.
2. Although the Declaration is addressed primarily to physicians, the WMA encourages other participants in medical research involving human subjects to adopt these principles.
3. It is the duty of the physician to promote and safeguard the health of patients, including those who are involved in medical research. The physician's knowledge and conscience are dedicated to the fulfilment of this duty.
4. The Declaration of Geneva of the WMA binds the physician with the words, "The health of my patient will be my first consideration," and the International Code of Medical Ethics declares that, "A physician shall act in the patient's best interest when providing medical care."
5. Medical progress is based on research that ultimately must include studies involving human subjects. Populations that are underrepresented in medical research should be provided appropriate access to participation in research.
6. In medical research involving human subjects, the well-being of the individual research subject must take precedence over all other interests.
7. The primary purpose of medical research involving human subjects is to understand the causes, development and effects of diseases and improve preventive, diagnostic and therapeutic interventions (methods, procedures and treatments). Even the best current interventions must be evaluated continually through research for their safety, effectiveness, efficiency, accessibility and quality.
8. In medical practice and in medical research, most interventions involve risks and burdens.
9. Medical research is subject to ethical standards that promote respect for all human subjects and protect their health and rights. Some research populations are particularly vulnerable and need special protection. These include those who cannot give or refuse consent for themselves and those who may be vulnerable to coercion or undue influence.
10. Physicians should consider the ethical, legal and regulatory norms and standards for research involving human subjects in their own countries as well as applicable international norms and standards. No national or international ethical, legal or regulatory requirement should reduce or eliminate any of the protections for research subjects set forth in this Declaration.

B. PRINCIPLES FOR ALL MEDICAL RESEARCH

11. It is the duty of physicians who participate in medical research to protect the life, health, dignity, integrity, right to self-determination, privacy, and confidentiality of personal information of research subjects.
12. Medical research involving human subjects must conform to generally accepted scientific principles, be based on a thorough knowledge of the scientific literature, other relevant sources of information, and adequate laboratory and, as appropriate, animal experimentation. The welfare of animals used for research must be respected.
13. Appropriate caution must be exercised in the conduct of medical research that may harm the environment.
14. The design and performance of each research study involving human subjects must be clearly described in a research protocol. The protocol should contain a statement of the ethical considerations involved and should indicate how the principles in this Declaration have been addressed. The protocol should include information regarding funding, sponsors, institutional affiliations, other potential conflicts of interest, incentives for subjects and provisions for treating and/or compensating subjects who are harmed as a consequence of participation in the research

study. The protocol should describe arrangements for post-study access by study subjects to interventions identified as beneficial in the study or access to other appropriate care or benefits.

15. The research protocol must be submitted for consideration, comment, guidance and approval to a research ethics committee before the study begins. This committee must be independent of the researcher, the sponsor and any other undue influence. It must take into consideration the laws and regulations of the country or countries in which the research is to be performed as well as applicable international norms and standards but these must not be allowed to reduce or eliminate any of the protections for research subjects set forth in this Declaration. The committee must have the right to monitor ongoing studies. The researcher must provide monitoring information to the committee, especially information about any serious adverse events. No change to the protocol may be made without consideration and approval by the committee.
16. Medical research involving human subjects must be conducted only by individuals with the appropriate scientific training and qualifications. Research on patients or healthy volunteers requires the supervision of a competent and appropriately qualified physician or other health care professional. The responsibility for the protection of research subjects must always rest with the physician or other health care professional and never the research subjects, even though they have given consent.
17. Medical research involving a disadvantaged or vulnerable population or community is only justified if the research is responsive to the health needs and priorities of this population or community and if there is a reasonable likelihood that this population or community stands to benefit from the results of the research.
18. Every medical research study involving human subjects must be preceded by careful assessment of predictable risks and burdens to the individuals and communities involved in the research in comparison with foreseeable benefits to them and to other individuals or communities affected by the condition under investigation.
19. Every clinical trial must be registered in a publicly accessible database before recruitment of the first subject.
20. Physicians may not participate in a research study involving human subjects unless they are confident that the risks involved have been adequately assessed and can be satisfactorily managed. Physicians must immediately stop a study when the risks are found to outweigh the potential benefits or when there is conclusive proof of positive and beneficial results.
21. Medical research involving human subjects may only be conducted if the importance of the objective outweighs the inherent risks and burdens to the research subjects.
22. Participation by competent individuals as subjects in medical research must be voluntary. Although it may be appropriate to consult family members or community leaders, no competent individual may be enrolled in a research study unless he or she freely agrees.
23. Every precaution must be taken to protect the privacy of research subjects and the confidentiality of their personal information and to minimize the impact of the study on their physical, mental and social integrity.
24. In medical research involving competent human subjects, each potential subject must be adequately informed of the aims, methods, sources of funding, any possible conflicts of interest, institutional affiliations of the researcher, the anticipated benefits and potential risks of the study and the discomfort it may entail, and any other relevant aspects of the study. The potential subject must be informed of the right to refuse to participate in the study or to withdraw consent to participate at any time without reprisal. Special attention should be given to the specific information needs of individual potential subjects as well as to the methods used to deliver the information. After ensuring that the potential subject has understood the information, the physician or another appropriately qualified individual must then seek the potential subject's freely-given informed consent, preferably in writing. If the consent cannot be expressed in writing, the non-written consent must be formally documented and witnessed.
25. For medical research using identifiable human material or data, physicians must normally seek consent for the collection, analysis, storage and/or reuse. There may be situations where consent would be impossible or impractical to obtain for such research or would pose a threat to the

validity of the research. In such situations the research may be done only after consideration and approval of a research ethics committee.

26. When seeking informed consent for participation in a research study the physician should be particularly cautious if the potential subject is in a dependent relationship with the physician or may consent under duress. In such situations the informed consent should be sought by an appropriately qualified individual who is completely independent of this relationship.
27. For a potential research subject who is incompetent, the physician must seek informed consent from the legally authorized representative. These individuals must not be included in a research study that has no likelihood of benefit for them unless it is intended to promote the health of the population represented by the potential subject, the research cannot instead be performed with competent persons, and the research entails only minimal risk and minimal burden.
28. When a potential research subject who is deemed incompetent is able to give assent to decisions about participation in research, the physician must seek that assent in addition to the consent of the legally authorized representative. The potential subject's dissent should be respected.
29. Research involving subjects who are physically or mentally incapable of giving consent, for example, unconscious patients, may be done only if the physical or mental condition that prevents giving informed consent is a necessary characteristic of the research population. In such circumstances the physician should seek informed consent from the legally authorized representative. If no such representative is available and if the research cannot be delayed, the study may proceed without informed consent provided that the specific reasons for involving subjects with a condition that renders them unable to give informed consent have been stated in the research protocol and the study has been approved by a research ethics committee. Consent to remain in the research should be obtained as soon as possible from the subject or a legally authorized representative.
30. Authors, editors and publishers all have ethical obligations with regard to the publication of the results of research. Authors have a duty to make publicly available the results of their research on human subjects and are accountable for the completeness and accuracy of their reports. They should adhere to accepted guidelines for ethical reporting. Negative and inconclusive as well as positive results should be published or otherwise made publicly available. Sources of funding, institutional affiliations and conflicts of interest should be declared in the publication. Reports of research not in accordance with the principles of this Declaration should not be accepted for publication.

C. ADDITIONAL PRINCIPLES FOR MEDICAL RESEARCH COMBINED WITH MEDICAL CARE

31. The physician may combine medical research with medical care only to the extent that the research is justified by its potential preventive, diagnostic or therapeutic value and if the physician has good reason to believe that participation in the research study will not adversely affect the health of the patients who serve as research subjects.
32. The benefits, risks, burdens and effectiveness of a new intervention must be tested against those of the best current proven intervention, except in the following circumstances:
    - The use of placebo, or no treatment, is acceptable in studies where no current proven intervention exists; or
    - Where for compelling and scientifically sound methodological reasons the use of placebo is necessary to determine the efficacy or safety of an intervention and the patients who receive placebo or no treatment will not be subject to any risk of serious or irreversible harm. Extreme care must be taken to avoid abuse of this option.
33. At the conclusion of the study, patients entered into the study are entitled to be informed about the outcome of the study and to share any benefits that result from it, for example, access to interventions identified as beneficial in the study or to other appropriate care or benefits.

34. The physician must fully inform the patient which aspects of the care are related to the research. The refusal of a patient to participate in a study or the patient's decision to withdraw from the study must never interfere with the patient-physician relationship.
35. In the treatment of a patient, where proven interventions do not exist or have been ineffective, the physician, after seeking expert advice, with informed consent from the patient or a legally authorized representative, may use an unproven intervention if in the physician's judgment it offers hope of saving life, re-establishing health or alleviating suffering. Where possible, this intervention should be made the object of research, designed to evaluate its safety and efficacy. In all cases, new information should be recorded and, where appropriate, made publicly available.

APPENDIX II. IRON STATUS AND THERAPY

	Hb, Epoetin and iron status	Hb, Epoetin and iron management during titration phase
1	Hb in target range (10 to 12 g/dl) and adequate iron store (serum ferritin level > 100)	Continue on usual Epoetin dose and observe Hb for 4 weeks. Patients with stable Hb maintained between 10 and 12 g/dl, and on stable Epoetin dose will proceed to enter the maintenance phase at the end of the baseline period.<br><br>Hb levels measured during this observation period will be considered as baseline level.<br><br>In the event a patient's Hb were to decline below 10 g/dl during the observation period, patients should switch to titration phase as described below
2	Hb below target range (< 10) and marginal iron store (all patients will have serum ferritin level > 100 to be eligible for entry)	Increase Epoetin dose during titration phase, and give IV iron as appropriate.<br><br>For patients unable to access IV iron therapy, sponsor will support the iron therapy.
3	Hb below target range (< 10) and serum ferritin level > 500	Increase Epoetin dose during titration and monitor iron closely. If serum ferritin were to decline rapidly, giving IV iron should be considered appropriately.<br><br>For patients unable to access IV iron therapy, sponsor will support the iron therapy.

APPENDIX III. EPOETIN DOSING

	Weekly dose and Dosing (regimen)	Weekly dose and Dosing after 25% increase	Weekly dose and Dosing after 25% decrease
1.	2000 IU given as 2000 IU 1X a week	3000 IU given as alternative dosage (e.g. 2000 IU/1st week, 4000 IU/2nd Week)	1000 IU given as alternative dosage (e.g. no injection in 1st week, 2000 IU/2nd Week)
2.	3000 IU given as alternative dosage (e.g. 2000 IU/1st week, 4000 IU/2nd Week)	4000 IU given as 2000 IU 2X a week	2000 IU given as 2000 IU 1X a week
3.	4000 IU given as 2000 IU 2X/week	5000 IU given as alternative dosage (e.g. 4000 IU/1st week, 6000 IU/2nd Week)	3000 IU given as alternative dosage (e.g. 2000 IU/1st week, 4000 IU/2nd Week)
4.	5000 IU given as alternative dosage (e.g. 4000 IU/1st week, 6000 IU/2nd Week	6000 IU given as 2000 IU 3X a week	4000 IU given as 2000 IU 2X a week
5.	6000 IU given as 2000 IU 3X/week	8000 IU given as 4000 IU 2X a week	4000 IU given as 2000 IU 2X/week
6.	7000 IU given as alternative dosage (e.g. 6000 IU/1stweek, 8000 IU/2nd week)	9000 IU given as alternative dosage (e.g. 8000 IU/1st week, 10,000 IU/2nd week)	5000 IU given as alternative dosage (e.g. 4000 IU/1st week, 6000 IU/2nd Week
7.	8000 IU given as 4000 IU 2X/week	10000 IU given as 4000 IU, 4000 IU and 2000 IU during a week	6000 IU given as 2000 IU 3X/week
8.	9000 IU given as alternative dosage (e.g. 8000 IU/1st week, 10,000 IU/2nd week)	11,000 IU given as alternative dosage (e.g. 10,000 IU/1st week, 12,000 IU/2nd week)	7000 IU given as alternative dosage (e.g. 6000 IU/1stweek, 8000 IU/2nd week)
9.	10,000 IU given as 4000 IU, 4000 IU and 2000 IU during a week	12,000 IU given as 4000 IU 3X/ week	8000 IU given as 4000 IU 2X/week
10.	11,000 IU given as alternative dosage (e.g. 10,000 IU/1st week, 12,000 IU/2nd week)	14,000 IU given as 6000 IU, 4000 IU and 4000 IU during a week	8000 IU given as 4000 IU 2X/week
11.	12,000 IU given as 4000 IU 3X / week	15,000 IU given as alternative dosage (e.g. 6000 IU, 6000 IU and 2000 IU/1st week, 6000IU, 6000IU and 4000IU/2nd week)	9000 IU given as alternative dosage (e.g. 8000 IU/1st week, 10,000 IU/2nd week)

APPENDIX IV. LIST OF STUDY PERSONNEL AND CONTACT DETAILS

#	Participating Site(s)	Name of Investigator(s)
1.	Zaki Morad Nephrology Services Sdn Bhd	Dr. Zaki Morad bin Mohamad Zaher
2.	Pusat Dialysis NKF- Yayasan Dialysis, Pertubuhan Pendidikan Akhlak Taiping	Dr. Indralingam Vaithilingam
3.	Hemodialysis Unit, Hospital Taiping	Dr. Indralingam Vaithilingam
4.	Hemodialysis Unit Hospital Pakar Sultanah Fatimah, Muar	Dr. Yia Hua Jern
5.	MULS-NKF Dialysis Centre, Ipoh, Perak	Dr. Loh Chek Loong
6.	Hemodialysis Unit, Hosp.Raja Permaisuri Bainun	Dr. Loh Chek Loong
7.	Hemodialysis Unit, Hospital Melaka	Dr. Korina Rahmat
8.	Unit Hemodialysis Hosp Sultan Hj Ahmad Shah,Termeloh	Dr. Rafidah Abdullah
9.	Mentakab Hemodialysis Unit (Hospital Mentakab Lama)	Dr. Rafidah Abdullah
10.	APEX Club Of Klang NKF Charity Dialisis Centre, Klang	Dr. Rafidah Abdullah
11.	Unit Hemodialysis Hospital Jengka	Dr. Rafidah Abdullah
12.	Quality Dialysis Care (Meru Branch)	Dr Ong Kee Liang
13.	Haemodialysis Unit, Hospital Raja Perempuan Zainab II	Dr. Wan Hasnul Halimi
14.	Pusat Rawatan Dialisis (NKF), Kota Bharu, Kelantan	Dr. Sukeri Mohamed
15.	Pusat Haemodialysis Dr. Ismail Sdn Bhd Alor Setar	Dr. Syed Faisal Bin Taha
16.	Pusat Dialisis NKF - Kelab Lions Alor Setar, Kedah	Dr. Ching Chen Hua
17.	Pusat Rawatan Dialisis Fungates Superflow, NKF Kepong	Dr. Bee Boon Cheak
18.	Metro Specialist Hospital, Sungai Petani, Kedah	Dr. Gan Hwa Chau
19.	Quality Dialysis Care Sdn Bhd (Cheras Branch)	Dr. Ng Eng Khim
20.	Yayasan Kebajikan SSL Haemodialysis, Petaling Jaya	Dr. Lim Soo Kun
21.	Daycare clinic, haemodialysis Unit, Hospital Tuanku Jaafar Seremban	Dr. Lily Mushahar

20. SUMMARY OF PROTOCOL AMENDMENTS AND ADMINISTRATIVE CHANGES TO THE PROTOCOL

Changes made from PG-EPO-Ph3 v2.4 to PG_EPO_Ph3 v2.5

Page #	Old Text	New/Revised Text	Justification
All Footer	Protocol PG-EPO-Ph3__v2.4/15May 2013	Protocol PG-EPO-Ph3_**v2.5_Malaysia/23Jul2013**	Changed version and date
Page 3 of 77	Protocol Agreement<br><br>Blank	Protocol Agreement<br>Note:<br>This study is funded by a grant from CCM Duopharma Biotech Bhd. and Pangen Biotech Inc.	Inserted name of the sponsor (MREC comment)
Page 5 & page 25 of 77	**Inclusion Criteria**<br>7. Patients must have the following at Screening Visit or prior to randomization and have the baseline period (or observation period)	**Inclusion Criteria**<br>7. Patients must have the following at Screening Visit or prior to randomization as well as at the baseline period (or observation period)	Grammatical error correction (MREC comment)
Page 7 of 77	**Titration phase**<br>• If Hb increase past 2 week is ≤ 1.0 g/dl and Hb level is in the target range (10 – 12 g/dl), the dose will be maintained.<br><br>When a change in Eprex® dose is indicated, a one-step change (25% of current dosage) is performed based on the stepwise dosing schedule refer to appendix II.<br><br>During last four weeks of the titration period, the Hb level will be evaluated to meet the inclusion criteria to conduct the randomization as same as observation period.	**Titration phase**<br>• If Hb increase in the past 2 weeks is ≤ 1.0 g/dl and Hb level is in the target range (10 – 12 g/dl), the dose will be maintained.<br><br>When a change in Eprex® dose is indicated, a one-step change (25% of current dosage) is performed based on the stepwise dosing schedule refer to appendix III.<br><br>During last four weeks of the titration period, the Hb level will be evaluated to meet the inclusion criteria to conduct the randomization similar to observation period.	1) Grammatical error correction (MREC comment)<br>2) Typo

Page 8 & page 32 of 77	**Dose adjustment scheme**<br>When a change in Eprex® or PDA10 dose is indicated, a one-step change (25% of current dosage) is performed according to the stepwise dosing schedule refer to appendix. II.	**Dose adjustment scheme**<br>When a change in Eprex® or PDA10 dose is indicated, a one-step change (25% of current dosage) is performed according to the stepwise dosing schedule refer to appendix. III.<br>The dose adjustment scheme in Open-Label Extension phase is same as maintenance phase**,** but hemoglobin will be assessed every 4 weeks.	1) Typo error<br>2) To state the dose adjustment scheme in OLE phase
Page 8 of 77	**Methodology**<br>Eligible subjects will be randomized, using IWRS, to receive one of the two study treatment regimen (PDA10 or Eprex®) in a 1:1 ratio. All subjects are to continue their treatment PDA10 or Eprex®, and to receive study medication 1-3 times weekly intravenously.	**Methodology**<br>Eligible subjects will be randomized, using IWRS, to receive one of the two study treatment regimen (PDA10 or Eprex®) in a 1:1 ratio. All subjects are to continue their treatment PDA10 or Eprex®, and to receive study medication 1-3 times weekly intravenously (refer to Appendix III).	Added reference number of appendix
Page 15 of 77	**5.1 INSTITUTIONAL REVIEW BOARD**<br>In the case of early termination/temporary halt of the study, the Investigator should notify the IRB/IEC within the day to be required, and a detailed written explanation of the reasons for the termination/halt should be given.	**5.1 INSTITUTIONAL REVIEW BOARD**<br>In the case of early termination/temporary halt of the study, the Investigator should notify the IRB/IEC within 24 hours, and a detailed written explanation of the reasons for the termination/halt should be given.	1) Grammatical error correction<br>(MREC comment)
Page 15 of 77	**5.3 SUBJECT INFORMATION AND INFORMED CONSENT**<br>……………………………At every visit during the study, whether adverse events and adverse drug reactions occur and their severity is evaluated through check-up and the appropriate measures are taken.<br><br>The written consent will be received from all the subjects. The Investigator may discuss the existence	**5.3 SUBJECT INFORMATION AND INFORMED CONSENT**<br>………………………………At every visit during the study, patients are to be assessed with adverse events and adverse drug reactions occurred and their severity should be evaluated along with the appropriate measures taken.<br>The written consent will be obtained from all the subjects. The Investigator may discuss with a	1) Grammatical error correction<br>(MREC comment)

	of the study and probability of being part of the study with candidates without obtaining the consent. If the consent to determine eligibility of the subjects for this study including discontinuation of the drug(s) currently administered is performed, it is considered as a part of the study.<br><br>If appropriate basic information is provided for the subjects, the protocol is explained by the Investigator or the clinical research coordinator (CRC) with easy terms and it seems the subject understands the meaning of participating in the study,<br><br>The Investigator should not conduct specially required tests only for the purpose of the study until obtaining the consent from the subject.<br><br>The investigator should retain the list of all the patients considered as a subject and all the subjects who sign the consent form.	prospective subject concerning the study without obtaining consent.<br>When appropriate basic information is provided for the subjects, it is explained by the Investigator in a simple and non technical manner.<br><br>The Investigator should not conduct any study specific tests until informed consent from the subject has been obtained.<br><br>The investigator should retain the list of all the patients considered as a subject who have been screened and all the subjects who signed the consent form.	
Page 17 of 77	**<u>6. INVESTIGATORS AND STUDY ADMINISTRATIVE STRUCTURE</u>**<br>……………….. Blank	**<u>6. INVESTIGATORS AND STUDY ADMINISTRATIVE STRUCTURE</u>**<br>…………… List of study personnel and contract details, please refer to Appendix IV.	Inserted the list of personnel and contact detail.
Page 32 of 77	**<u>10.4.6.1. DRUG INTERATIONS</u>**<br>……………….. Blank	**<u>10.4.6.1. DRUG INTERATIONS</u>**<br>There is no rescue medication or procedure as this is not a placebo controlled trial.	To state any rescue medication<br>(MREC comment)
Page	**Visit 11(Week 28/Withdrawal)**	**Visit 11(Week 28/Withdrawal)**	1) Typo error

48 of 77	• PDA10 or Eprex® administration<br><br>Subjects who are going to participate in the extension phase should sign the ICFs.	• PDA10 administration (subjects who participate in the OLE phase only)<br>Subjects who signed the ICF will perform the next visit. Subject who will NOT be participated in the extension phase should be returned to the standard treatment provided at the hospital/clinic respectively.	2) To state the way of treatment after end of maintain phase (MREC comment)
Page 51 of 77	**Subject Completion**<br>Subjects who complete the Week 28 visit will be offered PDA10 treatment by way of participation in the open-label extension phase. Subjects must sign an informed consent form for the open-label extension phase.	**Subject Completion**<br>Subjects will be asked to participate in the open-label extension (OLE) phase at Visit 1. Among them, subjects who complete the Week 28 will be offered PDA10 treatment.<br><br>After end of open-label extension phase, subject to return to the standard treatment provided at the hospital/clinic respectively.	1) To state process, place and timing for obtaining informed consent (MREC comment)<br>2) To state the way of treatment after end of extension phase (MREC comment)
Page 59 of 77	**12. PUBLICATION AND PRESENTATION**<br>……………….added sentences	**12. PUBLICATION AND PRESENTATION**<br>……………………………..All information obtained from the trial subjects during this study will not be disclosed to anyone else and will be kept confidential. Data of the study results will be investigated only for medical purposes. If the study results are published as necessary, the subject's identity will remain confidential. In addition, subjects have no access to study data and the results of this study.	1) To state the publication policy for protecting the confidentiality of subject personal information (MREC comment)<br>2) To state whether subjects has access to study and data finding (MREC comment)
Page 60 of 77	**14.2. DATA RECORDING AND DOCUMENTATION**<br>Study records (i.e., copies of eCRFs, regulatory documents) will be retained at the study center, along with adequate source documentation, according to KFDA or other regulatory agency and ICH	**14.2. DATA RECORDING AND DOCUMENTATION**<br>Study records (i.e., copies of eCRFs, regulatory documents) will be retained at the study center or the 3rd parties for 15years from the study ends, along with adequate source documentation, according to KFDA or	1) To state duration and means of storage and archival of medical records and study data (MREC comment)<br>2) To state whether study

	requirements.	other regulatory agency and ICH requirements.<br>After period of storage, the study records will be destroyed according to the regulatory guidelines.	data is destroyed after period of storage<br>(MREC comment)
Page 71 of 77	Blank<br>Added appendix	**APPENDIX IV.**<br>**LIST OF STUDY PERSONNEL AND CONTACT DETAILS**	To state name and institution of MOH investigators<br>(MREC comment)

Changes made from PG-EPO-Ph3 v2.5 to PG_EPO_Ph3 v2.6

Page #	Before	After	Reason
All Footer	ProtocolPG-EPO-Ph3_**v2.5_Malaysia/23Jul2013**	Protocol PG-EPO-Ph3_**v2.6_Malaysia/18 Oct 2013**	Changed version and date
Page 5 and 25	2. Synopsis<br>Inclusion criteria 7<br>Hemoglobin level with 10 - 12 g/dl and a stable IV dose of Eprex® without transfusion prior to randomization (A stable IV dose is defined as less than 25% change up or down in weekly dose and no change in frequency of ~~**injections**~~ for the baseline period [observation period])	2. Synopsis<br>Inclusion criteria 7<br>Hemoglobin level with 10 - 12 g/dl and a stable IV dose of Eprex® without transfusion prior to randomization (A stable IV dose is defined as less than 25% change up or down in weekly dose and no change in frequency of **haemodialysis** for the baseline period [observation period])	Error corrected
Page 7 and 30	**Titration Phase**<br>If Hb level increase during the past two weeks is > 1.0 g/dl or Hb level is ~~**over**~~ 12 g/dl, the dose will be decreased by 25% of current dosage.	**Titration Phase**<br>If Hb level increase during the past two weeks is > 1.0 g/dl or Hb level is **approaching** 12 g/dl, the dose will be decreased by 25% of current dosage. **If the hemoglobin continues to increase or exceed 12 g/dl, the treatment should be temporarily withheld until the hemoglobin begins to decrease, at which point therapy should be reinitiated at a dose approximately 25% below the previous dose. If dose reduction is needed, the amount given per dose or the number of weekly injections will be reduced, or both will be reduced.**	Stated that the dose should be decreased if Hb value is approaching 12g/dL according to the EPREX information for approval. (same as maintenance phase)
Page 8 and 31	**Maintenance Phase**<br>The maintenance dose should be individualized and the recommended total weekly dose ~~**is between 75 and**~~ 300 IU/kg.	**Maintenance Phase**<br>The maintenance dose should be individualized and the recommended total weekly dose **should not exceed 300 IU/kg.**	Error corrected (In 10.4.1 section stated that "The maximum dosage should not exceed 300 IU/kg /dose")
Page	**10.5.5.1. Schedule of Procedures/Assessments**	**10.5.5.1. Schedule of Procedures/Assessments**	Extended the window

<table>
<tr><th>Page #</th><th>Before</th><th>After</th><th>Reason</th></tr>
<tr><td>42</td><td>Visit window is ±~~3~~ days</td><td>Visit window is ± **5** days</td><td>period considering period of sending the blood sample from Malaysia to the central laboratory in Korea</td></tr>
<tr><td>Page 45</td><td>Visit 4 and Randomization (Baseline, Week 0)
● PDA10 or Eprex® administration</td><td>Visit 4 and Randomization (Baseline, Week 0)
● PDA10 or Eprex® administration **(Post randomization)**</td><td>Added words for clarification</td></tr>
<tr><td>Page 70</td><td>

Appendix III Epoetin Dosing

<table>
<tr><th></th><th>Weekly dose and Dosing (regimen)</th><th>Weekly dose and Dosing after 25% increase</th><th>Weekly dose and Dosing after 25% decrease</th></tr>
<tr><td>12</td><td>~~3000 IU given as alternative dosage (e.g. 2000 IU/1st week, 4000 IU/2nd Week)~~</td><td>~~4000 IU given as 2000 IU 2X a week~~</td><td>~~2000 IU given as 2000 IU 1X a week~~</td></tr>
<tr><td>13</td><td>New sentence added</td><td>New sentence added</td><td>New sentence added</td></tr>
<tr><td>14</td><td>4000 IU given as 2000 IU 2X/week</td><td>5000 IU given as ~~2000 IU, 2000 IU and 1000 IU during a week~~</td><td>3000 IU given as alternative dosage (e.g. 2000 IU/1st week, 4000 IU/2nd Week)</td></tr>
</table>

</td><td>

Appendix III Epoetin Dosing

<table>
<tr><th></th><th>Weekly dose and Dosing (regimen)</th><th>Weekly dose and Dosing after 25% increase</th><th>Weekly dose and Dosing after 25% decrease</th></tr>
<tr><td>1.</td><td>2000 IU given as 2000 IU 1X a week</td><td>3000 IU given as alternative dosage (e.g. 2000 IU/1st week, 4000 IU/2nd Week)</td><td>1000 IU given as alternative dosage (e.g. no injection in 1st week, 2000 IU/2nd Week)</td></tr>
<tr><td>2.</td><td>Added
3000 IU given as alternative dosage (e.g. 2000 IU/1st week, 4000 IU/2nd Week)</td><td>Added
4000 IU given as 2000 IU 2X a week</td><td>Added
2000 IU given as 2000 IU 1X a week</td></tr>
<tr><td>3.</td><td>No change</td><td>5000 IU given as alternative dosage (e.g. 4000 IU/1st week, 6000 IU/2nd Week)</td><td>No change</td></tr>
</table>

</td><td>1) Error correction
2) Stated additional guideline regarding increase/decrease dosage from 2000IU</td></tr>
<tr><td>Page71</td><td>**Appendix IV**
States 22 participating sites with 11 Principal</td><td>**Appendix IV**
State 18 participating sites with 13 Principal</td><td>1) Sites that withdraw because cant fulfill the</td></tr>
</table>

<table>
<tr><th>Page #</th><th>Before</th><th>After</th><th>Reason</th></tr>
<tr>
<td></td>
<td>Investigators in Malaysia
<table>
<tr><th>#</th><th>Participating Site(s)</th><th>Name of Investigator(s)</th></tr>
<tr><td>1.</td><td>Zaki Morad Nephrology Services Sdn Bhd</td><td>Dr. Zaki Morad bin Mohamad Zaher</td></tr>
<tr><td>2.</td><td>Pusat Dialysis NKF-Yayasan Dialysis, Pertubuhan Pendidikan Akhlak Taiping,</td><td rowspan="2">Dr. Indralingam Vaithilingam</td></tr>
<tr><td>3.</td><td>Hemodialysis Unit, Hospital Taiping,</td></tr>
<tr><td>4.</td><td>Hemodialysis Unit Hospital Pakar Sultanah Fatimah, Muar</td><td>Dr. Yia Hua Jern</td></tr>
<tr><td>5.</td><td>MULS-NKF Dialysis Centre, Ipoh, Perak</td><td rowspan="2">Dr. Loh Chek Loong</td></tr>
<tr><td>6.</td><td>Hemodialysis Unit, Hosp.Raja Permaisuri Bainun,</td></tr>
<tr><td>7.</td><td>Hemodialysis Unit, Hospital Melaka</td><td rowspan="3">Dr. Korina Rahmat</td></tr>
<tr><td>~~8.~~</td><td>~~Hemodialisis Yakin Jaya, Melaka~~</td></tr>
<tr><td>~~9.~~</td><td>~~Alor Gajah Dialysis~~</td></tr>
</table>
</td>
<td>Investigators in Malaysia
<table>
<tr><th>#</th><th>Participating Site(s)</th><th>Name of Investigator(s)</th></tr>
<tr><td>1.</td><td>Zaki Morad Nephrology Services Sdn Bhd</td><td>Dr. Zaki Morad bin Mohamad Zaher</td></tr>
<tr><td>2.</td><td>Pusat Dialysis NKF-Yayasan Dialysis, Pertubuhan Pendidikan Akhlak Taiping,</td><td rowspan="2">Dr. Indralingam Vaithilingam</td></tr>
<tr><td>3.</td><td>Hemodialysis Unit, Hospital Taiping,</td></tr>
<tr><td>4.</td><td>Hemodialysis Unit Hospital Pakar Sultanah Fatimah, Muar</td><td>Dr. Yia Hua Jern</td></tr>
<tr><td>5.</td><td>MULS-NKF Dialysis Centre, Ipoh, Perak</td><td rowspan="2">Dr. Loh Chek Loong</td></tr>
<tr><td>6.</td><td>Hemodialysis Unit, Hosp.Raja Permaisuri Bainun,</td></tr>
<tr><td>7.</td><td>Hemodialysis Unit, Hospital Melaka</td><td>Dr. Korina Rahmat</td></tr>
<tr><td>8.</td><td>Unit Hemodialysis Hosp Sultan Hj</td><td>Dr. Rafidah</td></tr>
</table>
</td>
<td>plan enrollment target
2) 3 newly recruited sites added – awaiting for EC approval</td>
</tr>
</table>

<table>
<tr><th>Page #</th><th>Before</th><th>After</th><th>Reason</th></tr>
<tr>
<td></td>
<td>
<table>
<tr><td></td><td>~~Centre D/A Embun Budiman Sdn Bhd,~~</td><td></td></tr>
<tr><td>~~10.~~</td><td>~~Pusat Dialisis Comfort, Melaka~~</td><td>~~Dr Yaw Chong Hwa~~</td></tr>
<tr><td>11.</td><td>Unit Hemodialysis Hosp Sultan Hj Ahmad Shah,Termeloh,</td><td rowspan="4">Dr. Rafidah Abdullah</td></tr>
<tr><td>12.</td><td>Mentakab Hemodialysis Unit (Hospital Mentakab Lama)</td></tr>
<tr><td>13.</td><td>APEX Club Of Klang NKF Charity Dialisis Centre, Klang,</td></tr>
<tr><td>14.</td><td>Unit Hemodialysis Hospital Jengka</td></tr>
<tr><td>~~15.~~</td><td>~~Quality Dialysis Care (Klang Branch)~~</td><td rowspan="3">Dr. Ong Kee Liang</td></tr>
<tr><td>~~16.~~</td><td>~~Quality Dialysis Care (Batang Berjuntai Branch)~~</td></tr>
<tr><td>~~17~~</td><td>~~Quality Dialysis Care (Cheras Branch)~~</td></tr>
</table>
</td>
<td>
<table>
<tr><td></td><td>Ahmad Shah,Termeloh,</td><td rowspan="4">Abdullah</td></tr>
<tr><td>9.</td><td>Mentakab Hemodialysis Unit (Hospital Mentakab Lama)</td></tr>
<tr><td>10.</td><td>APEX Club Of Klang NKF Charity Dialisis Centre, Klang,</td></tr>
<tr><td>11.</td><td>Unit Hemodialysis Hospital Jengka</td></tr>
<tr><td>12.</td><td>Quality Dialysis Care (Meru Branch)</td><td>Dr. Ong Kee Liang</td></tr>
<tr><td>13.</td><td>Haemodialysis Unit, Hospital Raja Perempuan Zainab II</td><td>Dr. Wan Hasnul Halimi</td></tr>
<tr><td>14.</td><td>Pusat Rawatan Dialisis(NKF), Kota Bharu, Kelantan</td><td>Dr. Sukeri Mohamed</td></tr>
<tr><td>15.</td><td>Pusat Haemodialysis Dr. Ismail Sdn Bhd Alor Setar</td><td>Dr. Syed Faisal Bin Taha</td></tr>
<tr><td>16.</td><td>Pusat Dialisis NKF - Kelab Lions Alor Setar, Kedah</td><td>Dr. Ching Chen Hua</td></tr>
<tr><td>17.</td><td>Pusat Rawatan Dialisis Fungates</td><td></td></tr>
</table>
</td>
<td></td>
</tr>
</table>

Page #	Before			After			Reason
	18.	Quality Dialysis Care<br>(Meru Branch)			Superflow, NKF Kepong	Dr. Bee Boon Cheak	
	~~19.~~	~~Quality Dialysis Care<br>(Wangsa Maju Branch)~~		18.	Metro Specialist Hospital,<br>Sungai Petani, Kedah	Dr. Gan Hwa Chau	
	20.	Haemodialysis Unit, Hospital Raja Perempuan Zainab II	Dr. Wan Hasnul Halimi				
	21.	Pusat Rawatan Dialisis(NKF),<br>Kota Bharu,<br>Kelantan	Dr. Sukeri Mohamed				
	22	Pusat Haemodialysis Dr. Ismail Sdn Bhd Alor Setar	Dr. Syed Faisal Bin Taha				

Changes made from PG-EPO-Ph3 v2.6 Malaysia/18 Oct 2013 to PG_EPO_Ph3 v3.0_Malaysia/10 Jun 2014

page	Before	After	Reason
All Footer	Protocol PG_EPO_Ph3_~~**v2.6_Malaysia/18 Oct 2013**~~	Protocol PG_EPO_Ph3_**v3.0_Malaysia/10 Jun 2014**	Changed version and date
Page 5, 25	Exclusion Criteria<br>5. Patients with a current or recent known history of a severe hyperparathyroidism or PTH > ~~8~~00 pg/ml within 12 weeks prior to randomization.	Exclusion Criteria<br>5. Patients with a current or recent known history of a severe hyperparathyroidism or PTH > **15**00 pg/ml within 12 weeks prior to randomization.	Changed PTH value
Page 6, 26	Exclusion Criteria<br>10. Patients with significant inflammation (CRP >~~10~~ mg/L within 12 weeks prior to randomization)	Exclusion Criteria<br>10. Patients with significant inflammation (CRP >**20** mg/L within 12 weeks prior to randomization)	Changed CRP value
Page 7, 31	**Titration phase**<br><br>* The patients will be treated with iron therapy based on iron level as follows;<br>① Marginal iron store (all patients will have serum ferritin level > 100 to be eligible for entry) - Eprex® dose increasing during the titration phase and IV iron ~~**sucrose**~~ as appropriate. For patients unable to access IV iron ~~**sucrose**~~ therapy, ~~**Sponsor will provide alternative iron therapy**~~.<br>② Hb is below 10 g/dl and serum ferritin level > 500 - Eprex® dose increasing during the titration phase and a close iron monitoring. If serum ferritin is to decline rapidly, ~~**IV iron sucrose should be considered and to be provided appropriately**~~. For patients	**Titration phase**<br><br>* The patients will be treated with iron therapy based on iron level as follows;<br>① Marginal iron store (all patients will have serum ferritin level > 100 to be eligible for entry) - Eprex® dose increasing during the titration phase and IV iron as appropriate. For patients unable to access IV iron therapy, **sponsor will support the iron therapy.**<br>② Hb is below 10 g/dl and serum ferritin level > 500 - Eprex® dose increasing during the titration phase and a close iron monitoring. If serum ferritin is to decline rapidly, **giving** IV iron should be considered appropriately. For patients unable to access IV iron therapy, **sponsor will support the iron therapy.**	Due to the fact that Dextran or sucrose could be prescribed as an IV iron agent in Malaysia, the word of 'sucrose' is deleted. Thus, all IV iron agents can be co-administrated.

page	Before	After	Reason
	unable to access IV iron sucrose therapy, ~~**Sponsor will provide alternative iron therapy**~~		
Page 24	**10.2Discussion of Study Design, Including the Choice of Control Groups**<br><br>Based on the above, intravenous iron ~~**sucrose**~~ will be supplemented with PDA10 and Eprex® as appropriate. See Appendix II for iron status and therapy.	**10.2Discussion of Study Design, Including the Choice of Control Groups**<br><br>Based on the above, intravenous iron **agent** will be supplemented with PDA10 and Eprex® as appropriate. See Appendix II for iron status and therapy.	Due to the fact that Dextran or sucrose could be prescribed as an IV iron agent in Malaysia, the word of 'sucrose' is deleted. Thus, all IV iron agents can be co-administrated.
Page 26	~~**20.Patients with known sensitivity to mammalian cell derived products**~~	**20. Patients who currently are pregnant or lactating**	See below no.1
Page 27	10.3.4 ~~Withdrawal~~ From Therapy or Assessment<br>..(skip)..<br>~~Subjects who withdraw from study will not be replaced.~~	10.3.4 **Discontinuation** From Therapy or Assessment<br>..(skip)..<br>(delete)	1. Word error (this section 10.3.4 states about discontinuation from this study.)<br><br>2. Duplication of the sentence with the Section 10.3.5
Page 27	10.3.5 Replacement Procedures<br>The subjects who were ~~withdrawn~~ at the screening after signing the informed consent form can be re-screened in this study if they ~~are~~ were not randomized. At this time, they must sign a new informed consent form.<br>If the randomized subject is withdrawn, he/she is not replaced with a new subject. The withdrawn subject's randomization number cannot be used again and the subject in question cannot participate in this study again.<br>~~Subjects in this study who prematurely~~	10.3.5 Replacement Procedures<br>The subjects who were **discontinued** at the screening after signing the informed consent form can be re-screened in this study if they were not yet randomized. At this time, they must sign a new informed consent form.<br>If the randomized subject is withdrawn, he/she is not replaced with a new subject. The withdrawn subject's randomization number cannot be used again and the subject in question cannot participate in this study again.<br>(delete)	

<table>
<tr><th>page</th><th>Before</th><th>After</th><th>Reason</th></tr>
<tr><td></td><td>~~discontinue treatment will not be replaced.~~</td><td></td><td></td></tr>
<tr><td>Page 38</td><td>~~**ransferritin**~~</td><td>**transferrin**</td><td>Word error</td></tr>
<tr><td>Page 42</td><td>**10.5.5.1. Schedule of Procedures/Assessments**

8) Visit window is ± ~~**5**~~ days.</td><td>**10.5.5.1. Schedule of Procedures/Assessments**

8) Visit window is ±**7** days.</td><td>Considering the period of sending the blood samples to the central laboratory in Korea from Malaysia, the window period is changed.</td></tr>
<tr><td>Page 69</td><td>**Appendix II. Iron Status and Therapy**
<table>
<tr><td>2</td><td>Hb below target range (< 10) and marginal iron store (all patients will have serum ferritin level > 100 to be eligible for entry)</td><td>Increase Epoetin dose during titration phase, and give IV iron ~~**sucrose**~~ as appropriate.

For patients unable to access IV iron ~~**sucrose**~~ therapy, sponsor will ~~**consider supporting such patients'**~~ iron therapy</td></tr>
<tr><td>3</td><td>Hb below target range (< 10) and serum ferritin level > 500</td><td>Increase Epoetin dose during titration and monitor iron closely. If serum ferritin were to decline rapidly, ~~**consider giving IV iron sucrose as appropriate**~~.

For patients unable to</td></tr>
</table></td><td>**Appendix II. Iron Status and Therapy**
<table>
<tr><td>2</td><td>Hb below target range (< 10) and marginal iron store (all patients will have serum ferritin level > 100 to be eligible for entry)</td><td>Increase Epoetin dose during titration phase, and give IV iron as appropriate.

For patients unable to access IV iron therapy, **sponsor will support the iron therapy.**</td></tr>
<tr><td>3</td><td>Hb below target range (< 10) and serum ferritin level > 500</td><td>Increase Epoetin dose during titration and monitor iron closely. If serum ferritin were to decline rapidly, **giving IV iron should be considered appropriately.**</td></tr>
</table></td><td>Due to the fact that Dextran or sucrose could be prescribed as an IV iron agent in Malaysia, the word of 'sucrose' is deleted. Thus, all IV iron agents can be co-administrated.</td></tr>
</table>

page	Before	After	Reason
	access IV iron sucrose therapy, sponsor will consider supporting such patients' iron therapy	For patients unable to access IV iron therapy, **sponsor will support the iron therapy.**	

1. Reason

Initially during the draft protocol development in English version, Study Exclusion Criteria # 20 'Patients who currently are pregnant or lactating' and Study exclusion criteria # 21 'Patients with known sensitivity to mammalian cell derived products' were both included in the draft Study Protocol. However, because PDA10 is developed by culturing animal cells, the criteria of 'patients with known sensitivity to mammalian cell derived products' was very much similar with the exclusion criteria # 18 of 'Patients with hypersensitivity to the active substance or to any of the Excipient'. Hence, the criteria of 'Patients with known sensitivity to mammalian cell derived products' was to be deleted.

The exclusion criteria # 21 i.e. 'patients with known sensitivity to mammalian cell derived products' was deleted in the synopsis but unfortunately, the exclusion criteria # 20 i.e. 'patients who currently are pregnant or lactating' was deleted in the body. For the reason above, it was decided to delete the exclusion Criteria # 20 i.e. 'Patients with known sensitivity to mammalian cell derived products" and to be replaced with criteria of 'Patients who currently are pregnant or lactating' in the body.

Changes made from PG-EPO-Ph3 v3.0_Malaysia/10 Jun 2014 to PG_EPO_Ph3 v3.0_Malaysia01/07 Jul 2014

page	Before	After	Reason
All Footer	Protocol PG_EPO_Ph3_ ~~v3.0_Malaysia/10 Jun 2014~~	Protocol PG_EPO_Ph3_ **v3.0_Malaysia01/07 Jul 2014**	Changed version and date
Page 42	**10.5.5.1. Schedule of Procedures/Assessments**<br><br>8) Visit window is ± ~~7~~ days.	**10.5.5.1. Schedule of Procedures/Assessments**<br><br>8) Visit window is ±5 days. **However, V4 blood sampling is allowed at -7 days from planned V4 date**.	Considering the period of sending the blood samples to the central laboratory in Korea from Malaysia, the window period of blood sampling at V4 (randomization) is added, and the other visit window is changed to 5 days.

Changes made from PG-EPO-Ph3 v3.0_ Malaysia01/07 Jul 2014 to PG_EPO_Ph3 v3.1_Malaysia/07 Nov 2014

page	Before	After	Reason
All Footer	Protocol PG_EPO_Ph3_ ~~**v3.0_Malaysia01/07 Jul 2014**~~	Protocol PG_EPO_Ph3_**v3.1_Malaysia/07 Nov 2014**	Changed version and date
5, 25	**Inclusion Criteria**<br>7. ..(skip)..<br>• Hemoglobin level with 10 - 12 g/dl and a stable IV dose of Eprex® without transfusion prior to randomization (A stable IV dose is defined as ~~**less**~~ than 25% change up or down in weekly dose and no change in frequency of haemodialysis for the baseline period [observation period])<br>• ..(skip)..<br>• Serum ferritin level at least 100 ng/ml and transferrin saturation (TSAT) at least 20% prior to randomization	**Inclusion Criteria**<br>7. ..(skip)..<br>• Hemoglobin level with 10 - 12 g/dl and a stable IV dose of Eprex® without transfusion prior to randomization (A stable IV dose is defined as **not more** than 25% change up or down in weekly dose and no **clinically relevant** change **of regimen** in frequency of haemodialysis for the baseline period [observation period])<br>• Serum ferritin level at least 100 ng/ml and**/or** transferrin saturation (TSAT) at least 20% prior to randomization	1) To clarify the concept of 'the change in frequency of haemodialysis', the explanation was changed to 'relevant change of dialysis regimen'.<br>2)Error correction (Changed this criteria based on EPO biosimilar Binocrit study performed in Europe<br><br>*Reference (additional document 1): Binocrit® Clinical Trial in website of clinical trial.gov (NCT00666835) : Serum ferritin >=100ug/L and/or saturated transferrin levels>=20%
5,25	**Exclusion Criteria**<br>2. Patients with uncontrolled hypertension, defined as a pre-dialysis ~~**systolic blood pressure (BP) > 170mmHg and/or**~~ diastolic BP of greater than 110 within 12 weeks prior to randomization	**Exclusion Criteria**<br>2. Patients with uncontrolled hypertension, defined as a pre-dialysis diastolic BP of greater than 110 within 12 weeks prior to randomization	Changed this criteria based on EPO biosimilar Binocrit study performed in Europe<br><br>*Reference (additional document 1): Binocrit® Clinical Trial in website of clinical trial.gov (NCT00666835) : Uncontrolled hypertension, defined as a predialysis diastolic BP measurement >= 110mmHg during the screening period
5,25	**Exclusion Criteria**	**Exclusion Criteria**	1) Included 'unstable angina' in a

page	Before	After	Reason
	3. Patients with ~~**a current or recent history of thrombotic vascular events, (including but not limited to**~~ stroke, transient ischemic attack, myocardial infarction, cerebrovascular accident, coronary artery ~~**disease, and**~~ deep venous thrombosis~~**) and**~~ decompensate congestive heart failure (New York Heart Association [NYHA] class IV) ~~**within the past 5 years**~~	3. Patients with **severe diseases within the last 6 months prior to randomization (e.g.** stroke, transient ischemic attack, myocardial infarction, cerebrovascular accident, coronary artery **bypass graft**, deep venous thrombosis, **unstable angina,** decompensate congestive heart failure (New York Heart Association [NYHA] class **III**~IV)**, or other thromboembolic events)**	category of severe disease<br>2) To clarify the word 'current or recent', the word was changed to 'within the last 6 months prior to randomization'<br>3) In case of decompensate congestive heart failure, NYHA class III was included in Exclusion criteria, and the period of the disease was changed to 'within the last 6 months prior to randomization' from 'the past 5 years'.<br>4) Included 'other thromboembolic events' to exclude any relevant vascular obstructive diseases due to thrombosis.
5, 26	**Exclusion Criteria**<br>8. Patients with malnutrition (serum albumin < 3.5g/dl)	**Exclusion Criteria**<br>8. Patients with malnutrition (serum albumin < 3.5g/dl **prior to randomization**)	To clarify the date of assessment
6, 26	**Exclusion Criteria**<br>9. Patients with an acute infection, ~~**viral infection**~~ or HIV infection	**Exclusion Criteria**<br>9. Patients with an acute infection, **acute hepatitis (including A, B, C type) or chronic hepatitis B or C requiring treatment,** or HIV infection	To clarify 'viral infection', changed and stated as 'acute hepatitis infection (including A,B,C type) or chronic hepatitis B or C requiring treatment'.
6, 26	**Exclusion Criteria**<br>10. Patients with significant inflammation (CRP >~~2~~0 mg/L within 12 weeks prior to randomization)	**Exclusion Criteria**<br>10. Patients with significant inflammation (CRP >**3**0 mg/L within 12 weeks prior to randomization)	Due to a dietary life and medical environment in Malaysia, CRP values are commonly higher than Korea. Considering these Malaysia's situation, the reference value of CRP was changed.<br>*Reference (additional document 2): According to the EPO Biosimilar Study performed in Europe, the reference value

page	Before	After	Reason
			of CRP in Exclusion criteria was a greater than 100 mg/L (10mg/dL).
6, 26	**Exclusion Criteria**<br>11. Patients with a history of gastrointestinal bleeding	**Exclusion Criteria**<br>11. Patients with a history of gastrointestinal bleeding **within the last 6 months before Screening**	To clarify the date of assessment
6,26	**Exclusion Criteria**<br>16. Patients with a current diagnosis of anemia due to folic acid and/or Vitamin B12 deficiencies, hemolysis, or gastrointestinal bleeding or a history of~~/or~~ active blood or bleeding disorders	**Exclusion Criteria**<br>16. Patients with a current diagnosis of anemia due to folic acid and/or Vitamin B12 deficiencies, hemolysis, or gastrointestinal bleeding or a history of active blood or bleeding disorders **within the last 6 months before Screening**	1)To clarify the date of assessment<br>2)Grammatical error correction
7,30	**10.4.4 Selection of Dosages in the Study**<br>…(skip)..<br>• Criteria to proceed the maintenance phase without titration phase<br>Patients on Eprex® treatment for at least 12 weeks and with hemoglobin within the target range of 10.0 to 12.0 g/dl and a stable IV dose of Eprex® , and adequate iron store (serum ferritin level ~~>~~ 100) in observation period will proceed to enter the maintenance phase.	**10.4.4 Selection of Dosages in the Study**<br>…(skip)..<br>• Criteria to proceed the maintenance phase without titration phase<br>Patients on Eprex® treatment for at least 12 weeks and with hemoglobin within the target range of 10.0 to 12.0 g/dl and a stable IV dose of Eprex® , and adequate iron store (serum ferritin level **≧**100**ng/mL and/or transferrin saturation ≥ 20%**) in observation period will proceed to enter the maintenance phase.	1) Error (sign of inequality) correction<br>2) Added the word due to the error correction of Inclusion criteria 7.
71	**APPENDIX IV. LIST OF STUDY PERSONNEL AND CONTACT DETAILS**<br>…(skip)..	**APPENDIX IV. LIST OF STUDY PERSONNEL AND CONTACT DETAILS**<br>…(skip)..<br>**19.** | **Quality Dialysis** | **Dr. Ng Eng**	Added investigational sites

<table>
<tr><th>page</th><th>Before</th><th>After</th><th>Reason</th></tr>
<tr><td></td><td></td><td><table>
<tr><td></td><td>Care Sdn Bhd (Cheras Branch)</td><td>Khim</td></tr>
<tr><td>20.</td><td>Yayasan Kebajikan SSL Haemodialysis, Petaling Jaya</td><td>Dr. Lim Soo Kun</td></tr>
<tr><td>21.</td><td>Daycare clinic, haemodialysis Unit, Hospital Tuanku Jaafar Seremban</td><td>Dr. Lily Mushahar</td></tr>
</table></td><td></td></tr>
</table>
